# The bacterial community in potato is recruited from soil and partly inherited across generations

**DOI:** 10.1371/journal.pone.0223691

**Published:** 2019-11-08

**Authors:** Franziska Buchholz, Livio Antonielli, Tanja Kostić, Angela Sessitsch, Birgit Mitter

**Affiliations:** Center for Health & Bioresources, Bioresources Unit, AIT Austrian Institute of Technology GmbH, Tulln, Austria; Universite Paris-Sud, FRANCE

## Abstract

Strong efforts have been made to understand the bacterial communities in potato plants and the rhizosphere. Research has focused on the effect of the environment and plant genotype on bacterial community structures and dynamics, while little is known about the origin and assembly of the bacterial community, especially in potato tubers. The tuber microbiota, however, may be of special interest as it could play an important role in crop quality, such as storage stability. Here, we used 16S rRNA gene amplicon sequencing to study the bacterial communities that colonize tubers of different potato cultivars commonly used in Austrian potato production over three generations and grown in different soils. Statistical analysis of sequencing data showed that the bacterial community of potato tubers has changed over generations and has become more similar to the soil bacterial community, while the impact of the potato cultivar on the bacterial assemblage has lost significance over time. The communities in different tuber parts did not differ significantly, while the soil bacterial community showed significant differences to the tuber microbiota composition. Additionally, the presence of OTUs in subsequent tuber generation points to vertical transmission of a subset of the tuber microbiota. Four OTUs were common to all tuber generations and all potato varieties. In summary, we conclude that the microbiota of potato tubers is recruited from the soil largely independent from the plant variety. Furthermore, the bacterial assemblage in potato tubers consists of bacteria transmitted from one tuber generation to the next and bacteria recruited from the soil.

## Introduction

Potato tubers are the fourth most important staple food worldwide after rice, wheat and maize, with an annual production of greater than 382 million tons [1]. Potato tubers provide an excellent source of nutrients and vitamins, but year-round availability depends on industrial scale storage, especially in countries that rely on annual crops [2]. A major challenge in the potato industry is the preservation of tuber quality for up to nine months of storage. Premature sprouting is one of the major causes of loss during postharvest storage of potato tubers [3, 4]. The sprouting behavior of potato tubers depends mainly on the plant genotype [5, 6], but it is also influenced by storage conditions, the type of field soil or environmental factors such as weather during growth [7, 8]. For example, tubers of the same potato variety grown in different fields often show differences in storage stability (personal communication with Austrian potato producers). Apart from the chemical and physical parameters of soil, different field sites differ in the composition of the soil microbiota, which is the main reservoir of microorganisms colonizing plants. This raises the question of to what extent do the microorganisms that colonize potato tubers have an impact on the sprouting behavior of the potato tubers.

Plants host complex communities of archaea, bacteria, and fungi, which live as endophytes in all plants [9]. Those microorganisms that colonize the rhizosphere of plants have received most attention [10]. They represent an important source of microorganisms, which are taken up by plant roots and further colonize the plant interior as endophytes [11].

In the past two decades, research has been dedicated to understanding the bacterial communities in potato plants with the intention to improve agricultural productivity [11–15]. Several biotic factors, such as plant developmental stage [16], plant health and pathogens [17, 18], insects [19], human activities [20] and abiotic factors, such as environmental conditions [21] or soil types [22], are known to influence the structural and functional diversity of the bacterial microbiota of potato plants. In summary, in previous studies, the effect of the plant genotype on the bacterial community structure in potatoes was only minor or not consistent over time or field sites [23, 24], while the soil was identified as the main driver of bacterial community composition in potato plants.

The microbiota of plants is known for their importance for healthy growth and development of the host [25] and was found to play a role, among others, in preventing [26, 27] or favoring rots [17], avoiding quality loss due to, for example, sprouting [28], saccharification [29], water loss [30] or spoilage [31]. Taking this into consideration, it seems likely that the microbial communities that colonize potato tubers influence the behavior of tubers during storage [32]. However, considering that the plant genotype might be the primary factor determining the length of dormancy in potatoes, a deeper understanding of the relationship between plant genotype and potato tuber microbiota is needed before the impact of tuber-colonizing microorganisms on tuber storage stability can be studied.

This study specifically addresses the set-up, community dynamics and heritability of tuber-associated microbiota. We analyzed the bacterial communities associated with tubers of different potato cultivars in subsequent generations and grown in different soils. Initially, seed potatoes of seven potato cultivars were grown in commercial potting soil. At maturity, tubers were harvested and used for 16S rRNA gene amplicon sequencing and planted in pots with five different soil types (commercial potting soil and four different farmland soils). Mature tubers were again used for sequencing-based bacterial community analysis to determine the effect of soil, plant genotype and tuber parts on bacterial community assembly in the potato tuber environment.

## Materials and methods

### Greenhouse experiment

Seven different potato varieties that are used in Austrian potato production were chosen for this analysis. The varieties Lady Claire, Hermes, Agria and Fontane are used for the production of snacks or French fries, and Ditta, Agata and Fabiola are table potatoes.

In February 2016, potato tubers were planted in pots (6 L) filled with commercial potting soil (Profi Substrat, Einheitserde Special, Sinntal-Altengronau, Germany) in the greenhouse. Plants were harvested when growth stage 909 of the BBCH scale was reached [33]. Four varieties (Agata, Fabiola, Hermes and Lady Claire) yielded enough tubers to be further planted in five different soil types. In May 2016, tubers of these four varieties were planted in pots (10 L) filled with a commercial potting soil (Profi Substrat, Einheitserde Special, Sinntal-Altengronau, Germany) or soils collected from fields in three different locations in Austria (Tulln, Karnabrunn and Kettlasbrunn). In Kettlasbrunn, we collected two different soil types from fields in close proximity, i.e., one of the fields was fertilized intensively with manure (personal communication with the farmer) and therefore had a darker color (Kettlasbrunn A) than the other (Kettlasbrunn B). Again, plants were harvested at growth stage 909 of the BBCH scale [33]. In all experiments, we cultivated five replicates per variety and soil type. Before planting, tubers were incubated in 2 ppm gibberellic acid (Sigma-Aldrich, Vienna, Austria) for 20 min to promote rapid germination (adapted from Hartmann et al.) [34]. Afterwards, tubers were dried and planted. During the cultivation period, plants were fertilized twice with 40 g/m^2^ of NPK + trace elements (8% N, 7% P_2_O_5_ and 17% K_2_O) according to the recommendations of the manufacturer (Compo Austria GmbH, Vienna, Austria). The temperature in the greenhouse was set to 22°C with a relative humidity of 50% during the day. During the night, the overall temperature was 21°C with a 35% relative humidity. All plants were grown in separated pots, and each pot was placed into an individual saucer to avoid cross contamination between varieties. Each pot was watered separately from above and kept sufficiently moist. In detail, one to 1.5 L of water was given to each pot every second to third day depending on the temperature in the greenhouse.

### Sampling

Three tubers per variety were randomly selected for microbiome sequencing, which was done directly after receiving the seed potatoes, immediately after harvesting the first generation and directly after harvesting the second generation. This resulted in a total of 102 tubers. Four different parts of each tuber including the corky epidermis, the cortex, the outer medulla and the inner medulla were sampled. In detail, an apple corer was used to extract a core from the tubers, which was then cut to approximately 5 mm thick bud discs (9–10 g). (S1 Fig). All discs were cut into small pieces with a sterile scalpel and mixed with 6 mL of sterile 10% tryptic soy broth (Merck, Germany) in a sterile plastic bag. Microbial cells were dislodged from plant tissue using a Pulsifier (Microgen Bioproducts, Surrey, UK) three times for 15 s. Two mL of the supernatant was collected for subsequent DNA isolation. In the case of the second generation tuber samples, we pooled aliquots of the supernatant of the different tuber parts per tuber to reduce the number of samples. However, the rest of the supernatants were centrifuged to collect the cells, and cell pellets were frozen for later analysis. In addition, 3 x 500 mg (triplicates) of the commercial potting soil used for plant cultivation was frozen at -20°C for DNA extraction.

### DNA isolation

For DNA isolation, samples were centrifuged for 15 min at 10,000 rpm in an Eppendorf™ 5424 Microcentrifuge (Eppendorf, Hamburg, Germany) to pellet microbial cells. Supernatants were discarded, and cell pellets were resuspended in sodium phosphate buffer and transferred to Matrix E of the FastDNA® SPIN Kit for Soil (MP Biomedicals, Solon, OH, USA). Shredding of microbial cells was performed using a bead beater (FastPrep FP 120, Bio101, Savant Instruments, Inc., Holbrook, NY, USA) for 40 s at speed setting 6. DNA extraction was then performed following the manufacturer’s instructions. DNA isolation from soil was also performed with the FastDNA® SPIN Kit for Soil (MP Biomedicals, Solon, OH, USA), strictly following the protocol. DNA (5 μl) was separated and visually tested for quality by electrophoresis (80 V) on 1% (w/v) agarose gels. In addition, DNA concentration was measured with a NanoDrop ND-1000 spectrophotometer (Thermo Fischer Scientific, DE, USA).

### 16S rRNA gene fragment amplicon library preparation and sequencing

A nested PCR approach was used to amplify a 376 bp fragment covering the hypervariable regions V5 to V7 of the 16S rRNA gene [35] as described by Mitter et al. (2017) [36, 37]. PCR conditions were as follows: 50 μl reaction volume that contained 5–10 ng of DNA, 1x KAPA HiFi GC Buffer (5x) with MgCl_2_, 10 mM dNTPs, 10 μM of each primer and 0.5 units of KAPA HiFi DNA polymerase (Kapa Biosystems, Boston, MA, USA). The amplification conditions for all PCRs were as follows: 95°C for 5 min, followed by 25 cycles of amplification at 95°C (30 s), 55°C (30 s) and 72°C (30 s), with a final extension step at 72°C (5 min). PCR was performed in a pegSTAR thermocycler (peQlab, Erlangen, Germany).

For the preparation of 16S rRNA gene amplicon libraries of seed potato tubers and first generation tubers, a first round of PCR was performed using the primer pair 799f (5’-AACMGGATTAGATACCCKG-3’) and 1392r (5’-ACGGGCGGTGTGTRC-3’) [38]. Three independent PCR amplifications were performed per replicate sample and pooled. Pools were loaded onto a 2% agarose gel (w/v) in sterile 1x TAE (Biozym Biotech Trading, Vienna, Austria), and the bacterial 16S rRNA gene amplicons (approximately 600 bp) were separated from mitochondrial 18S rRNA gene amplicons (1000 bp) by electrophoresis for 80 min at 110 V and excised from the gel using X-tracta Gel Extraction Tools (Sigma Aldrich, Vienna, Austria). The gel pieces were collected in the top of a sterile filter tip, which was then placed inside an Eppendorf tube. The PCR product was eluted by centrifugation for 1 min at 1000 rpm in an Eppendorf™ 5424 Microcentrifuge (Eppendorf, Hamburg, Germany). Then, the filter tip was carefully removed with sterile tweezers, and 2 μl of the eluate was used for the second round of PCR with the primer pair 799f (5’-AACMGGATTAGATACCCKG-3’) and 1175r (5’-ACGTCRTCCCCDCCTTCCT-3’) [35] carrying specific barcodes for sample recognition in sequencing (S1 Table). Illumina-adapter ligation and Illumina sequencing (2 x 300 bp) were performed at GATC Biotech AG (Konstanz, Germany). The concentration of PCR amplicons was estimated from the intensity of the amplicon bands on agarose gels with Image Lab™ Software 6.0 (BioRad, Hercules, CA, USA) using the bands of the DNA marker as a point of reference. The amplicons were pooled in equimolar amounts. Afterwards, libraries were purified with the Agencourt® AMPure® XP system (New England Biolabs, Ipswich, MA, USA). The quantity of the library was measured with Quant-iT™ PicoGreen® following the instructions of the manufacturer in a plate reader (BioTek, Winooski, VT, USA).

For the preparation of 16S rRNA gene amplicon libraries of second generation tubers, the same procedure as described above was used with the exception that first PCR was performed with the primer pair 799f and 1175r and second PCR was performed with primers 799f and 1175r carrying specific indices as shown in S2 Table.

For sequencing, pooled and purified libraries of the second generation potato tuber samples were subjected to Illumina adapter ligation and sequencing using 2 x 250 bp MiSeq v2 sequencing at LGC Genomics Berlin, Germany).

For the preparation of 16S rRNA gene amplicon libraries of soil samples, the primer pair

799F_illumina (5´- TCGTCGGCAGCGTCAGATGTGTATAAGAGACAGAACMGGATTAGATACCCKG-3´) and 1175R2_illumina (5’- GTCTCGTGGGCTCGGAGATGTGTATAAGAGACAGACGTCATCCCCACCTTCC-3’) were used with the same PCR conditions as described above and in Samad et al (2016) [39]. After purification of the PCR products with the Agencourt® AMPure® XP system (New England Biolabs, Ipswich, MA, USA), index PCR was performed with Index 1 (N701–N712) and Index 2 Primers (S517–S508) from the Nextera XT Index Kit (Illumina, San Diego, CA, USA). This reaction was carried out with the following settings: 95°C for 3 min, 16 cycles at 95°C for 30 s, 55°C for 30 s, 72°C for 30 s and final extension at 72°C for 5 min. The reaction conditions were as follows: 50 μl reaction volume that contained 50 ng of DNA, 1x KAPA HiFi GC Buffer (5x) with MgCl2, 10 mM dNTPs, 10 μM of each primer and 0.5 units of KAPA HiFi DNA polymerase (Kapa Biosystems, Boston, MA, USA). Afterwards, pooling and purification of amplicons was performed as described above. The amplicon library (4 nM) was denatured using freshly prepared 0.2 N NaOH and diluted to 20 pM using prechilled HT1 buffer (provided by the Illumina MiSeq Reagent Kit v3 for 2x 300 bp PE). A 20% PhiX DNA spike (6 pM) was added as a control to improve the data quality of low diversity samples, and the pool was incubated at 96°C for 2 min in a Thermomixer R (Eppendorf, Hamburg, Germany). Then, the library was immediately placed in an ice-water bath (3 parts ice and 1 part water) for 5 min. Finally, 6 μl of 6 pM denatured DNA was sequenced on the MiSeq instrument (Illumina, San Diego, CA, USA) using the MiSeq Reagent v3 Kit (part number MS-102-3003) with paired end, 2 x 300 bp cycle run, and the Generate FASTQ MiSeq Reporter workflow was applied. Different Illumina sequencing platforms and runs were used for potato tuber and soil samples *inter alia* for cost reasons. In a study by Caporaso et al., it was demonstrated that biological conclusions are consistent across sequencing platforms (the HiSeq2000 versus the MiSeq) and across the sequenced regions of amplicons [40]. However, the sequenced gene region was the same in all sequencing runs of this study.

### 16S rRNA gene sequence processing

Raw data quality was checked in FastQC, and reads were screened for PhiX contamination using Bowtie 2.2.6 [41]. Reads were demultiplexed with a Bayesian demultiplexing algorithm tool [42]. A Bayesian clustering for error correction was applied [43, 44] before merging the PE reads using PEAR 0.9.6 (p<0.001) [45]. Forward and reverse primers were then stripped from merged reads by employing cutadapt 1.8.3 [46] and quality filtering was performed in USEARCH v8.0.1517 (maximum expected error = 0.5) [47, 48]. METAXA2 was used to extract SSU ribosomal reads and to verify the 16S rRNA V5-V7 region of the sequences [49]. Targeted reads were labeled according to the sample name of origin and combined in QIIME [50]. Sequences were dereplicated, sorted and clustered at 97% similarity using VSEARCH 1.1.1 [51]. Chimeras were checked by adopting both de novo and reference-based approached as is standard for the abovementioned tool. The RDP classifier training set v15 (01/2018) was used as a database for reference-based chimera detection. An optimal global alignment was applied afterwards in VSEARCH, and a BIOM table was generated. Taxonomy assignment was performed by employing the naïve Bayesian RDP classifier v2.10 [52] in QIIME using SILVA release 132 as a reference database [53].

### 16S rRNA gene fragment-based microbial community analysis and statistics

After building the OTU table in QIIME, the table was imported into R, and only OTUs with a maximum relative abundance >0.01% in at least one sample were kept for further analyses. The OTU filtering was performed with the filter.OTU function of the RAM R package [54]. To determine if OTUs were consistently found among the three replicates of a sampling collection, we defined the OTUs that were detected in at least 2 of 3 replicates as “reproducibly occurring” OTUs (rOTUs) [11]. Therefore, the RAM::core.OTU function was used.

Barplots of the top ten taxa at five taxonomic ranks of seed potato generations were plotted to show the relative abundance with the RAM::group.top.number function.

The alpha diversity values were calculated with the rtk R package after multiple rarefactions and averaging the results of 9999 iterations [55]. The observed rOTUs were counted, and the diversity within each individual sample was estimated using Simpson's diversity index. Richness and diversity values were compared between the time points, cultivar or tuber parts by means of permutation ANOVA (i.e., perm.anova) and permutation pairwise comparison (i.e., pairwise.perm.t.test) functions in the RVAideMemoire R package [56]. The resulting P-values were adjusted by false discovery rate (FDR). Richness and diversity value boxplots were visualized with the RAM::group.diversity function. For identification of the core microbiota, the core_members function was used to calculate core OTUs with a prevalence of 99% and a detection threshold of 1 with the microbiome R package [57]. OTUs were visualized with the online tool jvenn [58].

For beta diversity analysis purposes, a normalization method based on cumulative sum scaling (CSS) [59] was used to remove biases in the count data with the metagenomeSeq Bioconductor package [60]. The differences between bacterial communities were investigated using the Bray–Curtis dissimilarity distance and the ordination methods applied to the same distance matrices. Multivariate analysis of community structure and diversity was performed as a constrained multidimensional scaling using constrained analysis of principle coordinates. All ordination analyses were computed, and CAP was plotted in the phyloseq R package [61]. The significance of the time point, cultivar or tuber parts grouping factor that was used as a constraint in the CAP was assessed via the permutation test in the vegan R package [62]. A hierarchical clustering using the Bray–Curtis dissimilarity distance was performed with the function RAM::data.clust.

Effects of each factor on the community composition (beta diversity) were tested by permutational multivariate analysis of variance (PERMANOVA). Therefore, the null hypothesis of no differences between a priori defined groups was investigated recurring to the PERMANOVA with the function vegan::adonis to the Bray–Curtis dissimilarity distances. Pairwise comparisons among levels of time points, cultivars and tuber parts were conducted using the package RVAideMemoire::pairwise.perm.manova. P values were adjusted by FDR. The applicability of the PERMANOVA was checked with the analysis of multivariate homogeneity of group dispersions as implemented in the vegan::betadisper function. Because multivariate homogeneity of group dispersions was not given in every grouping factor, a generalized linear model for multivariate abundance data was applied using the manyglm function of the mvabund R package [63] to confirm the results from PERMANOVA. An analysis of similarities was performed to test the effect of the grouping factor on the variables. Therefore, the vegan::anosim function was used.

Identification of rOTUs that were significantly enriched in the different potato varieties at different time points was carried out by extracting the OTUs after using the mvbund::manyglm function. Differentially abundant OTUs were visualized with the function RAM::group.abundance.meta.

### Nucleotide sequence accession numbers

Sequence data are available in the NCBI SRA database under the accession number SUB5027957 and the BioProject number PRJNA513967.

## Results and discussion

### Sequencing statistic

The sequencing of the V5-V7 region of the 16S rRNA gene from potato tuber samples yielded 9,631,766 high-quality merged reads, corresponding to an average of 41,877 reads per sample with an average read length of 360 bp. One sample (a tuber of variety Lady Claire grown in potting soil, first generation tuber) with less than 2,000 reads was excluded from further analysis. OTU clustering (97% similarity) of sequencing reads resulted in a total of 17,172 OTUs from 230 samples.

### Taxonomic composition of bacterial assemblages in seed potatoes

To obtain an overall idea of the bacterial assemblages associated with potato tubers and their variability across different potato varieties, we analyzed the taxonomy of 16S rRNA gene amplicons obtained from seed potato tubers (T0) of seven different varieties (Lady Claire, Hermes, Agria, Fontane, Ditta, Agata and Fabiola) and plotted the relative abundance of the top ten taxa (S2 Fig). At the phylum level, the potato tuber bacteria belonged predominantly to *Proteobacteria*, *Actinobacteria*, *Firmicutes* and *Bacteroidetes*, i.e., those taxa that usually dominate the bacterial community in plants [64]. On the class level, the most prominent taxon was *Actinobacteria*, which represents approximately 22% of the total bacterial community, followed by *Alphaproteobacteria* (approximately 16%), *Bacilli* (approximately 11%), *Betaproteobacteria* (approximately 10%) and *Gammaproteobacteria* (approximately 7%). This finding is in agreement with previous studies on the bacterial community associated with potatoes. Kõiv et al. (2015) compared the bacterial microbiota in potato tubers infected with *Pectobacterium atrospeticum* and uninfected tubers and found *Actinobacteria* to be dominant in the community followed by *Betaproteobacteria* and *Bacilli* [17]. Additionally, *Actinobacteria*, *Alphaproteobacteria* and *Gammaproteobacteria* have been described as consistently present in different potato cultivars [65].

### Impact of the soil microbiome on tuber-associated bacterial communities

**Fig 1:**
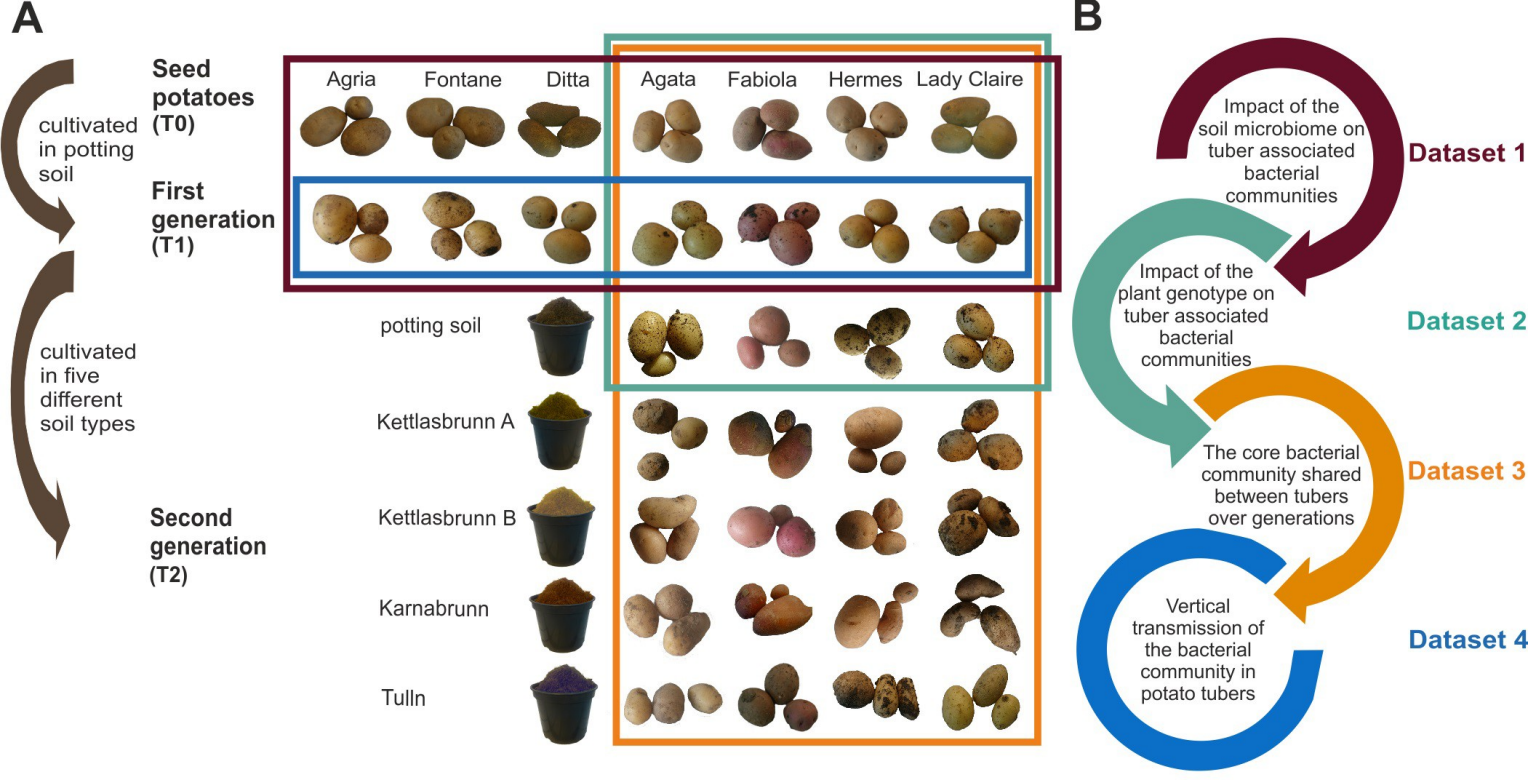
Overview of potato cultivars and potato tuber generations investigated in this study. Initially, seed potatoes of seven potato cultivars (Agata, Agria, Ditta, Fabiola, Fontane, Lady Claire and Hermes) were used for 16S rRNA gene amplicon sequencing (T0) and were grown in parallel in commercial potting soil. At maturity, tubers were harvested and used for 16S rRNA gene amplicon sequencing (T1). Tubers of four varieties (Agata, Fabiola, Hermes and Lady Claire) were planted in pots with five different soil types (commercial potting soil and four different farmland soils). Again, tubers were harvested at maturity and used for bacterial community sequencing (T2). The 16S rRNA gene amplicon sequencing data of different potato tuber generations and potato cultivars were combined to different datasets depending on the research question (datasets 1–4), as indicated by differently colored boxes.

The first step in our analysis was to compare the 16S rRNA gene amplicon sequencing data from seven different varieties (Lady Claire, Hermes, Agria, Fontane, Ditta, Agata and Fabiola) of seed potatoes (T0) with that from the tubers of these varieties grown in commercial potting soil in the greenhouse (first generation, T1) (dataset 1, Fig 1). This allowed us to test the hypothesis that the soil microbiota is the main reservoir for bacteria-colonizing potato tubers. If this hypothesis is true, the bacterial communities of the first generation tubers would be more similar to each other than to those of the seed potatoes, which came from different field sites.

Prior to statistical analysis, tuber parts were pooled to obtain insight into the whole tuber microbiome. After filtering for OTUs with at least 0.01% relative abundance and for OTUs that were present in at least two of three replicates, 728 rOTUs were obtained. Read numbers were rarefied to 19,751 reads in each sample. Permutation ANOVA of the rOTU richness revealed significant differences between the bacterial communities in tubers belonging to different potato cultivars (F-value = 19.836, p = 0.001*** for observed species) but not between both tuber generations. (S4 Table). In contrast to that, permutation ANOVA of Simspon’s Index showed no significant differences. Overall, alpha diversity analysis revealed that tubers of different potato varieties differ in bacterial richness and evenness, but these parameters were not affected by cultivation of the different potato varieties in the same soil type (commercial potting soil) (S3A Fig).

**Fig 2:**
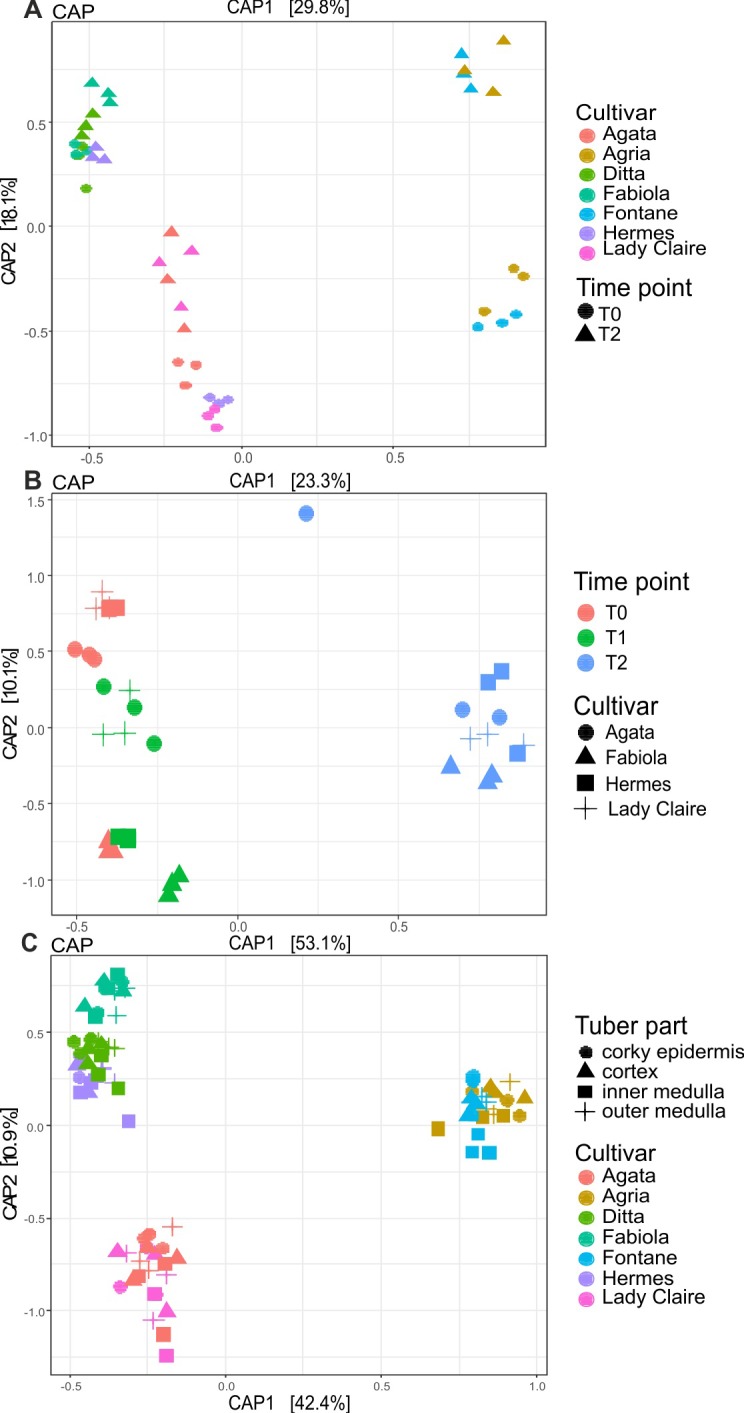
Constrained analysis of principal coordinates (CAP) of Bray–Curtis dissimilarities. CAP based on the V5–V7 regions of the 16S rRNA gene investigated for datasets 1, 2 and 4. **A.** Dataset 1, **B.** dataset 2, **C.** dataset 4. An overview of potato cultivars and potato tuber generations combined with different datasets is shown in Fig 1.

In contrast, the bacterial community composition (beta diversity) was affected by both the potato genotype and cultivation in potting soil (Fig 2A). The samples grouped according to the potato variety and essentially formed three clusters including i) tubers of Agria and Fontane, ii) Ditta and Fabiola, and iii) Agata, Hermes and Lady Claire. Furthermore, seed potatoes were separated from second generation tubers. The analysis of similarities (ANOSIM) (9999 permutations) confirmed the separation of the different cultivars in the CAP (P = 0.0001 and R value = 0.5467) (S5 Table). The permutation CAP test on the Bray-Curtis dissimilarity matrix exposed a clear discrimination between both tuber generations (P = 0.001***) and the different potato cultivars (P = 0.001***). This was confirmed by PERMANOVA on Bray-Curtis dissimilarity distance (between both tuber generation pseudo-F = 58.342, P = 0.0001*** and cultivar overall pseudo-F = 67.721, P = 0.0001***) and by the generalized linear models for multivariate abundance data on the Bray-Curtis dissimilarity matrix (999 permutations) (between both tuber P = 0.001*** and cultivar overall P = 0.001***) (S5 Table). Pairwise comparisons using PERMANOVA on the Bray-Curtis dissimilarity matrix showed that the bacterial community of tubers was significantly different in the seed potatoes and the first generation (P = 0.001) (S5 Table). In summary, beta diversity analysis revealed that cultivation in potting soil clearly affected the bacterial community composition in potato tubers. However, although grown in the same soil, tubers of different potato varieties still hosted distinct bacterial assemblages.

This shows that different plant genotypes, to a certain extent, select for different microbiota-colonizing potato tubers, e.g., by differences in root exudates, as is well known for the rhizosphere and roots [66–70]. On the other hand, the results also indicate that a part of the tuber bacterial community is independent of the soil and seems to be transmitted from one tuber generation to the next. This prompted us to test how repeated cultivation in potting soil would affect the bacterial assemblages associated with tubers. We assumed that if different plant genotypes specifically recruit bacteria from the soil, each cultivar would host similar bacterial communities in tubers of generations 1 and 2 (both grown in potting soil) and that the bacterial assemblage in second generation tubers would be significantly different between cultivars.

### Impact of the plant genotype on tuber-associated bacterial communities

To address this question, we compared the bacterial communities associated with tubers of the varieties Lady Claire, Hermes, Agata and Fabiola of three different generations: seed potato (T0), tubers grown in commercial potting soil (first generation tubers, T1) and tubers from plants emerging from T1 tubers and grown in commercial potting soil (second generation tubers, T2) (dataset 2, Fig 1). We again pooled the parts of the tubers to obtain an insight of the whole tuber microbiome and filtered for OTUs with at least 0.01% relative abundance and the “reproducibly occurring” OTUs. After both filtering steps, 894 rOTUs were obtained. Read numbers were rarefied to 12,410 reads in each sample. Permutation ANOVA of the rOTU richness and Simpson’s index (9999 permutations) did not show significant differences in the alpha diversity of bacterial communities between the different cultivars and time points. Pairwise comparison using permutation t-tests of the rOTU richness also did not reveal differences between the potato tuber cultivars and the generations (S4 Table), although bacterial richness slightly decreased with each potato generation (S3B Fig).

**Fig 3:**
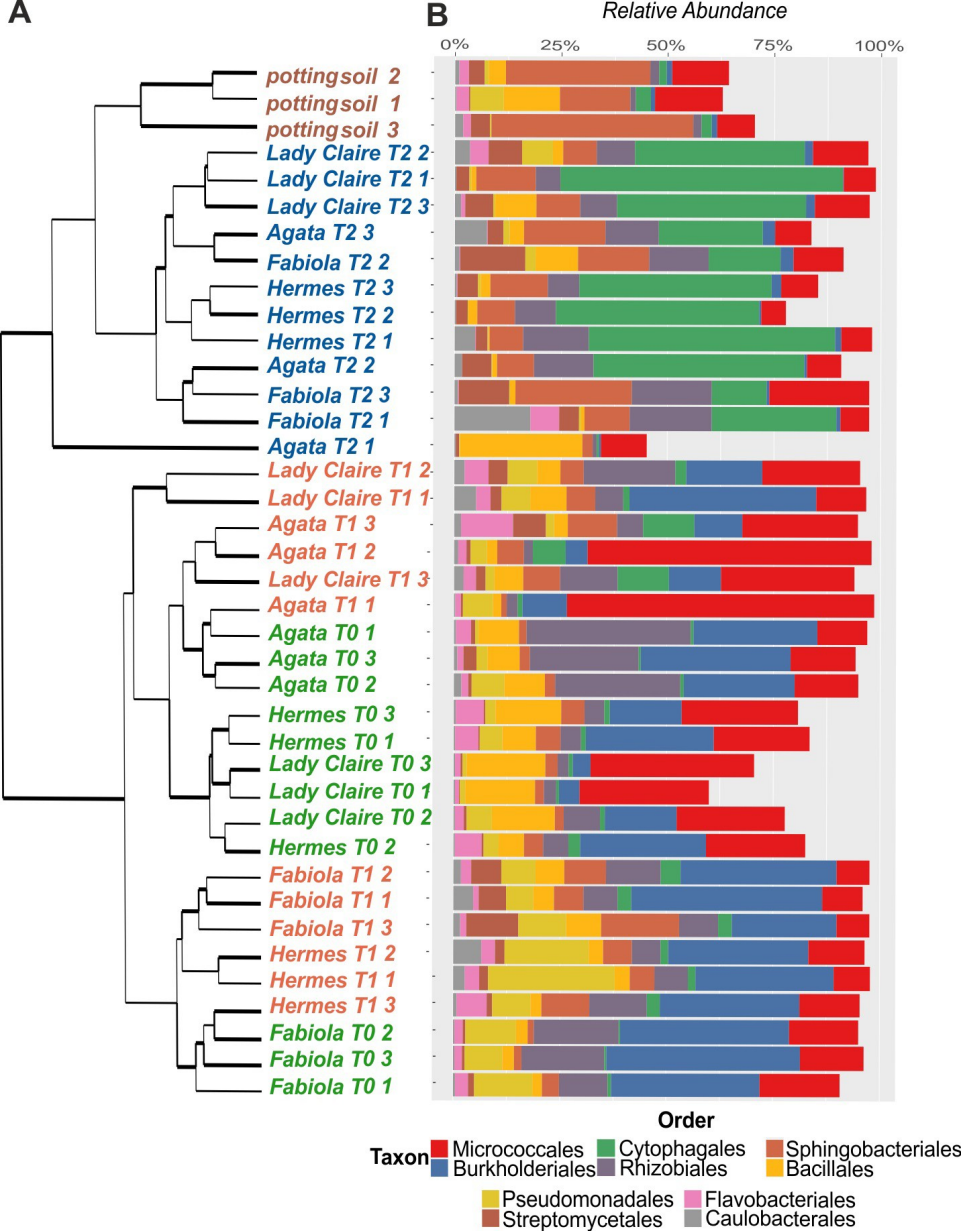
Hierarchical clustering of the Bray-Curtis dissimilarity matrix and differentially abundant taxa on the order level. **A.** Hierarchical clustering of the Bray-Curtis dissimilarity matrix on order level and **B**. differentially abundant taxonomic groups on the order level of the bacteria community in tubers of four different potato cultivars (Agata, Fabiola, Hermes and Lady Claire) of three different generations (T0-T2) grown in commercial potting soil (dataset 2).

By including the data on the bacterial microbiota in potting soil and performing hierarchical clustering of the samples based on Bray-Curtis dissimilarity distance on class level, we found that the samples formed two clusters. Seed potato tubers and first generation tubers clustered together, while second generation tubers and potting soil clustered together. However, the tuber and the potting soil microbiomes were clearly separated (Fig 3A). The bacterial communities of the seed potatoes and the first generation tubers still clustered according to the cultivar, while those of the second generation tubers did not cluster according to the potato cultivar.

The CAP scaling based on Bray-Curtis dissimilarity distance of the bacterial assemblage of the three different generations confirmed this result and revealed that the composition of the bacterial community in the second generation tubers was clearly different from that in the seed and first generation tubers (Fig 2B). ANOSIM (9999 permutations) exposed a clear separation of the different time points in the CAP (P = 0.0001 and R value = 0.6821) (S5 Table).

The PERMANOVA on Bray-Curtis dissimilarity distance exposed that the differences in the bacterial community composition could be explained by the tuber generation (pseudo F-test = 22.117, P = 0.0001***) but not by the potato cultivar. The same results were shown by testing the generalized linear models for multivariate abundance data on the Bray-Curtis dissimilarity matrix (999 permutations) (tuber generation P = 0.001***) (S5 Table). Similarly, pairwise comparisons using PERMANOVA on Bray-Curtis dissimilarity distance revealed that the microbial communities of the tuber samples differed significantly between seed potatoes, first- and second generation tubers (T0 to T1 P = 0.003, T0 to T2 P = 0.00015) (S5 Table). In contrast, the potato tuber cultivar showed no significant differences when tested with each other (S5 Table).

To specify the differences in the bacterial community composition between the different tuber generations and potting soil, differentially abundant OTUs were extracted after calculation of the generalized linear models for multivariate abundance data (999 permutations) on Bray-Curtis dissimilarity distance (Fig 3B). Taxa explaining the differences between the bacterial communities of the second generation tubers and the other two tuber generations were very similar to those explaining the difference between the potting soil and the T0 and T1 tubers, i.e., in soil samples and T2 tubers, the relative abundance of *Streptomycetales* was higher compared to T0 and T1 tubers (S6 Table).

In summary, the results showed that the bacterial communities of potato tubers changed over generations and became more similar to the soil bacterial community, while the impact of the potato cultivar on the bacterial assemblage lost significance over time. This resembles the results of a study on the bacterial community in the rhizosphere of different potato cultivars grown in two different sites and in different years, showing that the effect of the field site overrides the effect of the genotype [23]. Additionally, it has been shown that the bacterial communities in roots of different potato cultivars were different, dependent on the soil in which they were grown [22].

### The core bacterial community shared between tubers of four potato cultivars and over generations

The next step in this study was to predict the number of rOTUs shared across tubers of the tested potato cultivars. We assumed that if the bacterial assemblage of the tuber was recruited from the soil more or less independently from the plant genotype, the number of rOTUs shared among the four potato cultivars would increase with each tuber generation. Furthermore, this analysis will identify rOTUs shared between tubers of different plant generations, i.e., those OTUs putatively vertically transferred from one plant generation to the next.

For this analysis, we included data from seed potato, first generation tubers and second generation tubers grown in potting soil as well as in four different farmland soils (dataset 3, Fig 1). Overall, 1361 OTUs were obtained after filtering for 0.01% relative abundance. To identify rOTUs shared between tubers of all potato cultivars in each tuber generation, “core rOTUs” were extracted from the dataset by filtering for rOTUs present in at least two of three replicates per sample type and in all four potato cultivars. This resulted in a total of 1310 core rOTUs. Afterwards, core rOTUs with a 99% prevalence in each tuber generation were calculated.

**Fig 4:**
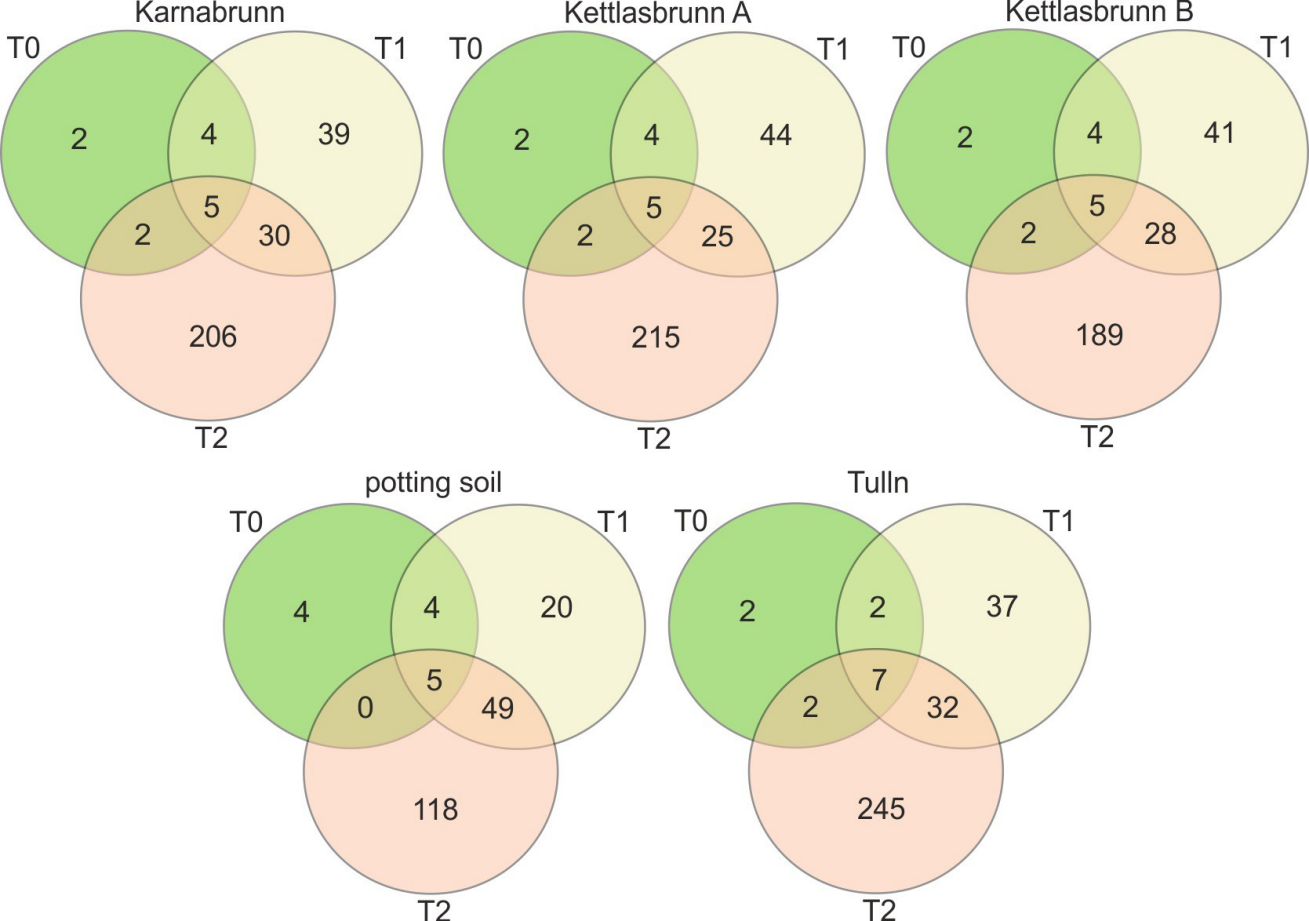
Numbers of core rOTUs in sequencing data of the bacterial communities of three potato tuber generations (dataset 3) shown in a Venn diagram. Here, samples were split according to the different soil types used for the cultivation of the second generation tubers (T2). For filtering, core OTUs with a prevalence of 99% were selected.

The Venn diagrams of core rOTUs illustrate the steady increase of core rOTUs during cultivation in commercial potting soil and in different farmland soils (Fig 4). Seed potatoes of the four tested cultivars had 13 rOTUs in common. In first generation tubers, the number of OTUs reproducibly occurring in all cultivars increased to 78 and in second generation tubers, the number of core rOTUs increased to172 to 286, depending on the soil in which the tubers grew. In addition, nine core rOTUs were found in seed potatoes as well as in first generation tubers, and between 30 and 54 core rOTUs were present in first and second generation tubers. Furthermore, all three tuber generations shared five to seven core rOTUs. Four of them were also found in the second generation tubers independent of the soil they grew in. We defined this set of core OTUs common to all tuber generations in each potato variety and independent from the cultivation soil as the core bacterial community. Taxonomic analysis revealed that they represent members of the families *Bacillaceae* (OTU_1), *Comamonadaceae* (OTU_22), *Micrococcaceae* (OTU_8) and *Nocardioidaceae* (OTU_97) (S7 Table). The first three could be assigned at the genus level; OTU_1 belongs to the genus Bacillus, OTU_22 to Variovorax and OTU_8 to Pseudarthrobacter (S7 Table).

In summary, the results revealed that the number of rOTUs shared among tubers of the four cultivars increased from generation to generation, indicating that the bacteria colonizing tubers are recruited from the soil more or less independently from the plant genotype. We found parallels to these results in a study in which the microbiota of tomato seeds was not affected by the plant genotype [71]. Moreover, selected bacteria were inherited over generations. The heritability of a specific cohort of microorganisms among plant generations was recently reported for the clonal plant *Glechoma hederacea* [72] and plant reproductive organs [36, 71, 73, 74].

Given that the soil is the main reservoir for bacteria that colonize potato tubers, we wanted to know whether the microorganisms enter the tubers directly or indirectly, i.e., via the roots. We assumed that if the entry point was the tuber itself, the bacterial communities in the outer parts of the tubers would be more complex and more similar to those in the soil and less similar to those in the inner parts of the tubers.

### Vertical transmission of the bacterial community in potato tubers

For this analysis, we compared the bacterial communities in four different parts of tubers of seven different potato cultivars grown in potting soil (first generation tubers, T1) (Fig 1). After filtering for 0.01% relative abundance and extracting rOTUs, 815 rOTUs remained. Read numbers were rarefied to 2,847 reads in each sample.

OTU richness was slightly lower in the inner medulla, while Simpson’s index showed consistently high diversity in all tuber parts (S3C Fig).

Permutation ANOVA of the rOTU richness and Simpson’s index (9999 permutations) exposed significant differences between the potato tuber microbiome of the different cultivars (F-value = 9.7788, p = 0.0001*** for observed species and F-value = 10.448, p = 0.0001*** for Simpson’s diversity values) but not between tuber parts. (S4 Table) (S3C Fig). The CAP scaling of the bacterial communities showed a clustering of cultivars into different groups (Fig 2C), while the different tuber parts did not cluster. ANOSIM (9999 permutations) confirmed that there was no separation between the tuber parts present (P = 0.4222 and R value = -0.0001237) (S5 Table). PERMANOVA on Bray-Curtis dissimilarity distance revealed that the microbial communities of the different cultivars were significantly different from each other (pseudo F-test = 20.348, P = 0.00001***), but the different tuber compartments did not host significantly different communities. The same results were shown by testing the generalized linear models for multivariate abundance data on the Bray-Curtis dissimilarity matrix (999 permutations) (P = 0.001***) (S5 Table). Furthermore, pairwise comparisons using PERMANOVAs on Bray-Curtis dissimilarity distance showed that the microbial communities of the different tuber compartments did not change significantly from the corky epidermis to the inner medulla.

By extracting differently abundant OTUs of the different cultivars, it could be confirmed that the bacterial taxa in the different tuber parts were similar to each other but different compared to those found in the soil. In detail, the relative abundance of *Sphingobacteriales* was higher in soil samples than in tuber parts, while *Burkholderiales* were more prominent in tuber parts than in soil (S8 Table).

In summary, the results did not indicate a significant loss in complexity of the bacterial assemblage of potato tubers across the different parts. Furthermore, the communities in the different tuber parts did not differ significantly, while the soil bacterial community showed significant differences in the tuber microbiota composition. Based on these results, we assume that bacteria do not predominantly migrate into potato tubers via the surface (corky epidermis) but might colonize the tubers from the inside of plants. Roots exudate sugars, amino acids and organic acids, providing a rich nutrient source for microorganisms in the rhizosphere, favoring the migration of soil microbiota in the rhizosphere and roots [75]. In contrast, the tuber surface might provide limited nutrients as a result of cell decay or lesions only [76] and might therefore attract soil microbiota to a much smaller extent.

**Fig 5:**
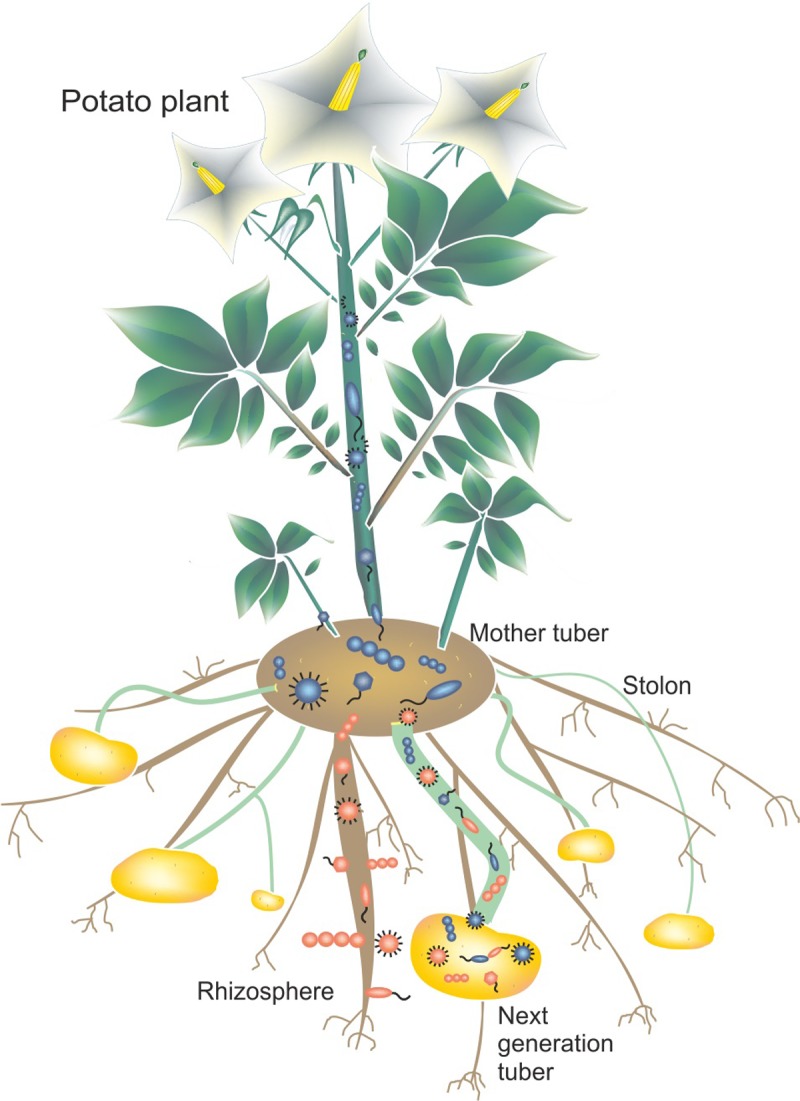
Graphical presentation of possible routes of colonization of potato tubers by bacteria. Microbiota colonizing the rhizosphere, entering the roots and colonizing the next tuber generation via the stolons, are visualized with a red color. Bacteria present in the mother tuber, passing through the stolons and migrating into the plant as well as into the next generation of tubers are shown in blue.

A possible scenario of potato tuber colonization (Fig 5) is that bacteria in the rhizosphere enter potato plant roots, pass through roots and reach the cortical cell layer or endodermis. Some bacteria may migrate via the xylem or intracellular spaces to the above ground tissues of the potato plants [77] as well as the stolon [78] and subsequently into the emerging tubers. In addition, bacteria colonizing mother tubers might migrate into the emerging roots and further to other above ground and below ground plant parts as described above, including the stolon and subsequently the next generation of tubers. Similarly, transmission of *Pectobacterium carotovorum* ssp. *atrosepticum* from one potato tuber generation to the next via stolon was reported elsewhere [78].

## Conclusions

In summary, the data of this study revealed the following:

- The soil is the main reservoir for bacteria that colonize potato tubers
- Bacteria are recruited from the soil more or less independent of the potato variety
- Bacteria might colonize the tubers predominantly from the inside of plants via the stolon
- The bacterial microbiota of potato tubers consists of bacteria transmitted from one tuber generation to the next and bacteria recruited from the soil colonize potato plants via the roots

## Supporting information

S1 Fig Overview of the tuber parts selected for bacterial community analysis. In detail, the corky epidermis characterizes the shell of the potato tuber followed by the cortex, which is defined by the tissue between the skin and corky epidermis. Inside the tubers, the outer and inner medulla represent two parts of the primary storage area for the potato tuber.
(TIF)

S2 Fig Bacterial community composition of seed potato tubers. In detail, the bacterial composition of the cultivars Agata, Agria, Ditta Fabiola, Fontane, Hermes and Lady Claire. The boxplots present the relative abundances of the top ten taxon groups at five taxonomic ranks. Values of the relative abundance are shown in S3 Table.
(TIF)

S3 Fig Alpha diversity as measured by bacterial richness and Simpson index. Values are visualized with boxplots to provide a comprehensive assessment of bacterial diversity for datasets 1, 2 and 4. **A.** Dataset 1, **B.** dataset 2, **C.** dataset 4. An overview of the potato cultivars and potato tuber generations that were combined with different datasets is shown in Fig 1.
(TIF)

S1 Table Primers used for the amplification of region V5-V7 of the 16S rRNA gene of the potato tuber bacterial communities. Sample-specific indices are shown in red.
(PDF)

S2 Table Primers used for the amplification of region V5-V7 of the 16S rRNA gene of the potato tuber bacterial communities. Sample-specific indices are shown in red.
(PDF)

S3 Table Taxonomic classification, relative abundance and relative abundance in % of the bacterial community in the seed potato tubers. Specifically, of the cultivars Agata, Agria, Ditta Fabiola, Fontane, Hermes and Lady Claire. The top ten taxa (visualized in S2 Fig) and not assigned taxa (NA) are shown at five different taxonomic ranks.
(PDF)

S4 Table Statistical analysis of alpha diversity values measured by bacterial richness and Simpson index. This was accomplished with permutation ANOVA and pairwise comparison permutation t-test between the following factors: time point, tuber parts and/or cultivar of dataset 1, 2 and 4.
(PDF)

S5 Table Statistical analysis of beta diversity. This was examined with PERMANOVA, permutation CAP test multivariate generalized linear model and ANOSIM based on the Bray-Curtis dissimilarity matrix of datasets 1, 2 and 4.
(PDF)

S6 Table Taxonomic classification of differentially abundant rOTUs. Differentially abundant rOTUs of the bacterial communities based on the sequencing data from all potato tuber generations (T0-T2) are shown. Tubers were grown in commercial potting soil (dataset 2).
(PDF)

S7 Table Taxonomic classification of shared and unique rOTUs. Shared and unique rOTUs of the bacterial communities based on the sequencing data from three potato tuber generations (dataset 3) are shown in Venn diagrams (Fig 4). Samples were split according to soil types used for the cultivation of second generation tubers (T2).
(PDF)

S8 Table Taxonomic classification of differentially abundant rOTUs. Differentially abundant rOTUs of the bacterial communities based on the sequencing data of potato tubers from the first generation (dataset 4) are shown.
(PDF)
