# Supplementary figures and images for: The bacterial community in potato is recruited from soil and partly inherited across generations
Source: PLoS One. 2019 Nov 8;14(11):e0223691. doi: 10.1371/journal.pone.0223691 (PMC6839881; doi:10.1371/journal.pone.0223691)

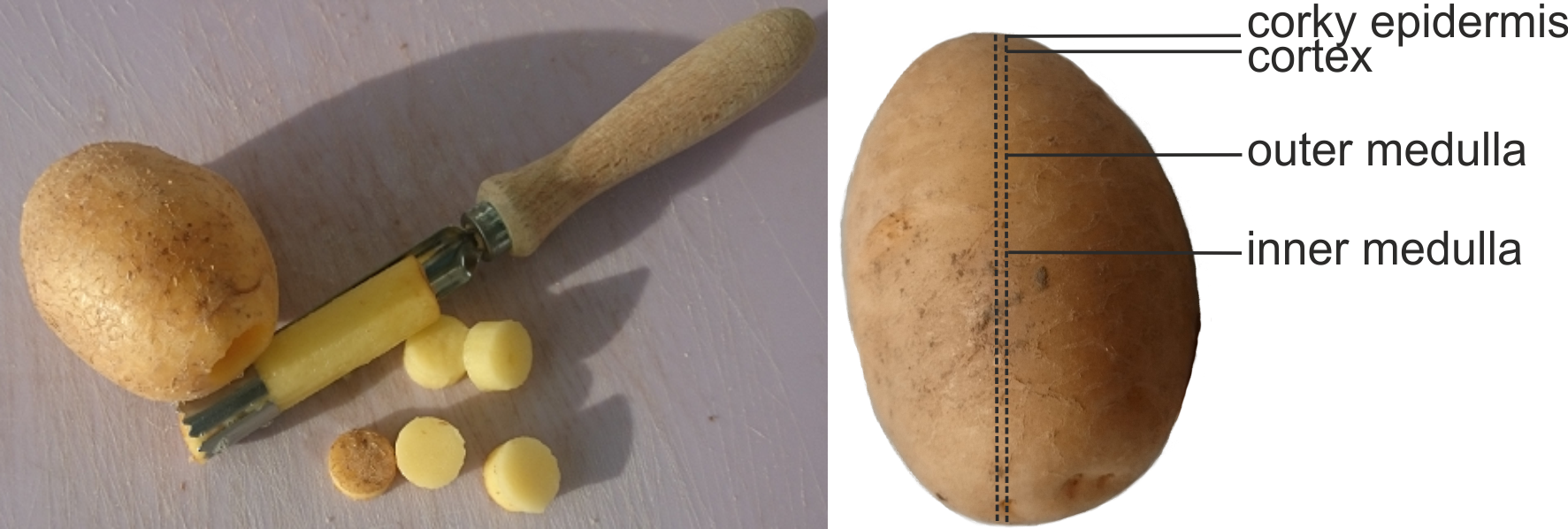

Supplement: S1 Fig — In detail, the corky epidermis characterizes the shell of the potato tuber followed by the cortex, which is defined by the tissue between the skin and corky epidermis. Inside the tubers, the outer and inner medulla represent two parts of the primary storage area for the potato tuber. (TIF) [file pone.0223691.s001.tif]

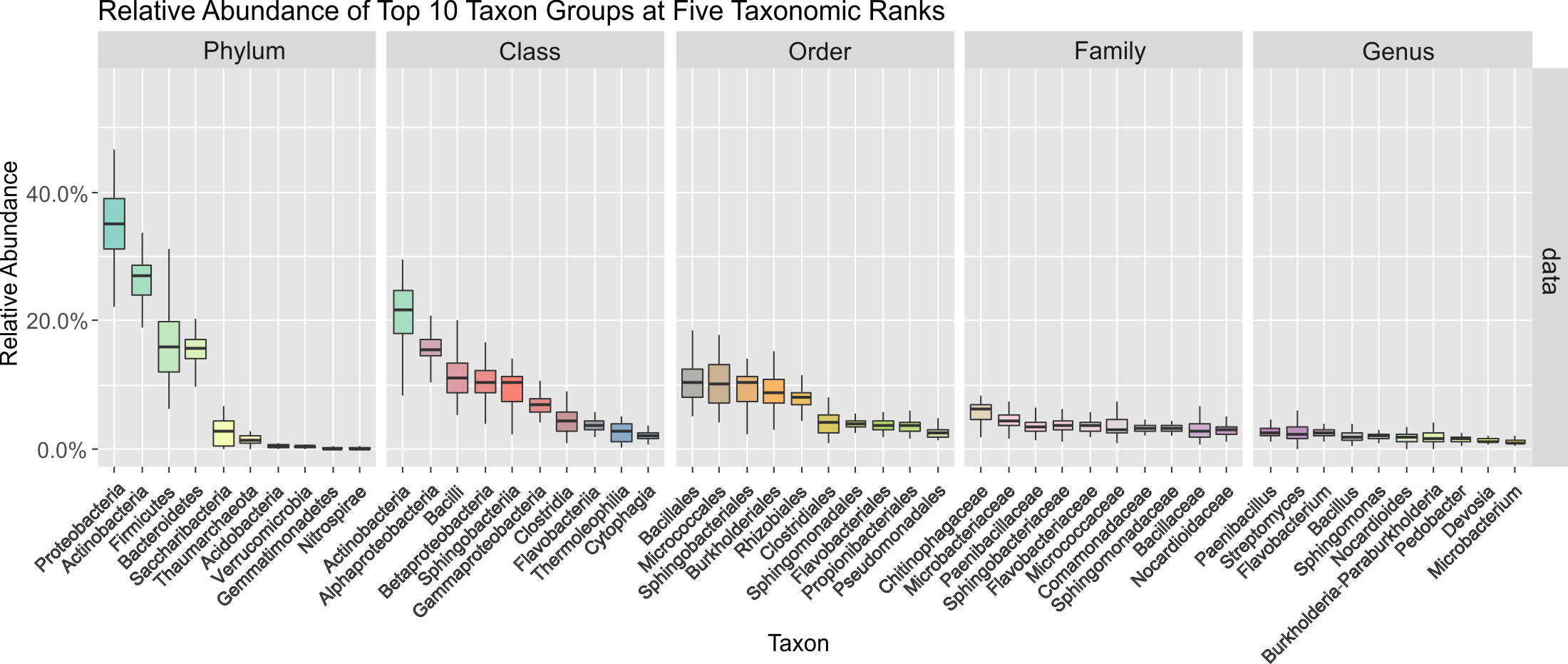

Supplement: S2 Fig — In detail, the bacterial composition of the cultivars Agata, Agria, Ditta Fabiola, Fontane, Hermes and Lady Claire. The boxplots present the relative abundances of the top ten taxon groups at five taxonomic ranks. Values of the relative abundance are shown in S3 Table. (TIF) [file pone.0223691.s002.tif]

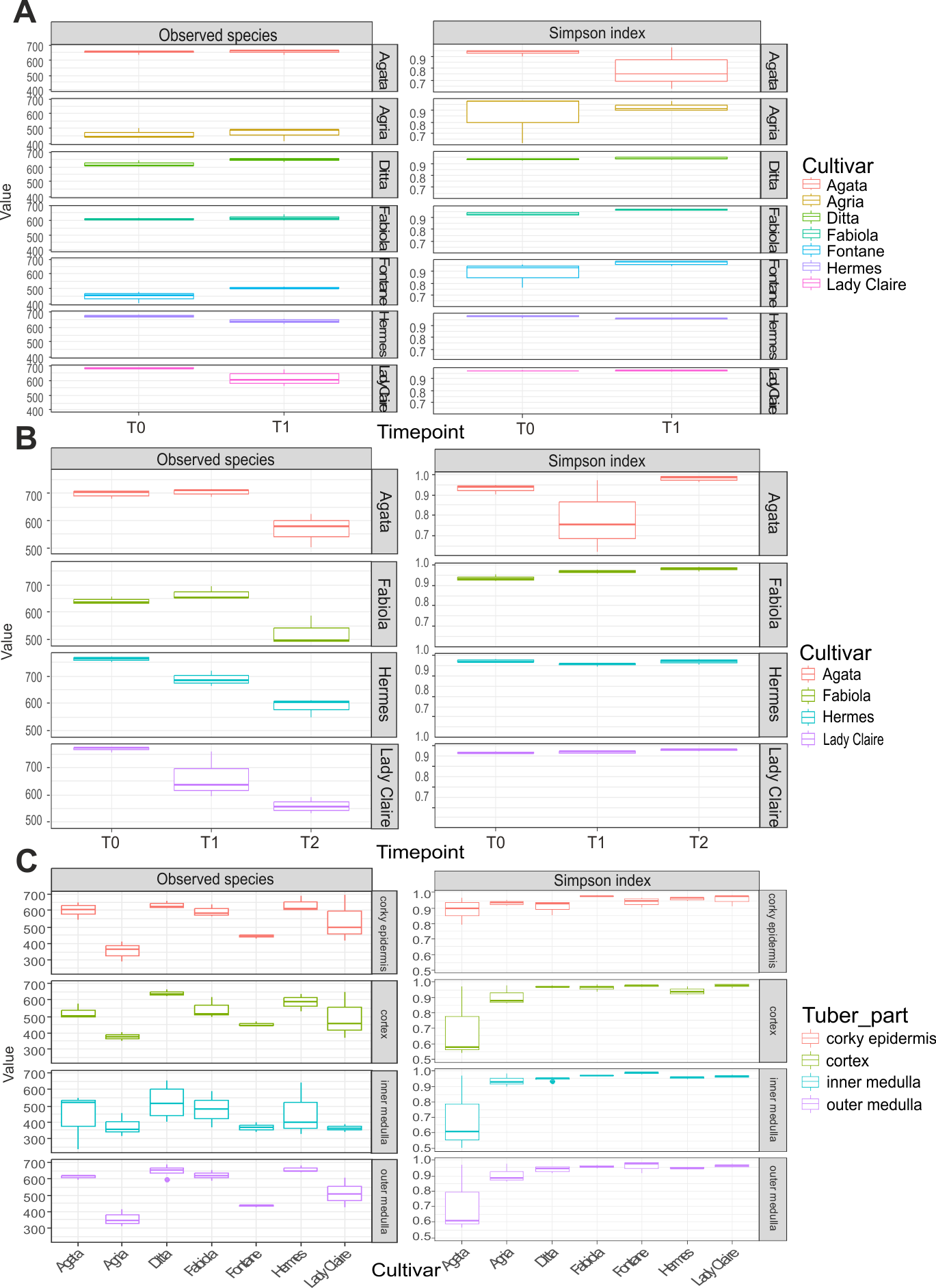

Supplement: S3 Fig — Values are visualized with boxplots to provide a comprehensive assessment of bacterial diversity for datasets 1, 2 and 4. A. Dataset 1, B. dataset 2, C. dataset 4. An overview of the potato cultivars and potato tuber generations that were combined with different datasets is shown in Fig 1. (TIF) [file pone.0223691.s003.tif]
