# Supplementary material for: The bacterial community in potato is recruited from soil and partly inherited across generations
Source: PLoS One. 2019 Nov 8;14(11):e0223691. doi: 10.1371/journal.pone.0223691 (PMC6839881; doi:10.1371/journal.pone.0223691)
Supplement: S1 Table — Sample-specific indices are shown in red. (PDF) [file pone.0223691.s004.pdf]

Table S1: **Primers used for the amplification of region V5-V7 of the 16S rRNA gene of potato tuber bacterial communities**. Sample-specific indices are shown in red.

Primer name	Oligo Sequence (5´- 3´)
	16S rRNA PCR round 1
799f	MGGATTAGATACCCKGGT
1392r	ACGGGCGGTGTGTRC
	16S rRNA PCR round 2
799f_mod6_1	CTACGGMGGATTAGATACCCKGGT
799f_mod6_2	CGTGCCMGGATTAGATACCCKGGT
799f_mod6_3	CAAGTGMGGATTAGATACCCKGGT
799f_mod6_4	GCGCCAMGGATTAGATACCCKGGT
799f_mod6_5	GGCAAGMGGATTAGATACCCKGGT
799f_mod6_6	TCTCAGMGGATTAGATACCCKGGT
799f_mod6_7	CTGGTTMGGATTAGATACCCKGGT
799f_mod6_8	CCTGAGMGGATTAGATACCCKGGT
799f_mod6_9	TTAGCTMGGATTAGATACCCKGGT
799f_mod6_10	TAGGCAMGGATTAGATACCCKGGT
799f_mod6_11	GAACATMGGATTAGATACCCKGGT
799f_mod6_12	TCGAACMGGATTAGATACCCKGGT
1175r_1	CTACGGAGCTCRTCCCCDCCTTCCT
1175r_2	CGTGCCAGCTCRTCCCCDCCTTCCT
1175r_3	CAAGTGAGCTCRTCCCCDCCTTCCT
1175r_4	GCGCCAAGCTCRTCCCCDCCTTCCT
1175r_5	GGCAAGAGCTCRTCCCCDCCTTCCT
1175r_6	TCTCAGAGCTCRTCCCCDCCTTCCT
1175r_7	CTGGTTAGCTCRTCCCCDCCTTCCT
1175r_8	CCTGAGAGCTCRTCCCCDCCTTCCT
