# Supplementary material for: The bacterial community in potato is recruited from soil and partly inherited across generations
Source: PLoS One. 2019 Nov 8;14(11):e0223691. doi: 10.1371/journal.pone.0223691 (PMC6839881; doi:10.1371/journal.pone.0223691)
Supplement: S2 Table — Sample-specific indices are shown in red. (PDF) [file pone.0223691.s005.pdf]

Table S2: **Primers used for the amplification of region V5-V7 of the 16S rRNA gene of potato tuber bacterial communities.** Sample-specific indices are shown in red.

Primer name		Oligo Sequence (5´- 3´)	
16S rRNA PCR round 1			
799f		AACMGGATTAGATACCCKG	
1175r		ACGTCRTCCCCDCCTTCCT	
16S rRNA PCR round 2			
Primer name	Oligo Sequence (5´- 3´)	Primer name	Oligo Sequence (5´- 3´)
799F_1	ACAACCAGTTAACMGGATTAGATACCCKG	1175R_1	ACAACCAGTTACGTCRTCCCCDCCTTCCT
799F_2	NAACAGACCTTAACMGGATTAGATACCCKG	1175R_2	NAACAGACCTTACGTCRTCCCCDCCTTCCT
799F_3	NNACAAGGTCTTAACMGGATTAGATACCCKG	1175R_3	NNACAAGGTCTTACGTCRTCCCCDCCTTCCT
799F_4	NNNAAGTCTTCGTAACMGGATTAGATACCCKG	1175R_4	NNNAAGTCTTCGTACGTCRTCCCCDCCTTCCT
799F_5	ACATGAGGTTAACMGGATTAGATACCCKG	1175R_5	ACATGAGGTTACGTCRTCCCCDCCTTCCT
799F_6	NAAGCTCACTTAACMGGATTAGATACCCKG	1175R_6	NAAGCTCACTTACGTCRTCCCCDCCTTCCT
799F_7	NNACGATACGTTAACMGGATTAGATACCCKG	1175R_7	NNACGATACGTTACGTCRTCCCCDCCTTCCT
799F_8	NNNAATGCGCTATAACMGGATTAGATACCCKG	1175R_8	NNNAATGCGCTATACGTCRTCCCCDCCTTCCT
799F_9	ACCTCATCTTAACMGGATTAGATACCCKG	1175R_9	ACCTCATCTTACGTCRTCCCCDCCTTCCT
799F_10	NAACAGCTCATAACMGGATTAGATACCCKG	1175R_10	NAACAGCTCATACGTCRTCCCCDCCTTCCT
799F_11	NNACTGTTGACTAACMGGATTAGATACCCKG	1175R_11	NNACTGTTGACTACGTCRTCCCCDCCTTCCT
799F_12	NNNAGAGTTGCTTAACMGGATTAGATACCCKG	1175R_12	NNNAGAGTTGCTTACGTCRTCCCCDCCTTCCT
799F_13	ACCTTGACATAACMGGATTAGATACCCKG	1175R_13	ACCTTGACATACGTCRTCCCCDCCTTCCT
799F_14	NACAACTGTGTAACMGGATTAGATACCCKG	1175R_14	NACAACTGTGTACGTCRTCCCCDCCTTCCT
799F_15	NNACTTAGCACTAACMGGATTAGATACCCKG	1175R_15	NNACTTAGCACTACGTCRTCCCCDCCTTCCT
799F_16	NNNAATACGACCTAACMGGATTAGATACCCKG	1175R_16	NNNAATACGACCTACGTCRTCCCCDCCTTCCT
799F_17	ACGATCGTATAACMGGATTAGATACCCKG	1175R_17	ACGATCGTATACGTCRTCCCCDCCTTCCT
799F_18	NACCAATCAGTAACMGGATTAGATACCCKG	1175R_18	NACCAATCAGTACGTCRTCCCCDCCTTCCT
799F_19	NNAGGACTTGTTAACMGGATTAGATACCCKG	1175R_19	NNAGGACTTGTTACGTCRTCCCCDCCTTCCT
799F_20	NNNAGCTGAATCTAACMGGATTAGATACCCKG	1175R_20	NNNAGCTGAATCTACGTCRTCCCCDCCTTCCT
799F_21	ACTCACTGTTAACMGGATTAGATACCCKG	1175R_21	ACTCACTGTTACGTCRTCCCCDCCTTCCT
799F_22	NAGAGCAATGTAACMGGATTAGATACCCKG	1175R_22	NAGAGCAATGTACGTCRTCCCCDCCTTCCT
799F_23	NNAGTCATCCTTAACMGGATTAGATACCCKG	1175R_23	NNAGTCATCCTTACGTCRTCCCCDCCTTCCT
799F_24	NNNAGTAGCCTATAACMGGATTAGATACCCKG	1175R_24	NNNAGTAGCCTATACGTCRTCCCCDCCTTCCT
799F_25	AGATAGCGATAACMGGATTAGATACCCKG	1175R_25	AGATAGCGATACGTCRTCCCCDCCTTCCT
799F_26	NAACGGAACATAACMGGATTAGATACCCKG	1175R_26	NAACGGAACATACGTCRTCCCCDCCTTCCT
799F_27	NNATATAGCCGTAACMGGATTAGATACCCKG	1175R_27	NNATATAGCCGTACGTCRTCCCCDCCTTCCT
799F_28	NNNAGTGAACTCTAACMGGATTAGATACCCKG	1175R_28	NNNAGTGAACTCTACGTCRTCCCCDCCTTCCT
799F_29	ATACGGACTTAACMGGATTAGATACCCKG	1175R_29	ATACGGACTTACGTCRTCCCCDCCTTCCT
799F_30	NAGCTCCTTATAACMGGATTAGATACCCKG	1175R_30	NAGCTCCTTATACGTCRTCCCCDCCTTCCT
799F_31	NNATGCGCATATAACMGGATTAGATACCCKG	1175R_31	NNATGCGCATATACGTCRTCCCCDCCTTCCT
799F_32	NNNAGTGCTTCATAACMGGATTAGATACCCKG	1175R_32	NNNAGTGCTTCATACGTCRTCCCCDCCTTCCT
799F_33	ATCACCATGTAACMGGATTAGATACCCKG	1175R_33	ATCACCATGTACGTCRTCCCCDCCTTCCT
799F_34	NAGCTTCAGTTAACMGGATTAGATACCCKG	1175R_34	NAGCTTCAGTTACGTCRTCCCCDCCTTCCT
799F_35	NNATGTACTGGTAACMGGATTAGATACCCKG	1175R_35	NNATGTACTGGTACGTCRTCCCCDCCTTCCT
799F_36	NNNATCGGTAGTTAACMGGATTAGATACCCKG	1175R_36	NNNATCGGTAGTTACGTCRTCCCCDCCTTCCT
799F_37	ATCGAACCTTAACMGGATTAGATACCCKG	1175R_37	ATCGAACCTTACGTCRTCCCCDCCTTCCT
799F_38	NAGGTACAACTAACMGGATTAGATACCCKG	1175R_38	NAGGTACAACTACGTCRTCCCCDCCTTCCT

799F_39	NNATTGGAGTGTAACMGGATTAGATACCCKG	1175R_39	NNATTGGAGTGTACGTCRTCCCCDCCTTCCT
799F_40	NNNAGACATTCCTAACMGGATTAGATACCCKG	1175R_40	NNNAGACATTCCTACGTCRTCCCCDCCTTCCT
799F_41	ATGCAACACTAACMGGATTAGATACCCKG	1175R_41	ATGCAACACTACGTCRTCCCCDCCTTCCT
799F_42	NAGGTGTGTTTAACMGGATTAGATACCCKG	1175R_42	NAGGTGTGTTTACGTCRTCCCCDCCTTCCT
799F_43	ATGGTAACGTAACMGGATTAGATACCCKG	1175R_43	ATGGTAACGTACGTCRTCCCCDCCTTCCT
799F_44	NAGTCGATACTAACMGGATTAGATACCCKG	1175R_44	NAGTCGATACTACGTCRTCCCCDCCTTCCT
799F_45	ATTCACCTGTAACMGGATTAGATACCCKG	1175R_45	ATTCACCTGTACGTCRTCCCCDCCTTCCT
799F_46	NAGTTGAGCATAACMGGATTAGATACCCKG	1175R_46	NAGTTGAGCATACGTCRTCCCCDCCTTCCT
799F_47	ATTGACACCTAACMGGATTAGATACCCKG	1175R_47	ATTGACACCTACGTCRTCCCCDCCTTCCT
799F_48	NATACGTTGCTAACMGGATTAGATACCCKG	1175R_48	NATACGTTGCTACGTCRTCCCCDCCTTCCT
