# Supplementary material for: The bacterial community in potato is recruited from soil and partly inherited across generations
Source: PLoS One. 2019 Nov 8;14(11):e0223691. doi: 10.1371/journal.pone.0223691 (PMC6839881; doi:10.1371/journal.pone.0223691)
Supplement: S3 Table — Specifically, of the cultivars Agata, Agria, Ditta Fabiola, Fontane, Hermes and Lady Claire. The top ten taxa (visualized in S2 Fig) and not assigned taxa (NA) are shown at five different taxonomic ranks. (PDF) [file pone.0223691.s006.pdf]

Table S3: **Taxonomic classification, relative abundance and relative abundance in % of the bacterial community in seed potato tuber**. Specifically of the cultivars Agata, Agria, Ditta Fabiola, Fontane, Hermes and Lady Claire. Top ten taxa (visualized in Fig S2) as well as not assigned taxa (NA) are shown at five different taxonomic ranks.

No.	Rank	Taxa	relative Abundance	relative Abundance [%]
1	**Phylum**	Proteobacteria	0.350098326	35.01
2		Actinobacteria	0.269964819	27.00
3		Firmicutes	0.159993912	16.00
4		Bacteroidetes	0.157415964	15.74
5		Saccharibacteria	0.028036592	2.80
6		Thaumarchaeota	0.014303903	1.43
7		Acidobacteria	0.004697668	0.47
8		Verrucomicrobia	0.003547061	0.35
9		Gemmatimonadetes	0.00088304	0.09
10		Nitrospirae	0.000739974	0.07
		**NA**	0.014173946	1.42
1	**Class**	Actinobacteria	0.216399586	21.64
2		Alphaproteobacteria	0.154511696	15.45
3		Bacilli	0.110979526	11.10
4		Betaproteobacteria	0.104341219	10.43
5		Sphingobacteriia	0.10298655	10.30
6		Gammaproteobacteria	0.06893436	6.89
7		Clostridia	0.043814955	4.38
8		Flavobacteriia	0.037805373	3.78
9		Thermoleophilia	0.028089634	2.81
10		Cytophagia	0.020895097	2.09
		**NA**	0.025087589	2.51
1	**Order**	Bacillales	0.10390029	10.39
2		Micrococcales	0.102427575	10.24
3		Sphingobacteriales	0.10298655	10.30
4		Burkholderiales	0.088372338	8.84
5		Rhizobiales	0.081478164	8.15
6		Clostridiales	0.0409513	4.10
7		Sphingomonadales	0.039480822	3.95
8		Flavobacteriales	0.037805373	3.78
9		Propionibacteriales	0.037099972	3.71
10		Pseudomonadales	0.026053483	2.61
		**NA**	0.089381842	8.94
1	**Family**	Chitinophagaceae	0.062245547	6.22
2		Microbacteriaceae	0.043692944	4.37
3		Paenibacillaceae	0.03375108	3.38
4		Sphingobacteriaceae	0.036878958	3.69
5		Flavobacteriaceae	0.036032873	3.60
6		Micrococcaceae	0.029748135	2.97
7		Comamonadaceae	0.03319294	3.32
8		Sphingomonadaceae	0.032036244	3.20
9		Bacillaceae	0.027853005	2.79
10		Nocardioidaceae	0.029855028	2.99
		**NA**	0.24012287	24.01
1	**Genus**	Paenibacillus	0.02621119	2.62
2		Streptomyces	0.023841281	2.38
3		Flavobacterium	0.025235658	2.52
4		Bacillus	0.018532786	1.85
5		Sphingomonas	0.02005923	2.01
6		Nocardioides	0.017357367	1.74
7		Burkholderia-Paraburkholderia	0.01532084	1.53
8		Pedobacter	0.015488306	1.55
9		Devosia	0.012613305	1.26
10		Microbacterium	0.010381169	1.04
		**NA**	0.445989873	44.60
