# Supplementary material for: The bacterial community in potato is recruited from soil and partly inherited across generations
Source: PLoS One. 2019 Nov 8;14(11):e0223691. doi: 10.1371/journal.pone.0223691 (PMC6839881; doi:10.1371/journal.pone.0223691)
Supplement: S4 Table — This was accomplished with permutation ANOVA and pairwise comparison permutation t-test between the following factors: time point, tuber parts and/or cultivar of dataset 1, 2 and 4. (PDF) [file pone.0223691.s007.pdf]

Table S4: Statistical analysis of alpha diversity values measured by bacterial richness and Simpson index. This was accomplished with permutation ANOVA and pairwise comparison permutation t-test between factors time point, tuber parts and/or cultivar of dataset 1, 2 and 4.

Dataset 1

observed species

Permutation Analysis of Variance Table
9999 permutations

	Sum Sq	Df	MeanSQ	F value	Pr(>F)	
Cultivar	126.622	6	21103.7	19.863	1.00E-03	***
Residuals	37.187	35	1062.5			

	Sum Sq	Df	MeanSQ	F value	Pr(>F)	
Timepoint	2509	1	2508.9	1	0.432	
Residuals	161.300	40	4032.5			

Signif. codes: 0 ‘***’ 0.001 ‘**’ 0.01 ‘*’ 0.05 ‘.’ 0.1 ‘ ’ 1

Pairwise comparisons using permutation t tests
9999 permutations

	Agata	Agria	Ditta	Fabiola	Fontane	Hermes
Agria	0.01	-	-	-	-	-
Ditta	0.01	0.019	-	-	-	-
Fabiola	0.039	0.039	0.65	-	-	-
Fontane	0.01	0.548	0.045	0.074	-	-
Hermes	0.278	0.01	0.01	0.028	0.017	-
Lady Claire	0.068	0.01	0.014	0.01	0.01	0.508

	T0
T1	0.41

P value adjustment method: fdr

Simpson index

Permutation Analysis of Variance Table
9999 permutations

	Sum Sq	Df	Mean	Sq	F value	Pr(>F)
Cultivar	0.060947	6	0.0101578	15.693	1.45E-01	
Residuals	0	35	0.0064729			

	Sum Sq	Df	MeanSQ	F value	Pr(>F)	
Timepoint	0	1	0.0002561	0.0357	0.828	
Residuals	0	40	0.0071811			

Signif. codes: 0 ‘***’ 0.001 ‘**’ 0.01 ‘*’ 0.05 ‘.’ 0.1 ‘ ’ 1

Pairwise comparisons using permutation t tests
9999 permutations

	Agata	Agria	Ditta	Fabiola	Fontane	Hermes
Agria	0.762	-	-	-	-	-
Ditta	0.364	0.615	-	-	-	-
Fabiola	0.364	0.549	0.593	-	-	-
Fontane	0.52	0.674	0.944	0.762	-	-
Hermes	0.168	0.364	0.14	0.52	0.52	-
Lady Claire	0.14	0.364	0.042	0.364	0.412	0.593

	T0
T1	0.78

P value adjustment method: fdr

Dataset 2

observed species

Permutation Analysis of Variance Table
9999 permutations

	Sum Sq	Df	MeanSq	F value	Pr(>F)	
Cultivar	14.636	4	0.36589	23.241	7.40E-02	.
Residuals	53.527	34	0.15743			

	Sum Sq	Df	MeanSq	F value	Pr(>F)	
Timepoint	0.8474	2	0.4237	25.554	0.0932	.
Residuals	59.689	36	0.1658			

Signif. codes: 0 '***' 0.001 '**' 0.01 '*' 0.05 '.' 0.1 ' ' 1

Pairwise comparisons using permutation t tests
9999 permutations

	Agata	Fabiola	Hermes	Lady_Claire
Fabiola	0.463	-	-	-
Hermes	0.324	0.122	-	-
Lady_Claire	0.206	0.098	0.994	-
Substrat	0.971	0.73	0.798	0.738

	T0	T1
T1	0.47	-
T2	0.12	0.13

Simpson index

Permutation Analysis of Variance Table
9999 permutations

	Sum Sq	Df	MeanSq	F value	Pr(>F)	
Cultivar	0.035007	4	0.0087519	22.208	7.43E-02	.
Residuals	0	34	0.0039409			

	Sum Sq	Df	MeanSq	F value	Pr(>F)
Timepoint	0	2	0.0069522	16.137	0.2111
Residuals	0	36	0.0043082		

Signif. codes: 0 '***' 0.001 '**' 0.01 '*' 0.05 '.' 0.1 ' ' 1

Pairwise comparisons using permutation t tests
9999 permutations

	Agata	Fabiola	Hermes	Lady_Claire
Fabiola	0.35	-	-	-
Hermes	0.35	0.67	-	-
Lady_Claire	0.35	0.35	0.35	-
Substrat	0.35	0.39	0.35	0.54

	T0	T1
T1	0.6052	-
T2	0.0066	0.0462

P value adjustment method: fdr

Dataset 4

observed species

Permutation Analysis of Variance Table
9999 permutations

	Sum Sq	Df	MeanSq	F value	Pr(>F)	
Cultivar	132672	7	18953.2	9,7788	1.00E-04	***
Residuals	151178	78	1938.2			

	Sum Sq	Df	MeanSq	F value	Pr(>F)	
Tuber_part	11319	4	2829.8	0.841	0.5039	
Residuals	272532	81	3364.6			

Signif. codes: 0 ‘***’ 0.001 ‘**’ 0.01 ‘*’ 0.05 ‘.’ 0.1 ‘ ’ 1

Pairwise comparisons using permutation t tests
9999 permutations

	Agata	Agria	Ditta	Fabiola	Fontane	Hermes
Agria	0.1206	-	-	-	-	-
Ditta	0.3149	0.0081	-	-	-	-
Fabiola	0.0028	0.0011	0.0019	-	-	-
Fontane	0.2581	0.0028	0.9074	0.0011	-	-
Hermes	0.133	0.0011	0.5257	0.0011	0.4185	-
Lady Claire	0.0185	0.0011	0.0616	0.7439	0.0351	0.0556

	corky epidermis	cortex	inner medulla	outer medulla
cortex	0.85	-	-	-
inner medulla	0.88	0.85	-	-
outer medulla	0.88	0.85	0.94	-

Simpson index

Permutation Analysis of Variance Table
9999 permutations

	Sum Sq	Df	MeanSq	F value	Pr(>F)	
Cultivar	0.4436	7	0	10,448	1.00E-04	***
Residuals	0.47308	78	0.006065			

	Sum Sq	Df	MeanSq	F value	Pr(>F)	
Tuber_part	0.01678	4	0.0041947	0.3776	0.8161	
Residuals	0.8999	81	0.0111099			

Signif. codes: 0 ‘***’ 0.001 ‘**’ 0.01 ‘*’ 0.05 ‘.’ 0.1 ‘ ’ 1

Pairwise comparisons using permutation t tests
9999 permutations

	Agata	Agria	Ditta	Fabiola	Fontane	Hermes
Agria	0.0218	-	-	-	-	-
Ditta	0.0176	0.3083	-	-	-	-
Fabiola	0.0056	0.0176	0.0176	-	-	-
Fontane	0.0176	0.0314	0.0915	0.8788	-	-
Hermes	0.0176	0.0702	0.3932	0.0423	0.2282	-
Lady Claire	0.0112	0.0218	0.0556	0.8788	0.9874	0.1117

	corky epidermis	cortex	inner medulla	outer medulla
cortex	0.8	-	-	-
inner medulla	0.8	0.92	-	-
outer medulla	0.75	0.92	0.92	-

P value adjustment method: fdr
