# Supplementary material for: The bacterial community in potato is recruited from soil and partly inherited across generations
Source: PLoS One. 2019 Nov 8;14(11):e0223691. doi: 10.1371/journal.pone.0223691 (PMC6839881; doi:10.1371/journal.pone.0223691)
Supplement: S5 Table — This was examined with PERMANOVA, permutation CAP test multivariate generalized linear model and ANOSIM based on the Bray-Curtis dissimilarity matrix of datasets 1, 2 and 4. (PDF) [file pone.0223691.s008.pdf]

Table S5: Statistical analysis of beta diversity. This was examined with PERMANOVA, permutation CAP test, multivariate generalized linear model and ANOSIM based on Bray Curtis dissimilarity matrix of dataset 1, 2 and 4.

Dataset 1

Permutation test for homogeneity of multivariate dispersions

Cultivar

	Df	Sum Sq	Mean Sq	F	N.Perm	Pr(>F)	
Groups	6	0.096319	0.0160531	18.966	999	0.001	***
Residuals	35	0	0.0008464				

Timepoint

	Df	Sum Sq	Mean Sq	F	N.Perm	Pr(>F)	
Groups	1	0.001232	0.0012322	0	999	0.655	.
Residuals	40	0	0.0057414				

Pairwise comparisons for homogeneity of multivariate dispersions

(Observed p-value below diagonal, permuted p-value above diagonal)

	Agata	Agria	Ditta	Fabiola	Fontane	Hermes	Lady Claire
Agata		1.00E+01	1.58E+03	2.57E+03	1.00E+01	3.90E+02	0.501
Agria	5.14E-01		1.00E+01	1.00E+01	4.43E+03	2.00E+01	0.011
Ditta	1.61E+03	5.94E-02		4.89E+03	1.00E+01	1.00E+01	0.105
Fabiola	2.41E+03	8.56E-03	5.19E+03		1.00E+01	1.00E+01	0.186
Fontane	1.38E+00	4.48E+03	2.40E-01	1.16E-01		3.00E+01	0.008
Hermes	4.11E+02	3.66E+00	1.90E+01	6.09E+00	9.52E+00		0.437
Lady Claire	5.12E+03	4.49E+01	1.19E+03	1.69E+03	3.64E+01	4.65E+03	

	T0	T2
T0		0.641
T2	0.64568	

PERMANOVA on a distance matrix

9999 permutations

	Df	SumsOfSqs	MeanSqs	F.Model	R2	Pr(>F)	
Cultivar	6	12.555	0	67.721	0.53724	1.00E-04	***
Residuals	35	10.815	0.030899	0.46276			
Total	41	23.370	100.000				

	Df	SumsOfSqs	MeanSqs	F.Model	R2	Pr(>F)	
Timepoint	1	0	0	58.342	0.12729	1.00E-04	***
Residuals	40	203.952	0.050988	0.87271			
Total	41	233.700	100.000				

Pairwise comparisons using PERMANOVAs on a distance matrix

9999 permutations

	Agata	Agria	Ditta	Fabiola	Fontane	Hermes
Agria	0.0039	-	-	-	-	-
Ditta	0.0039	0.0039	-	-	-	-
Fabiola	0.0039	0.0039	0.1098	-	-	-
Fontane	0.0039	0.5707	0.0039	0.0039	-	-
Hermes	0.0691	0.0039	0.0056	0.0039	0.0039	-
Lady Claire	0.1765	0.0039	0.0039	0.0039	0.0039	0.1765

	T0
T2	1.00E-04

P value adjustment method: fdr

permutation CAP test on Bray Curtis dissimilarity matrix

	Df	SumsOfSqs	F	Pr(>F)	
Timepoint	1	0	221.661	0.001	***
Cultivar	6	125.552	155.924	0.001	***
Timepoint:Cultivar	6	0	50.698	0.001	***
Residual	28		0.37577		

Signif. codes: 0 '***' 0.001 '**' 0.01 '*' 0.05 '.' 0.1 ' ' 1

multivariate GLM

	Res.	Df	Df.diff	Dev	Pr(>Dev)
(Intercept)	41				
Timepoint	40	1	3254	0.001	***

	Res.	Df	Df.diff	Dev	Pr(>Dev)
(Intercept)	41				
Cultivar	35	6	18287	0.001	***

Signif. codes: 0 '***' 0.001 '**' 0.01 '*' 0.05 '.' 0.1 ' ' 1

Analysis of similarites (ANOSIM)

**Cultivar**		**Timepoint**	
Dissimilarity: bray		Dissimilarity: bray	
ANOSIM statistic R	0.5467	ANOSIM statistic R	0.2337
Significance	1.00E-03	Significance	1.00E-03
Number of permutations: 9999		Number of permutations: 9999	

Dataset 2

Permutation test for homogeneity of multivariate dispersions

Cultivar

	Df	Sum Sq	Mean Sq	F	N.Perm	Pr(>F)	
Groups	3	0.00802	0.0026731	0.1397	999	0.962	
Residuals	32	0.61251	0.0191408				

Timepoint

	Df	Sum Sq	Mean Sq	F	N.Perm	Pr(>F)	
Groups	2	0.021351	0.0106756	29.481	999	0.047	*
Residuals	33	0.1195	0.0036212				

Pairwise comparisons for homogeneity of multivariate dispersions

(Observed p-value below diagonal, permuted p-value above diagonal)

	Agata	Fabiola	Hermes	Lady_Claire
Agata		0.903	0.666	0.726
Fabiola	0.9089		0.57	0.638
Hermes	0.6666	0.5795		0.925
Lady_Claire	0.74342	0.65714	0.93144	

	T0	T1	T2
T0		0.877	0.072
T1	0.869002		0.032
T2	0.082484	0.046653	

PERMANOVA on a distance matrix
9999 permutations

	Df	SumsOfSqs	MeanSqs	F.Model	R2	Pr(>F)	
Cultivar	3	0.30002	0.100006	12.991	0.10857	0.2177	.
Residuals	32	246.346	0.076983	0.89143			
Total	35	276.348	100.000				

	Df	SumsOfSqs	MeanSqs	F.Model	R2	Pr(>F)	
Timepoint	2	15.827	0.79136	22.117	0.57272	1.00E-04	***
Residuals	33	11.808	0.03578	0.42728			
Total	35	27.635	100.000				

Signif. codes: 0 ‘***’ 0.001 ‘**’ 0.01 ‘*’ 0.05 ‘.’ 0.1 ‘ ’ 1

Pairwise comparisons using PERMANOVAs on a distance matrix

9999 permutations

	Agata	Fabiola	Hermes
Fabiola	0.29	-	-
Hermes	0.64	0.35	-
Lady Claire	0.64	0.35	0.68

	T0	T1
T1	0.00300	-
T2	0.00015	0.00015

P value adjustment method: fdr

---

permutation CAP test on Bray Curtis dissimilarity matrix

	Df	SumsOfSqs	F	Pr(>F)	
Cultivar	3	0.30002	4.8266	0.001	***
Timepoint	2	158.271	38.1930	0.001	***
Cultivar:Timepoint	6	0	3.0846	0.001	***
Residual	24	0.49728			

Signif. codes: 0 ‘***’ 0.001 ‘**’ 0.01 ‘*’ 0.05 ‘.’ 0.1 ‘ ’ 1

---

multivariate GLM

	Res.	Df	Df.diff	Dev Pr(>Dev)
(Intercept)	35			
Timepoint	33	2	2210	0.001***

	Res.	Df	Df.diff	Dev
(Intercept)	35			
Cultivar	33	3	208.3	0.24

Signif. codes: 0 ‘***’ 0.001 ‘**’ 0.01 ‘*’ 0.05 ‘.’ 0.1 ‘ ’ 1

Analysis of similarities (ANOSIM)

Cultivar		Timepoint	
Dissimilarity: bray		Dissimilarity: bray	
ANOSIM statistic R	0.09414	ANOSIM statistic R	0.6821
Significance	4.36E-02	Significance	1.00E-04
Number of permutations: 9999		Number of permutations: 9999	

Dataset 4

Permutation test for homogeneity of multivariate dispersions

Cultivar

	Df	Sum Sq	Mean Sq	F	N.Perm	Pr(>F)	
Groups	6	0.060018	0.010003	41.684	999	0.003	**
Residuals	76	0.182378	0.0023997				

Tuber_part

	Df	Sum Sq	Mean Sq	F	N.Perm	Pr(>F)	
Groups	3	0.0256	0.0085329	0.945	999	0.434	
Residuals	79	0.71334	0.0090296				

Pairwise comparisons for homogeneity of multivariate dispersions

(Observed p-value below diagonal, permuted p-value above diagonal)

	Agata	Agria	Ditta	Fabiola	Fontane	Hermes	Lady Claire
Agata		0.214	0.152	0.218	0.593	0.455	0.165
Agria	0.20815525		0.001	0.001	0.266	0.022	0.8
Ditta	0.15702124	0.00063665		0.762	0.006	0.508	0.001
Fabiola	0.20336442	0.00038307	0.73916006		0.008	0.67	0.002
Fontane	0.57909921	0.26970761	0.00855392	0.00799809		0.102	0.185
Hermes	0.44029296	0.01704795	0.4948942	0.6448428	0.10315693		0.008
Lady Claire	0.17209841	0.77927156	0.00059068	0.00033791	0.19022587	0.01437224	

	corky epidermis	cortex	inner medulla	outer medulla
corky epidermis		0.925	0.184	0.809
cortex	0.92412		0.175	0.682
inner medulla	0.19596	0.1878		0.121
outer medulla	0.78547	0.69754	0.10727	

PERMANOVA on a distance matrix

9999 permutations

	Df	SumsOfSqs	MeanSqs	F.Model	R2	Pr(>F)	
Cultivar	6	42.661	0.71102	20.348	0.61634	1.00E-04	***
Residuals	76	26.556	0.03494		0.38366		
Total	82	69.217			100.000		

	Df	SumsOfSqs	MeanSqs	F.Model	R2	Pr(>F)	
Tuber_part	3	0.2231	0.074379	1	0.03224	0.5521	
Residuals	79	66.986	0.084792		0.96776		
Total	82	69.217			100.000		

Signif. codes: 0 ‘***’ 0.001 ‘**’ 0.01 ‘*’ 0.05 ‘.’ 0.1 ‘ ’ 1

Pairwise comparisons using PERMANOVAs on a distance matrix

9999 permutations

	Agata	Agria	Ditta	Fabiola	Fontane	Hermes
Agria	0.0001	-	-	-	-	-
Ditta	0.0001	0.0001	-	-	-	-
Fabiola	0.0001	0.0001	0.0001	-	-	-
Fontane	0.0001	0.0001	0.0001	0.0001	-	-
Hermes	0.0001	0.0001	0.0001	0.0001	0.0001	-
Lady Claire	0.0002	0.0001	0.0001	0.0001	0.0001	0.0001

	corky epidermis	cortex	inner medulla
cortex	0.91	-	-
inner medulla	0.67	0.77	-
outer medulla	0.91	0.91	0.67

P value adjustment method: fdr

---

permutation CAP test on Bray Curtis dissimilarity matrix

	Df	SumsOfSqs	F	Pr(>F)	
Cultivar	6	42.661	20.3719	0.001	***
Tuber_part	3	0	2.1872	0.017	*
Cultivar:Tuber_part	18	1	0.8145	0.883	
Residual	55	19.196			

Signif. codes: 0 ‘***’ 0.001 ‘**’ 0.01 ‘*’ 0.05 ‘.’ 0.1 ‘ ’ 1

---

multivariate GLM

	Res.	Df	Df.diff	Dev Pr(>Dev)	
(Intercept)	82				
Cultivar	76	6	4727	0.001	***

	Res.	Df	Df.diff	Dev Pr(>Dev)	
(Intercept)	82				
Tuber_part	79	3	200.2	0.387	

Signif. codes: 0 ‘***’ 0.001 ‘**’ 0.01 ‘*’ 0.05 ‘.’ 0.1 ‘ ’ 1

Analysis of similarities (ANOSIM)

Cultivar		Tuber part	
Dissimilarity: bray		Dissimilarity: bray	
ANOSIM statistic R	0.7415	ANOSIM statistic R	-0.0001237
Significance	1.00E-04	Significance	0.4222
Number of permutations: 9999		Number of permutations: 9999	
