# Supplementary material for: The bacterial community in potato is recruited from soil and partly inherited across generations
Source: PLoS One. 2019 Nov 8;14(11):e0223691. doi: 10.1371/journal.pone.0223691 (PMC6839881; doi:10.1371/journal.pone.0223691)
Supplement: S6 Table — Differentially abundant rOTUs of the bacterial communities based on the sequencing data from all potato tuber generations (T0-T2) are shown. Tubers were grown in commercial potting soil (dataset 2). (PDF) [file pone.0223691.s009.pdf]

Table S6: Taxonomic classification of differentially abundant rOTU. Differentially abundant rOTUs of bacterial community sequencing data of all potato tuber generation (T0-T2) are shown. Tubers were grown in commercial potting soil (dataset 2).

Differentially abundant OTUs	Taxonomy
OTU_1	p__Firmicutes;c__Bacilli;o__Bacillales;f__Bacillaceae;g__Bacillus
OTU_10	p__Proteobacteria;c__Betaproteobacteria;o__Burkholderiales;f__Burkholderiaceae;g__Ralstonia
OTU_100	p__Proteobacteria;c__Alphaproteobacteria;o__Caulobacterales;f__Caulobacteraceae;g__Caulobacter
OTU_105	p__Proteobacteria;c__Gammaproteobacteria;o__Xanthomonadales;f__Xanthomonadaceae;g__Dyella
OTU_108	p__Bacteroidetes;c__Sphingobacteriia;o__Sphingobacteriales;f__Chitinophagaceae;g__Terrimonas
OTU_109	p__Actinobacteria;c__Actinobacteria;o__Propionibacteriales;f__Propionibacteriaceae;g__Propionibacterium
OTU_1102	p__Bacteroidetes;c__Flavobacteriia;o__Flavobacteriales;f__Flavobacteriaceae;g__Flavobacterium
OTU_119	p__Proteobacteria;c__Alphaproteobacteria;o__Rhizobiales;f__Rhizobiaceae;g__Shinella
OTU_1252	p__Proteobacteria;c__Alphaproteobacteria;o__Rhizobiales;f__Hyphomicrobiaceae;g__Devosia
OTU_126	p__Bacteroidetes;c__Sphingobacteriia;o__Sphingobacteriales;f__Chitinophagaceae;g__Chitinophaga
OTU_13	p__Proteobacteria;c__Gammaproteobacteria;o__Pseudomonadales;f__Moraxellaceae;g__Acinetobacter
OTU_131	p__Actinobacteria;c__Actinobacteria;o__Streptosporangiales;f__Thermomonosporaceae
OTU_132	p__Proteobacteria;c__Alphaproteobacteria;o__Sphingomonadales
OTU_140	p__Proteobacteria;c__Alphaproteobacteria;o__Caulobacterales;f__Caulobacteraceae;g__Brevundimonas
OTU_146	p__Proteobacteria;c__Alphaproteobacteria;o__Caulobacterales;f__Caulobacteraceae;g__Asticcacaulis
OTU_1461	p__Actinobacteria;c__Actinobacteria;o__Streptomycetales;f__Streptomycetaceae;g__Streptomyces
OTU_147	p__Proteobacteria;c__Alphaproteobacteria;o__Rhizobiales;f__Bradyrhizobiaceae
OTU_15	p__Proteobacteria;c__Gammaproteobacteria;o__Pseudomonadales;f__Pseudomonadaceae;g__Pseudomonas
OTU_152	p__Proteobacteria;c__Gammaproteobacteria;o__Xanthomonadales;f__Xanthomonadaceae
OTU_158	p__Firmicutes;c__Bacilli;o__Bacillales
OTU_16	p__Bacteroidetes;c__Cytophagia;o__Cytophagales;f__Cytophagaceae;g__Emticicia
OTU_165	p__Proteobacteria;c__Gammaproteobacteria;o__Pseudomonadales;f__Moraxellaceae
OTU_17	p__Saccharibacteria;
OTU_178	p__Actinobacteria;c__Actinobacteria;o__Propionibacteriales;f__Nocardioidaceae;g__Kribbella
OTU_179	p__Proteobacteria;c__Alphaproteobacteria;o__Rhizobiales;f__Phyllobacteriaceae
OTU_18	p__Firmicutes;c__Bacilli;o__Bacillales
OTU_180	p__Actinobacteria;c__Actinobacteria;o__Micrococcales;f__Microbacteriaceae;g__Leifsonia
OTU_19	p__Proteobacteria;c__Alphaproteobacteria;o__Rhizobiales;f__Phyllobacteriaceae;g__Mesorhizobium
OTU_195	p__Proteobacteria;c__Alphaproteobacteria;o__Rhodospirillales;f__Rhodospirillaceae;g__Dongia
OTU_2	p__Proteobacteria;c__Betaproteobacteria;o__Burkholderiales;f__Burkholderiaceae;g__Burkholderia-Paraburkholderia
OTU_20	p__Bacteroidetes;c__Flavobacteriia;o__Flavobacteriales;f__Flavobacteriaceae;g__Chryseobacterium
OTU_203	p__Bacteroidetes;c__Sphingobacteriia;o__Sphingobacteriales;f__Chitinophagaceae;g__Chitinophaga
OTU_212	p__Proteobacteria;c__Betaproteobacteria;o__Burkholderiales;f__Comamonadaceae;g__Hydrogenophaga
OTU_22	p__Proteobacteria;c__Betaproteobacteria;o__Burkholderiales;f__Comamonadaceae;g__Variovorax
OTU_235	p__Proteobacteria;c__Gammaproteobacteria;o__Xanthomonadales;f__Xanthomonadaceae;g__Pseudoxanthomonas
OTU_238	p__Proteobacteria;c__Alphaproteobacteria;o__Rhizobiales;f__Bradyrhizobiaceae;g__Bosea
OTU_242	p__Proteobacteria;c__Alphaproteobacteria;o__Rhizobiales;f__Hyphomicrobiaceae;g__Devosia
OTU_25	p__Actinobacteria;c__Actinobacteria;o__Micrococcales;f__Micrococcaceae
OTU_265	p__Bacteroidetes;c__Sphingobacteriia;o__Sphingobacteriales;f__Chitinophagaceae;g__Parafilimonas

OTU_27	p__Actinobacteria;c__Actinobacteria;o__Streptomycetales;f__Streptomycetaceae;g__Streptomyces
OTU_270	p__Actinobacteria;c__Actinobacteria;o__Streptomycetales;f__Streptomycetaceae;g__Streptomyces
OTU_275	p__Bacteroidetes;c__Flavobacteriia;o__Flavobacteriales;f__Flavobacteriaceae;g__Flavobacterium
OTU_276	p__Proteobacteria;c__Alphaproteobacteria;o__Rhizobiales;f__Hyphomicrobiaceae;g__Devosia
OTU_278	p__Actinobacteria;c__Actinobacteria;o__Micrococcales;f__Microbacteriaceae;g__Frigoribacterium
OTU_2846	p__Actinobacteria;c__Actinobacteria;o__Micrococcales;f__Microbacteriaceae;g__Microbacterium
OTU_29	p__Proteobacteria;c__Gammaproteobacteria;o__Pseudomonadales;f__Moraxellaceae;g__Enhydrobacter
OTU_3	p__Proteobacteria;c__Alphaproteobacteria;o__Rhizobiales;f__Rhizobiaceae;g__Rhizobium
OTU_30	p__Saccharibacteria
OTU_319	p__Actinobacteria;c__Acidimicrobiia;o__Acidimicrobiales;f__Iamiaceae;g__Iamia
OTU_32	p__Actinobacteria;c__Actinobacteria;o__Corynebacteriales;f__Nocardiaceae;g__Rhodococcus
OTU_33	p__Proteobacteria;c__Betaproteobacteria;o__Burkholderiales;f__Comamonadaceae;0
OTU_35	p__Proteobacteria;c__Alphaproteobacteria;o__Caulobacterales;f__Caulobacteraceae;g__Asticcacaulis
OTU_350	p__Proteobacteria;c__Alphaproteobacteria;o__Rhizobiales;f__Xanthobacteraceae;g__Pseudolabrys
OTU_36	p__Bacteroidetes;c__Flavobacteriia;o__Flavobacteriales;f__Flavobacteriaceae;g__Flavobacterium
OTU_364	p__Proteobacteria;c__Alphaproteobacteria;o__Caulobacterales;f__Caulobacteraceae;g__Brevundimonas
OTU_38	p__Proteobacteria;c__Betaproteobacteria;o__Burkholderiales;f__Oxalobacteraceae;g__Massilia
OTU_39	p__Bacteroidetes;c__Sphingobacteriia;o__Sphingobacteriales;f__Chitinophagaceae;g__Terrimonas
OTU_392	p__Proteobacteria;c__Alphaproteobacteria;o__Sphingomonadales;f__Sphingomonadaceae;g__Sphingobium
OTU_4	p__Firmicutes;c__Bacilli;o__Bacillales;f__Staphylococcaceae;g__Staphylococcus
OTU_40	p__Actinobacteria;c__Actinobacteria;o__Micrococcales;f__Micrococcaceae;g__Glutamicibacter
OTU_41	p__Bacteroidetes;c__Sphingobacteriia;o__Sphingobacteriales;f__Chitinophagaceae
OTU_43	p__Bacteroidetes;c__Cytophagia;o__Cytophagales;f__Cytophagaceae
OTU_44	p__Bacteroidetes;c__Sphingobacteriia;o__Sphingobacteriales;f__Chitinophagaceae;g__Niastella
OTU_45	p__Proteobacteria;c__Gammaproteobacteria;o__Pseudomonadales;f__Pseudomonadaceae;g__Pseudomonas
OTU_47	p__Bacteroidetes;c__Sphingobacteriia;o__Sphingobacteriales;f__Sphingobacteriaceae;g__Pedobacter
OTU_48	p__Firmicutes;c__Bacilli;o__Bacillales;f__Planococcaceae;g__Lysinibacillus
OTU_49	p__Actinobacteria;c__Actinobacteria;o__Micrococcales;f__Micrococcaceae;g__Micrococcus
OTU_5	p__Actinobacteria;c__Actinobacteria;o__Micrococcales;f__Cellulomonadaceae;g__Cellulomonas
OTU_50	p__Proteobacteria;c__Betaproteobacteria;o__Methylophilales;f__Methylophilaceae
OTU_56	p__Actinobacteria;c__Actinobacteria;o__Micrococcales;f__Micrococcaceae;g__Arthrobacter
OTU_5735	p__Bacteroidetes;c__Flavobacteriia;o__Flavobacteriales;f__Flavobacteriaceae;g__Flavobacterium
OTU_58	p__Actinobacteria;c__Actinobacteria;o__Micrococcales;f__Microbacteriaceae
OTU_6	p__Proteobacteria;c__Betaproteobacteria;o__Burkholderiales;f__Comamonadaceae;g__Delftia
OTU_61	p__Bacteroidetes;c__Sphingobacteriia;o__Sphingobacteriales;f__Sphingobacteriaceae;g__Pedobacter
OTU_614	p__Proteobacteria;c__Betaproteobacteria;o__Burkholderiales;f__Burkholderiaceae;g__Cupriavidus
OTU_62	p__Bacteroidetes;c__Sphingobacteriia;o__Sphingobacteriales;f__Sphingobacteriaceae;g__Pedobacter
OTU_64	p__Proteobacteria;c__Alphaproteobacteria;o__Rhizobiales;f__Rhizobiaceae;g__Shinella
OTU_641	p__Proteobacteria;c__Alphaproteobacteria;o__Rhizobiales;f__Phyllobacteriaceae
OTU_65	p__Proteobacteria;c__Alphaproteobacteria;o__Rhizobiales;f__Rhizobiaceae;g__Rhizobium
OTU_67	p__Actinobacteria;c__Actinobacteria;o__Pseudonocardiales;f__Pseudonocardiaceae
OTU_68	p__Actinobacteria;c__Actinobacteria;o__Micrococcales;f__Microbacteriaceae
OTU_70	p__Actinobacteria;c__Actinobacteria;o__Streptomycetales;f__Streptomycetaceae;g__Streptomyces
OTU_700	p__Bacteroidetes;c__Sphingobacteriia;o__Sphingobacteriales;f__Chitinophagaceae;g__Flavitalea
OTU_73	p__Proteobacteria;c__Betaproteobacteria;o__Burkholderiales;f__Burkholderiaceae;g__Burkholderia-Paraburkholderia
OTU_74	p__Proteobacteria;c__Betaproteobacteria;o__Burkholderiales;f__Comamonadaceae
OTU_75	p__Bacteroidetes;c__Sphingobacteriia;o__Sphingobacteriales;f__Chitinophagaceae;g__Chitinophaga
OTU_76	p__Bacteroidetes;c__Sphingobacteriia;o__Sphingobacteriales;f__Sphingobacteriaceae;g__Mucilaginibacter

OTU_77	p__Actinobacteria;c__Actinobacteria;o__Micrococcales;f__Microbacteriaceae;g__Rudaibacter
OTU_8	p__Actinobacteria;c__Actinobacteria;o__Micrococcales;f__Micrococcaceae;g__Pseudarthrobacter
OTU_83	p__Proteobacteria;c__Alphaproteobacteria;o__Rhizobiales;f__Hyphomicrobiaceae;g__Devosia
OTU_85	p__Proteobacteria;c__Alphaproteobacteria;o__Sphingomonadales;f__Sphingomonadaceae;g__Sphingopyxis
OTU_86	p__Actinobacteria;c__Actinobacteria;o__Streptomycetales;f__Streptomycetaceae;g__Streptomyces
OTU_876	p__Actinobacteria;c__Actinobacteria;o__Micrococcales;f__Microbacteriaceae;g__Leifsonia
OTU_88	p__Proteobacteria;c__Alphaproteobacteria;o__Rhizobiales;f__Phyllobacteriaceae;g__Mesorhizobium
OTU_9	p__Actinobacteria;c__Actinobacteria;o__Micrococcales;f__Microbacteriaceae;g__Microbacterium
OTU_90	p__Bacteroidetes;c__Flavobacteriia;o__Flavobacteriales;f__Flavobacteriaceae;g__Chryseobacterium
OTU_91	p__Bacteroidetes;c__Sphingobacteriia;o__Sphingobacteriales;f__Chitinophagaceae
OTU_92	p__Proteobacteria;c__Alphaproteobacteria;o__Caulobacterales;f__Caulobacteraceae;g__Caulobacter
OTU_95	p__Actinobacteria;c__Actinobacteria;o__Streptomycetales;f__Streptomycetaceae;g__Streptomyces
OTU_97	p__Actinobacteria;c__Actinobacteria;o__Propionibacteriales;f__Nocardioidaceae
