# Supplementary material for: The bacterial community in potato is recruited from soil and partly inherited across generations
Source: PLoS One. 2019 Nov 8;14(11):e0223691. doi: 10.1371/journal.pone.0223691 (PMC6839881; doi:10.1371/journal.pone.0223691)
Supplement: S7 Table — Shared and unique rOTUs of the bacterial communities based on the sequencing data from three potato tuber generations (dataset 3) are shown in Venn diagrams (Fig 4). Samples were split according to soil types used for the cultivation of second generation tubers (T2). (PDF) [file pone.0223691.s010.pdf]

Table S7: **Taxonomic classification of shared and unique rOTUs.** Shared and unique rOTUs of bacterial community sequencing data of three potato tuber generations (dataset 3) are visualized in Venn diagrams (Fig 4). Samples were split according to soil types used for the cultivation of second generation tubers (T2).

		**potting soil**
T0	OTU_4	p__Firmicutes;c__Bacilli;o__Bacillales;f__Staphylococcaceae;g__Staphylococcus
	OTU_18	p__Firmicutes;c__Bacilli;o__Bacillales
	OTU_13	p__Proteobacteria;c__Gammaproteobacteria;o__Pseudomonadales;f__Moraxellaceae;g__Acinetobacter
	OTU_54	p__Firmicutes;c__Bacilli;o__Bacillales;f__Bacillaceae;g__Bacillus
T1	OTU_109	p__Actinobacteria;c__Actinobacteria;o__Propionibacteriales;f__Propionibacteriaceae;g__Propionibacterium
	OTU_35	p__Proteobacteria;c__Alphaproteobacteria;o__Caulobacterales;f__Caulobacteraceae;g__Asticcacaulis
	OTU_16	p__Bacteroidetes;c__Cytophagia;o__Cytophagales;f__Cytophagaceae;g__Emticicia
	OTU_90	p__Bacteroidetes;c__Flavobacteriia;o__Flavobacteriales;f__Flavobacteriaceae;g__Chryseobacterium
	OTU_45	p__Proteobacteria;c__Gammaproteobacteria;o__Pseudomonadales;f__Pseudomonadaceae;g__Pseudomonas
	OTU_67	p__Actinobacteria;c__Actinobacteria;o__Pseudonocardiales;f__Pseudonocardiaceae;
	OTU_155	p__Bacteroidetes;c__Sphingobacteriia;o__Sphingobacteriales;f__Chitinophagaceae;g__Chitinophaga
	OTU_5	p__Actinobacteria;c__Actinobacteria;o__Micrococcales;f__Cellulomonadaceae;g__Cellulomonas
	OTU_364	p__Proteobacteria;c__Alphaproteobacteria;o__Caulobacterales;f__Caulobacteraceae;g__Brevundimonas
	OTU_140	p__Proteobacteria;c__Alphaproteobacteria;o__Caulobacterales;f__Caulobacteraceae;g__Brevundimonas
	OTU_230	p__Actinobacteria;c__Acidimicrobiia;o__Acidimicrobiales;f__Iamiaceae;g__Iamia
	OTU_2846	p__Actinobacteria;c__Actinobacteria;o__Micrococcales;f__Microbacteriaceae;g__Microbacterium
	OTU_75	p__Bacteroidetes;c__Sphingobacteriia;o__Sphingobacteriales;f__Chitinophagaceae;g__Chitinophaga
	OTU_527	p__Proteobacteria;c__Alphaproteobacteria;o__Sphingomonadales;f__Erythrobacteraceae;g__Altererythrobacter
	OTU_85	p__Proteobacteria;c__Alphaproteobacteria;o__Sphingomonadales;f__Sphingomonadaceae;g__Sphingopyxis
	OTU_601	p__Proteobacteria;c__Betaproteobacteria;o__Burkholderiales;f__Comamonadaceae;
	OTU_618	p__Proteobacteria;c__Alphaproteobacteria;o__Rhizobiales;f__Methylobacteriaceae;
	OTU_252	p__Proteobacteria;c__Alphaproteobacteria;o__Rhizobiales;f__Brucellaceae;g__Ochrobactrum
	OTU_1267	p__Proteobacteria;c__Alphaproteobacteria;o__Rhizobiales
	OTU_1600	p__Proteobacteria;c__Alphaproteobacteria;o__Rhizobiales;f__Hyphomicrobiaceae;
T1 potting soil	OTU_33	p__Proteobacteria;c__Betaproteobacteria;o__Burkholderiales;f__Comamonadaceae;
	OTU_41	p__Bacteroidetes;c__Sphingobacteriia;o__Sphingobacteriales;f__Chitinophagaceae;
	OTU_39	p__Bacteroidetes;c__Sphingobacteriia;o__Sphingobacteriales;f__Chitinophagaceae;g__Terrimonas
	OTU_86	p__Actinobacteria;c__Actinobacteria;o__Streptomycetales;f__Streptomycetaceae;g__Streptomyces
	OTU_30	p__Saccharibacteria
	OTU_180	p__Actinobacteria;c__Actinobacteria;o__Micrococcales;f__Microbacteriaceae;g__Leifsonia
	OTU_142	p__Proteobacteria;c__Alphaproteobacteria;o__Caulobacterales;f__Caulobacteraceae;
	OTU_270	p__Actinobacteria;c__Actinobacteria;o__Streptomycetales;f__Streptomycetaceae;g__Streptomyces
	OTU_1461	p__Actinobacteria;c__Actinobacteria;o__Streptomycetales;f__Streptomycetaceae;g__Streptomyces
	OTU_425	p__Actinobacteria;c__Actinobacteria;o__Streptomycetales;f__Streptomycetaceae;g__Streptomyces
	OTU_95	p__Actinobacteria;c__Actinobacteria;o__Streptomycetales;f__Streptomycetaceae;g__Streptomyces
	OTU_126	p__Bacteroidetes;c__Sphingobacteriia;o__Sphingobacteriales;f__Chitinophagaceae;g__Chitinophaga
	OTU_282	p__Chloroflexi;c__S85;
	OTU_77	p__Actinobacteria;c__Actinobacteria;o__Micrococcales;f__Microbacteriaceae;g__Rudaibacter
	OTU_605	p__Proteobacteria;c__Alphaproteobacteria;o__Rhizobiales;f__Hyphomicrobiaceae;g__Hyphomicrobium
	OTU_5498	p__Proteobacteria;c__Alphaproteobacteria;o__Rhizobiales;f__Xanthobacteraceae;g__Variibacter
	OTU_757	p__Actinobacteria;c__Actinobacteria;o__Streptomycetales;f__Streptomycetaceae;g__Streptomyces
	OTU_138	p__Actinobacteria;c__Actinobacteria;o__Micrococcales;f__Microbacteriaceae;
	OTU_53	p__Proteobacteria;c__Alphaproteobacteria;o__Caulobacterales;f__Caulobacteraceae;g__Caulobacter
	OTU_2313	p__Actinobacteria;c__Actinobacteria;o__Propionibacteriales;f__Nocardioidaceae;g__Marmoricola
	OTU_1606	p__Proteobacteria;c__Betaproteobacteria;o__Burkholderiales;f__Comamonadaceae;
	OTU_266	p__Bacteroidetes;c__Sphingobacteriia;o__Sphingobacteriales;f__Chitinophagaceae;
	OTU_166	p__Saccharibacteria
	OTU_203	p__Bacteroidetes;c__Sphingobacteriia;o__Sphingobacteriales;f__Chitinophagaceae;g__Chitinophaga
	OTU_111	p__Bacteroidetes;c__Cytophagia;o__Cytophagales;f__Cytophagaceae;g__Ohtaekwangia
	OTU_654	p__Bacteroidetes;c__Sphingobacteriia;o__Sphingobacteriales;f__Chitinophagaceae;
	OTU_731	p__Actinobacteria;c__Actinobacteria;o__Propionibacteriales;f__Nocardioidaceae;g__Marmoricola
	OTU_172	p__Proteobacteria;c__Betaproteobacteria;o__Rhodocyclales;f__Rhodocyclaceae;
	OTU_246	p__Actinobacteria;c__Actinobacteria;o__Propionibacteriales;f__Nocardioidaceae;g__Nocardioides
	OTU_533	p__Actinobacteria;c__Thermoleophilia;o__Solirubrobacterales;f__Elev-16S-1332;
	OTU_340	p__Proteobacteria;c__Gammaproteobacteria;o__Pseudomonadales;f__Moraxellaceae;
	OTU_700	p__Bacteroidetes;c__Sphingobacteriia;o__Sphingobacteriales;f__Chitinophagaceae;g__Flavitalea
	OTU_3612	p__Actinobacteria;c__Actinobacteria;o__Corynebacteriales;f__Mycobacteriaceae;g__Mycobacterium
	OTU_1124	p__Saccharibacteria
	OTU_51	p__Proteobacteria;c__Alphaproteobacteria;o__Sphingomonadales;f__Sphingomonadaceae;g__Sphingomonas

OTU_1382	p__Actinobacteria;c__Actinobacteria;o__Micrococcales;f__Intrasporangiaceae;
OTU_209	p__Proteobacteria;c__Betaproteobacteria;o__Burkholderiales;f__Alcaligenaceae;
OTU_92	p__Proteobacteria;c__Alphaproteobacteria;o__Caulobacterales;f__Caulobacteraceae;g__Caulobacter
OTU_381	p__Bacteroidetes;c__Sphingobacteriia;o__Sphingobacteriales;f__Chitinophagaceae;g__Niastella
OTU_91	p__Bacteroidetes;c__Sphingobacteriia;o__Sphingobacteriales;f__Chitinophagaceae;
OTU_291	p__Bacteroidetes;c__Cytophagia;o__Cytophagales;f__Cytophagaceae;g__Cytophaga
OTU_728	p__Actinobacteria;c__Acidimicrobiia;o__Acidimicrobiales
OTU_338	p__Proteobacteria;c__Gammaproteobacteria;o__Legionellales;f__Legionellaceae;g__Legionella
OTU_8914	p__Actinobacteria;c__Actinobacteria;o__Propionibacteriales;f__Nocardioidaceae;
OTU_79	p__Thaumarchaeota;c__Soil Crenarchaeotic Group(SCG)
OTU_588	p__Proteobacteria;c__Alphaproteobacteria;o__Rhizobiales;f__Xanthobacteraceae;g__Variibacter
OTU_570	p__Proteobacteria;c__Alphaproteobacteria;o__Caulobacterales;f__Caulobacteraceae;g__Phenylobacterium
OTU_128	p__Bacteroidetes;c__Sphingobacteriia;o__Sphingobacteriales;f__Sphingobacteriaceae;
OTU_1215	p__Bacteroidetes;c__Sphingobacteriia;o__Sphingobacteriales;f__Chitinophagaceae;
OTU_42	p__Firmicutes;c__Bacilli;o__Bacillales;f__Paenibacillaceae;g__Paenibacillus
OTU_1141	p__Actinobacteria;c__Acidimicrobiia;o__Acidimicrobiales;f__Acidimicrobiaceae;g__CL5-29 marine group
OTU_478	p__Proteobacteria;c__Betaproteobacteria;o__Burkholderiales;f__Comamonadaceae;g__Ramlibacter
OTU_296	p__Proteobacteria;c__Betaproteobacteria;o__Nitrosomonadales;f__Nitrosomonadaceae;
OTU_247	p__Proteobacteria;c__Alphaproteobacteria;o__Caulobacterales;f__Caulobacteraceae;g__Phenylobacterium
OTU_348	p__Actinobacteria;c__Actinobacteria;o__Micromonosporales;f__Micromonosporaceae;
OTU_186	p__Proteobacteria;c__Alphaproteobacteria;o__Rhizobiales;f__Bradyrhizobiaceae;g__Bradyrhizobium
OTU_104	p__Proteobacteria;c__Gammaproteobacteria;o__Cellvibrionales;f__Cellvibrionaceae;g__Cellvibrio
OTU_474	p__Actinobacteria;c__Actinobacteria;o__Micrococcales;f__Microbacteriaceae;
OTU_480	p__Gemmatimonadetes;c__Gemmatimonadetes;o__Gemmatimonadales;f__Gemmatimonadaceae;
OTU_410	p__Bacteroidetes;c__Cytophagia;o__Cytophagales;f__Cytophagaceae;g__Chryseolinea
OTU_352	p__Proteobacteria;c__Betaproteobacteria;o__Burkholderiales;f__Oxalobacteraceae;
OTU_123	p__Actinobacteria;c__Actinobacteria;o__Propionibacteriales;f__Nocardioidaceae;g__Nocardioides
OTU_517	p__Proteobacteria;c__Alphaproteobacteria;o__Caulobacterales;f__Caulobacteraceae;
OTU_607	p__Proteobacteria;c__Betaproteobacteria;o__TRA3-2;
OTU_312	p__Bacteroidetes;c__Sphingobacteriia;o__Sphingobacteriales;f__Chitinophagaceae;g__Parafilimonas
OTU_272	p__Actinobacteria;c__Actinobacteria;o__Streptomycetales;f__Streptomycetaceae;g__Streptomyces
OTU_3454	p__Bacteroidetes;c__Sphingobacteriia;o__Sphingobacteriales;f__Chitinophagaceae;
OTU_715	p__Proteobacteria;c__Gammaproteobacteria;o__Xanthomonadales;f__Xanthomonadales Incertae Sedis;g__Acidibacter
OTU_863	p__Actinobacteria;c__Acidimicrobiia;o__Acidimicrobiales
OTU_2167	p__Proteobacteria;c__Alphaproteobacteria;o__Rhizobiales;f__Rhizobiales Incertae Sedis;g__Nordella
OTU_426	p__Proteobacteria;c__Betaproteobacteria;o__Burkholderiales;f__Comamonadaceae;
OTU_528	p__Proteobacteria;c__Alphaproteobacteria;o__Caulobacterales;f__Hyphomonadaceae;g__Hirschia
OTU_873	p__Proteobacteria;c__Alphaproteobacteria;o__Rhizobiales;f__Hyphomicrobiaceae;
OTU_1085	p__Proteobacteria;c__Deltaproteobacteria;o__Myxococcales;f__Sandaracinaceae;
OTU_248	p__Gemmatimonadetes;c__Gemmatimonadetes;o__Gemmatimonadales;f__Gemmatimonadaceae;
OTU_538	p__Actinobacteria;c__Actinobacteria;o__Micromonosporales;f__Micromonosporaceae;
OTU_998	p__Actinobacteria;c__Actinobacteria;o__Frankiales;f__Sporichthyaceae;g__Sporichthya
OTU_2895	p__Proteobacteria;c__Alphaproteobacteria;o__Rhodospirillales;f__Acetobacteraceae;g__Roseomonas
OTU_2466	p__Proteobacteria;c__Alphaproteobacteria;o__Rhizobiales;f__Hyphomicrobiaceae;g__Devosia
OTU_1086	p__Proteobacteria;c__Alphaproteobacteria;o__Rhizobiales;f__JG34-KF-361;
OTU_268	p__Proteobacteria;c__Gammaproteobacteria;o__Xanthomonadales;f__Xanthomonadales Incertae Sedis;g__Acidibacter
OTU_133	p__Bacteroidetes;c__Sphingobacteriia;o__Sphingobacteriales;f__Chitinophagaceae;g__Terrimonas
OTU_437	p__Actinobacteria;c__Thermoleophilia;o__Solirubrobacterales;f__TM146;
OTU_418	p__Actinobacteria;c__Acidimicrobiia;o__Acidimicrobiales;f__Acidimicrobiaceae;g__Ilumatobacter
OTU_1046	p__Proteobacteria;c__Alphaproteobacteria;o__Rhizobiales;f__Xanthobacteraceae;g__Variibacter
OTU_713	p__Chloroflexi;c__TK1
OTU_565	p__Proteobacteria;c__Alphaproteobacteria;o__Rhodospirillales;f__Rhodospirillales Incertae Sedis;g__Reyranella
OTU_1200	p__Actinobacteria;c__Acidimicrobiia;o__Acidimicrobiales;f__Iamiaceae;g__Iamia
OTU_1180	p__Actinobacteria;c__Actinobacteria;o__Propionibacteriales;f__Nocardioidaceae;g__Nocardioides
OTU_188	p__Proteobacteria;c__Gammaproteobacteria;o__Xanthomonadales;f__Xanthomonadales Incertae Sedis;
OTU_22371	p__Actinobacteria;c__Actinobacteria;o__Micrococcales;f__Microbacteriaceae;g__Microbacterium
OTU_749	p__Proteobacteria;c__Alphaproteobacteria;o__Rhizobiales;f__Rhizobiales Incertae Sedis;g__Nordella
OTU_377	p__Proteobacteria;c__Gammaproteobacteria;o__Xanthomonadales
OTU_785	p__Actinobacteria;c__Acidimicrobiia;o__Acidimicrobiales;f__Iamiaceae;g__Iamia
OTU_560	p__Proteobacteria;c__Alphaproteobacteria;o__Rhodospirillales;f__Rhodospirillaceae;g__Ferrovibrio
OTU_318	p__Actinobacteria;c__Actinobacteria;o__Corynebacteriales;f__Mycobacteriaceae;g__Mycobacterium
OTU_941	p__Proteobacteria;c__Alphaproteobacteria;o__Rhizobiales;f__Methylobacteriaceae;
OTU_529	p__Actinobacteria;c__Thermoleophilia;o__Solirubrobacterales;f__Elev-16S-1332;
OTU_380	p__Bacteroidetes;c__Cytophagia;o__Cytophagales;f__Cytophagaceae;
OTU_168	p__Proteobacteria;c__Alphaproteobacteria;o__Rhodospirillales;f__Rhodospirillaceae;

	OTU_833	p__FBP;
	OTU_1604	p__Proteobacteria;c__Alphaproteobacteria;o__Rhizobiales;f__BCf3-2;
	OTU_502	p__Proteobacteria;c__Alphaproteobacteria;o__Rhizobiales;f__Xanthobacteraceae;g__Variibacter
	OTU_78	p__Bacteroidetes;c__Cytophagia;o__Cytophagales;f__Cytophagaceae;
	OTU_5804	p__Proteobacteria;c__Gammaproteobacteria;o__Xanthomonadales;f__Xanthomonadales Incertae Sedis;
	OTU_660	p__Proteobacteria;c__Betaproteobacteria;o__Nitrosomonadales;f__Nitrosomonadaceae;
	OTU_681	p__Proteobacteria;c__Alphaproteobacteria;o__Rhizobiales;f__Hyphomicrobiaceae;g__Hyphomicrobium
	OTU_261	p__Proteobacteria;c__Betaproteobacteria;o__Nitrosomonadales;f__Nitrosomonadaceae;
	OTU_899	p__Chloroflexi;c__TK1
	OTU_1199	p__Armatimonadetes;c__Fimbriimonadia;o__Fimbriimonadales;f__Fimbriimonadaceae;
	OTU_5027	p__Proteobacteria;c__Alphaproteobacteria;o__Rhodospirillales;f__Rhodospirillaceae;g__Dongia
	OTU_3557	p__Chloroflexi;c__S85
	OTU_1024	p__Proteobacteria;c__Betaproteobacteria;o__TRA3-2
	OTU_875	p__Proteobacteria;c__Deltaproteobacteria;o__Myxococcales;f__Sandaracinaceae;
	OTU_1143	p__Actinobacteria;c__Actinobacteria;o__Micrococcales;f__Intrasporangiaceae;g__Intrasporangium
	OTU_1602	p__Bacteroidetes;c__Cytophagia;o__Cytophagales;f__Cytophagaceae;
	OTU_716	p__Spirochaetae;c__Spirochaetes;o__Spirochaetales;f__Spirochaetaceae;
	OTU_2257	p__Saccharibacteria;
T0|T1	OTU_20	p__Bacteroidetes;c__Flavobacteriia;o__Flavobacteriales;f__Flavobacteriaceae;g__Chryseobacterium
	OTU_15	p__Proteobacteria;c__Gammaproteobacteria;o__Pseudomonadales;f__Pseudomonadaceae;g__Pseudomonas
	OTU_3	p__Proteobacteria;c__Alphaproteobacteria;o__Rhizobiales;f__Rhizobiaceae;g__Rhizobium
	OTU_19	p__Proteobacteria;c__Alphaproteobacteria;o__Rhizobiales;f__Phyllobacteriaceae;g__Mesorhizobium
T1|T2 potting soil	OTU_27	p__Actinobacteria;c__Actinobacteria;o__Streptomycetales;f__Streptomycetaceae;g__Streptomyces
	OTU_58	p__Actinobacteria;c__Actinobacteria;o__Micrococcales;f__Microbacteriaceae;
	OTU_9	p__Actinobacteria;c__Actinobacteria;o__Micrococcales;f__Microbacteriaceae;g__Microbacterium
	OTU_56	p__Actinobacteria;c__Actinobacteria;o__Micrococcales;f__Micrococcaceae;g__Arthrobacter
	OTU_17	p__Saccharibacteria;
	OTU_10	p__Proteobacteria;c__Betaproteobacteria;o__Burkholderiales;f__Burkholderiaceae;g__Ralstonia
	OTU_43	p__Bacteroidetes;c__Cytophagia;o__Cytophagales;f__Cytophagaceae;
	OTU_468	p__Proteobacteria;
	OTU_50	p__Proteobacteria;c__Betaproteobacteria;o__Methylophilales;f__Methylophilaceae;
	OTU_68	p__Actinobacteria;c__Actinobacteria;o__Micrococcales;f__Microbacteriaceae;
	OTU_1252	p__Proteobacteria;c__Alphaproteobacteria;o__Rhizobiales;f__Hyphomicrobiaceae;g__Devosia
	OTU_464	p__Actinobacteria;c__Acidimicrobiia;o__Acidimicrobiales
	OTU_755	p__Actinobacteria;c__Acidimicrobiia;o__Acidimicrobiales
	OTU_131	p__Actinobacteria;c__Actinobacteria;o__Streptosporangiales;f__Thermomonosporaceae;
	OTU_44	p__Bacteroidetes;c__Sphingobacteriia;o__Sphingobacteriales;f__Chitinophagaceae;g__Niastella
	OTU_119	p__Proteobacteria;c__Alphaproteobacteria;o__Rhizobiales;f__Rhizobiaceae;g__Shinella
	OTU_319	p__Actinobacteria;c__Acidimicrobiia;o__Acidimicrobiales;f__Iamiaceae;g__Iamia
	OTU_350	p__Proteobacteria;c__Alphaproteobacteria;o__Rhizobiales;f__Xanthobacteraceae;g__Pseudolabrys
	OTU_88	p__Proteobacteria;c__Alphaproteobacteria;o__Rhizobiales;f__Phyllobacteriaceae;g__Mesorhizobium
	OTU_65	p__Proteobacteria;c__Alphaproteobacteria;o__Rhizobiales;f__Rhizobiaceae;g__Rhizobium
	OTU_238	p__Proteobacteria;c__Alphaproteobacteria;o__Rhizobiales;f__Bradyrhizobiaceae;g__Bosea
	OTU_876	p__Actinobacteria;c__Actinobacteria;o__Micrococcales;f__Microbacteriaceae;g__Leifsonia
	OTU_212	p__Proteobacteria;c__Betaproteobacteria;o__Burkholderiales;f__Comamonadaceae;g__Hydrogenophaga
	OTU_83	p__Proteobacteria;c__Alphaproteobacteria;o__Rhizobiales;f__Hyphomicrobiaceae;g__Devosia
	OTU_147	p__Proteobacteria;c__Alphaproteobacteria;o__Rhizobiales;f__Bradyrhizobiaceae;
	OTU_392	p__Proteobacteria;c__Alphaproteobacteria;o__Sphingomonadales;f__Sphingomonadaceae;g__Sphingobium
	OTU_242	p__Proteobacteria;c__Alphaproteobacteria;o__Rhizobiales;f__Hyphomicrobiaceae;g__Devosia
	OTU_195	p__Proteobacteria;c__Alphaproteobacteria;o__Rhodospirillales;f__Rhodospirillaceae;g__Dongia
	OTU_165	p__Proteobacteria;c__Gammaproteobacteria;o__Pseudomonadales;f__Moraxellaceae;
	OTU_235	p__Proteobacteria;c__Gammaproteobacteria;o__Xanthomonadales;f__Xanthomonadaceae;g__Pseudoxanthomonas
	OTU_194	p__Proteobacteria;c__Alphaproteobacteria;o__Sphingomonadales;f__Sphingomonadaceae;g__Novosphingobium
	OTU_509	p__Actinobacteria;c__Thermoleophilia;o__Solirubrobacterales;f__Elev-16S-1332;
	OTU_518	p__Proteobacteria;c__Alphaproteobacteria;o__Rhizobiales;f__Rhizobiales Incertae Sedis;g__Bauldia
	OTU_64	p__Proteobacteria;c__Alphaproteobacteria;o__Rhizobiales;f__Rhizobiaceae;g__Shinella
	OTU_178	p__Actinobacteria;c__Actinobacteria;o__Propionibacteriales;f__Nocardioidaceae;g__Kribbella
	OTU_641	p__Proteobacteria;c__Alphaproteobacteria;o__Rhizobiales;f__Phyllobacteriaceae;
	OTU_148	p__Proteobacteria;c__Alphaproteobacteria;o__Rhizobiales;f__Hyphomicrobiaceae;g__Devosia
	OTU_108	p__Bacteroidetes;c__Sphingobacteriia;o__Sphingobacteriales;f__Chitinophagaceae;g__Terrimonas
	OTU_505	p__Proteobacteria;c__Alphaproteobacteria;o__Rhodospirillales;f__Rhodospirillales Incertae Sedis;g__Reyranella
	OTU_501	p__Actinobacteria;c__Acidimicrobiia;o__Acidimicrobiales;f__Acidimicrobiaceae;
	OTU_223	p__Actinobacteria;c__Acidimicrobiia;o__Acidimicrobiales;f__Iamiaceae;g__Iamia
	OTU_179	p__Proteobacteria;c__Alphaproteobacteria;o__Rhizobiales;f__Phyllobacteriaceae;
	OTU_69	p__Actinobacteria;c__Actinobacteria;o__Propionibacteriales;f__Nocardioidaceae;g__Nocardioides
	OTU_1094	p__Proteobacteria;c__Gammaproteobacteria;o__Xanthomonadales;f__Xanthomonadaceae;g__Dokdonella
	OTU_141	p__Actinobacteria;c__Thermoleophilia;o__Solirubrobacterales;f__Gsoil-1167;

	OTU_4010	p__Proteobacteria;c__Alphaproteobacteria;o__Rhizobiales;f__Hyphomicrobiaceae;g__Devosia
	OTU_174	p__Actinobacteria;c__Actinobacteria;o__Corynebacteriales;f__Mycobacteriaceae;g__Mycobacterium
	OTU_1288	p__Proteobacteria;c__Alphaproteobacteria;o__Rhizobiales;f__Rhizobiales Incertae Sedis;g__Bauldia
	OTU_357	p__Proteobacteria;c__Alphaproteobacteria;o__Rhizobiales;f__Rhizobiales Incertae Sedis;g__Rhizomicrobium
T0|T1|T2 potting soil	OTU_97	p__Actinobacteria;c__Actinobacteria;o__Propionibacteriales;f__Nocardioidaceae;
	OTU_8	p__Actinobacteria;c__Actinobacteria;o__Micrococcales;f__Micrococcaceae;g__Pseudarthrobacter
	OTU_1	p__Firmicutes;c__Bacilli;o__Bacillales;f__Bacillaceae;g__Bacillus
	OTU_22	p__Proteobacteria;c__Betaproteobacteria;o__Burkholderiales;f__Comamonadaceae;g__Variovorax
	OTU_100	p__Proteobacteria;c__Alphaproteobacteria;o__Caulobacterales;f__Caulobacteraceae;g__Caulobacter

		**Tulln**
T0	OTU_4	p__Firmicutes;c__Bacilli;o__Bacillales;f__Staphylococcaceae;g__Staphylococcus
	OTU_13	p__Proteobacteria;c__Gammaproteobacteria;o__Pseudomonadales;f__Moraxellaceae;g__Acinetobacter
T1	OTU_58	p__Actinobacteria;c__Actinobacteria;o__Micrococcales;f__Microbacteriaceae;
	OTU_109	p__Actinobacteria;c__Actinobacteria;o__Propionibacteriales;f__Propionibacteriaceae;g__Propionibacterium
	OTU_17	p__Saccharibacteria;
	OTU_10	p__Proteobacteria;c__Betaproteobacteria;o__Burkholderiales;f__Burkholderiaceae;g__Ralstonia
	OTU_35	p__Proteobacteria;c__Alphaproteobacteria;o__Caulobacterales;f__Caulobacteraceae;g__Asticcacaulis
	OTU_43	p__Bacteroidetes;c__Cytophagia;o__Cytophagales;f__Cytophagaceae;
	OTU_16	p__Bacteroidetes;c__Cytophagia;o__Cytophagales;f__Cytophagaceae;g__Emticicia
	OTU_468	p__Proteobacteria;
	OTU_90	p__Bacteroidetes;c__Flavobacteriia;o__Flavobacteriales;f__Flavobacteriaceae;g__Chryseobacterium
	OTU_464	p__Actinobacteria;c__Acidimicrobiia;o__Acidimicrobiales
	OTU_755	p__Actinobacteria;c__Acidimicrobiia;o__Acidimicrobiales
	OTU_131	p__Actinobacteria;c__Actinobacteria;o__Streptosporangiales;f__Thermomonosporaceae;
	OTU_155	p__Bacteroidetes;c__Sphingobacteriia;o__Sphingobacteriales;f__Chitinophagaceae;g__Chitinophaga
	OTU_119	p__Proteobacteria;c__Alphaproteobacteria;o__Rhizobiales;f__Rhizobiaceae;g__Shinella
	OTU_319	p__Actinobacteria;c__Acidimicrobiia;o__Acidimicrobiales;f__Iamiaceae;g__Iamia
	OTU_350	p__Proteobacteria;c__Alphaproteobacteria;o__Rhizobiales;f__Xanthobacteraceae;g__Pseudolabrys
	OTU_5	p__Actinobacteria;c__Actinobacteria;o__Micrococcales;f__Cellulomonadaceae;g__Cellulomonas
	OTU_65	p__Proteobacteria;c__Alphaproteobacteria;o__Rhizobiales;f__Rhizobiaceae;g__Rhizobium
	OTU_364	p__Proteobacteria;c__Alphaproteobacteria;o__Caulobacterales;f__Caulobacteraceae;g__Brevundimonas
	OTU_876	p__Actinobacteria;c__Actinobacteria;o__Micrococcales;f__Microbacteriaceae;g__Leifsonia
	OTU_194	p__Proteobacteria;c__Alphaproteobacteria;o__Sphingomonadales;f__Sphingomonadaceae;g__Novosphingobium
	OTU_509	p__Actinobacteria;c__Thermoleophilia;o__Solirubrobacterales;f__Elev-16S-1332;
	OTU_140	p__Proteobacteria;c__Alphaproteobacteria;o__Caulobacterales;f__Caulobacteraceae;g__Brevundimonas
	OTU_518	p__Proteobacteria;c__Alphaproteobacteria;o__Rhizobiales;f__Rhizobiales Incertae Sedis;g__Bauldia
	OTU_230	p__Actinobacteria;c__Acidimicrobiia;o__Acidimicrobiales;f__Iamiaceae;g__Iamia
	OTU_178	p__Actinobacteria;c__Actinobacteria;o__Propionibacteriales;f__Nocardioidaceae;g__Kribbella
	OTU_2846	p__Actinobacteria;c__Actinobacteria;o__Micrococcales;f__Microbacteriaceae;g__Microbacterium
	OTU_75	p__Bacteroidetes;c__Sphingobacteriia;o__Sphingobacteriales;f__Chitinophagaceae;g__Chitinophaga
	OTU_108	p__Bacteroidetes;c__Sphingobacteriia;o__Sphingobacteriales;f__Chitinophagaceae;g__Terrimonas
	OTU_505	p__Proteobacteria;c__Alphaproteobacteria;o__Rhodospirillales;f__Rhodospirillales Incertae Sedis;g__Reyranella
	OTU_601	p__Proteobacteria;c__Betaproteobacteria;o__Burkholderiales;f__Comamonadaceae;
	OTU_618	p__Proteobacteria;c__Alphaproteobacteria;o__Rhizobiales;f__Methylobacteriaceae;
	OTU_252	p__Proteobacteria;c__Alphaproteobacteria;o__Rhizobiales;f__Brucellaceae;g__Ochrobactrum
	OTU_1094	p__Proteobacteria;c__Gammaproteobacteria;o__Xanthomonadales;f__Xanthomonadaceae;g__Dokdonella
	OTU_1267	p__Proteobacteria;c__Alphaproteobacteria;o__Rhizobiales
	OTU_1600	p__Proteobacteria;c__Alphaproteobacteria;o__Rhizobiales;f__Hyphomicrobiaceae;
	OTU_357	p__Proteobacteria;c__Alphaproteobacteria;o__Rhizobiales;f__Rhizobiales Incertae Sedis;g__Rhizomicrobium
T2 Tulln	OTU_33	p__Proteobacteria;c__Betaproteobacteria;o__Burkholderiales;f__Comamonadaceae;
	OTU_39	p__Bacteroidetes;c__Sphingobacteriia;o__Sphingobacteriales;f__Chitinophagaceae;g__Terrimonas
	OTU_30	p__Saccharibacteria;
	OTU_28	p__Actinobacteria;c__Actinobacteria;o__Micrococcales;f__Microbacteriaceae;g__Agromyces
	OTU_34	p__Actinobacteria;c__Actinobacteria;o__Micrococcales;f__Micrococcaceae;
	OTU_142	p__Proteobacteria;c__Alphaproteobacteria;o__Caulobacterales;f__Caulobacteraceae;
	OTU_449	p__Proteobacteria;c__Betaproteobacteria;o__Nitrosomonadales;f__Nitrosomonadaceae;
	OTU_1461	p__Actinobacteria;c__Actinobacteria;o__Streptomycetales;f__Streptomycetaceae;g__Streptomyces
	OTU_95	p__Actinobacteria;c__Actinobacteria;o__Streptomycetales;f__Streptomycetaceae;g__Streptomyces
	OTU_25	p__Actinobacteria;c__Actinobacteria;o__Micrococcales;f__Micrococcaceae;
	OTU_1506	p__Bacteroidetes;c__Sphingobacteriia;o__Sphingobacteriales;f__Chitinophagaceae;
	OTU_126	p__Bacteroidetes;c__Sphingobacteriia;o__Sphingobacteriales;f__Chitinophagaceae;g__Chitinophaga
	OTU_282	p__Chloroflexi;c__S85
	OTU_221	p__Proteobacteria;c__Alphaproteobacteria;o__Rhizobiales;f__Rhizobiaceae;
	OTU_407	p__Proteobacteria;c__Betaproteobacteria;o__Burkholderiales;f__Oxalobacteraceae;
	OTU_5498	p__Proteobacteria;c__Alphaproteobacteria;o__Rhizobiales;f__Xanthobacteraceae;g__Variibacter

OTU_225	p__Proteobacteria;c__Betaproteobacteria;o__Burkholderiales;f__Oxalobacteraceae;g__Paucimonas
OTU_757	p__Actinobacteria;c__Actinobacteria;o__Streptomycetales;f__Streptomycetaceae;g__Streptomyces
OTU_773	p__Proteobacteria;c__Betaproteobacteria;o__Burkholderiales;f__Comamonadaceae;
OTU_297	p__Proteobacteria;c__Betaproteobacteria;o__Burkholderiales;f__Oxalobacteraceae;
OTU_596	p__Bacteroidetes;c__Cytophagia;o__Cytophagales;f__Cytophagaceae;g__Ohtaekwangia
OTU_280	p__Actinobacteria;c__Actinobacteria;o__Frankiales;f__Geodermatophilaceae;g__Blastococcus
OTU_53	p__Proteobacteria;c__Alphaproteobacteria;o__Caulobacterales;f__Caulobacteraceae;g__Caulobacter
OTU_266	p__Bacteroidetes;c__Sphingobacteriia;o__Sphingobacteriales;f__Chitinophagaceae;
OTU_203	p__Bacteroidetes;c__Sphingobacteriia;o__Sphingobacteriales;f__Chitinophagaceae;g__Chitinophaga
OTU_111	p__Bacteroidetes;c__Cytophagia;o__Cytophagales;f__Cytophagaceae;g__Ohtaekwangia
OTU_654	p__Bacteroidetes;c__Sphingobacteriia;o__Sphingobacteriales;f__Chitinophagaceae;
OTU_731	p__Actinobacteria;c__Actinobacteria;o__Propionibacteriales;f__Nocardioidaceae;g__Marmoricola
OTU_343	p__Proteobacteria;c__Deltaproteobacteria;o__Myxococcales
OTU_63	p__Actinobacteria;c__Actinobacteria;o__Frankiales;f__Geodermatophilaceae;
OTU_23	p__Firmicutes;c__Bacilli;o__Bacillales;f__Bacillaceae;g__Bacillus
OTU_1811	p__Proteobacteria;c__Alphaproteobacteria;o__Rhizobiales;f__Methylobacteriaceae;g__Microvirga
OTU_172	p__Proteobacteria;c__Betaproteobacteria;o__Rhodocyclales;f__Rhodocyclaceae;
OTU_2150	p__Actinobacteria;c__Actinobacteria;o__Micrococcales
OTU_246	p__Actinobacteria;c__Actinobacteria;o__Propionibacteriales;f__Nocardioidaceae;g__Nocardioides
OTU_158	p__Firmicutes;c__Bacilli;o__Bacillales
OTU_340	p__Proteobacteria;c__Gammaproteobacteria;o__Pseudomonadales;f__Moraxellaceae;
OTU_700	p__Bacteroidetes;c__Sphingobacteriia;o__Sphingobacteriales;f__Chitinophagaceae;g__Flavitalea
OTU_3001	p__Bacteroidetes;c__Sphingobacteriia;o__Sphingobacteriales;f__Chitinophagaceae;
OTU_432	p__Firmicutes;c__Bacilli;o__Bacillales;f__Paenibacillaceae;g__Brevibacillus
OTU_313	p__Proteobacteria;c__Alphaproteobacteria;o__Rhizobiales;f__Rhizobiaceae;g__Ensifer
OTU_51	p__Proteobacteria;c__Alphaproteobacteria;o__Sphingomonadales;f__Sphingomonadaceae;g__Sphingomonas
OTU_688	p__Bacteroidetes;c__Cytophagia;o__Cytophagales;f__Cytophagaceae;
OTU_92	p__Proteobacteria;c__Alphaproteobacteria;o__Caulobacterales;f__Caulobacteraceae;g__Caulobacter
OTU_381	p__Bacteroidetes;c__Sphingobacteriia;o__Sphingobacteriales;f__Chitinophagaceae;g__Niastella
OTU_91	p__Bacteroidetes;c__Sphingobacteriia;o__Sphingobacteriales;f__Chitinophagaceae;
OTU_1680	p__Firmicutes;c__Clostridia;o__Clostridiales;f__Peptostreptococcaceae;
OTU_338	p__Proteobacteria;c__Gammaproteobacteria;o__Legionellales;f__Legionellaceae;g__Legionella
OTU_972	p__Bacteroidetes;c__Sphingobacteriia;o__Sphingobacteriales;f__Chitinophagaceae;g__Terrimonas
OTU_842	p__Gemmatimonadetes;c__Gemmatimonadetes;o__Gemmatimonadales;f__Gemmatimonadaceae;
OTU_79	p__Thaumarchaeota;c__Soil Crenarchaeotic Group(SCG)
OTU_588	p__Proteobacteria;c__Alphaproteobacteria;o__Rhizobiales;f__Xanthobacteraceae;g__Variibacter
OTU_570	p__Proteobacteria;c__Alphaproteobacteria;o__Caulobacterales;f__Caulobacteraceae;g__Phenylobacterium
OTU_12	p__Thaumarchaeota;c__Soil Crenarchaeotic Group(SCG)
OTU_1215	p__Bacteroidetes;c__Sphingobacteriia;o__Sphingobacteriales;f__Chitinophagaceae;
OTU_269	p__Bacteroidetes;c__Cytophagia;o__Cytophagales;f__Cytophagaceae;g__Adhaeribacter
OTU_386	p__Bacteroidetes;c__Sphingobacteriia;o__Sphingobacteriales;f__Chitinophagaceae;
OTU_42	p__Firmicutes;c__Bacilli;o__Bacillales;f__Paenibacillaceae;g__Paenibacillus
OTU_190	p__Proteobacteria;c__Alphaproteobacteria;o__Rhizobiales;f__Phyllobacteriaceae;g__Phyllobacterium
OTU_116	p__Firmicutes;c__Bacilli;o__Bacillales;f__Paenibacillaceae;g__Paenibacillus
OTU_59	p__Actinobacteria;c__Rubrobacteria;o__Rubrobacterales;f__Rubrobacteriaceae;g__Rubrobacter
OTU_134	p__Actinobacteria;c__Actinobacteria;o__Propionibacteriales;f__Nocardioidaceae;g__Nocardioides
OTU_7	p__Thaumarchaeota;c__Soil Crenarchaeotic Group(SCG)
OTU_478	p__Proteobacteria;c__Betaproteobacteria;o__Burkholderiales;f__Comamonadaceae;g__Ramlibacter
OTU_384	p__Actinobacteria;c__Thermoleophilia;o__Solirubrobacterales;f__Solirubrobacteraceae;g__Solirubrobacter
OTU_247	p__Proteobacteria;c__Alphaproteobacteria;o__Caulobacterales;f__Caulobacteraceae;g__Phenylobacterium
OTU_348	p__Actinobacteria;c__Actinobacteria;o__Micromonosporales;f__Micromonosporaceae;
OTU_186	p__Proteobacteria;c__Alphaproteobacteria;o__Rhizobiales;f__Bradyrhizobiaceae;g__Bradyrhizobium
OTU_276	p__Proteobacteria;c__Alphaproteobacteria;o__Rhizobiales;f__Hyphomicrobiaceae;g__Devosia
OTU_227	p__Nitrospirae;c__Nitrospira;o__Nitrospirales;f__Nitrospiraceae;g__Nitrospira
OTU_104	p__Proteobacteria;c__Gammaproteobacteria;o__Cellvibrionales;f__Cellvibrionaceae;g__Cellvibrio
OTU_118	p__Actinobacteria;c__Actinobacteria;o__Micromonosporales;f__Micromonosporaceae;
OTU_480	p__Gemmatimonadetes;c__Gemmatimonadetes;o__Gemmatimonadales;f__Gemmatimonadaceae;
OTU_37	p__Firmicutes;c__Bacilli;o__Bacillales;f__Planococcaceae;g__Paenisporosarcina
OTU_410	p__Bacteroidetes;c__Cytophagia;o__Cytophagales;f__Cytophagaceae;g__Chryseolinea
OTU_46	p__Thaumarchaeota;c__Soil Crenarchaeotic Group(SCG)
OTU_539	p__Firmicutes;c__Bacilli;o__Bacillales;f__Planococcaceae;g__Sporosarcina
OTU_324	p__Firmicutes;c__Bacilli;o__Bacillales;f__Paenibacillaceae;g__Paenibacillus
OTU_113	p__Actinobacteria;c__Actinobacteria;o__Corynebacteriales;f__Nocardiaceae;g__Nocardia
OTU_287	p__Actinobacteria;c__Thermoleophilia;o__Gaiellales
OTU_686	p__Proteobacteria;c__Gammaproteobacteria;o__Xanthomonadales;f__Xanthomonadales Incertae Sedis;
OTU_979	p__Proteobacteria;c__Alphaproteobacteria;o__Rhizobiales;f__Hyphomicrobiaceae;g__Rhodoplanes
OTU_231	p__Actinobacteria;c__Acidimicrobiia;o__Acidimicrobiales;f__Acidimicrobiaceae;

OTU_334	p__Gemmatimonadetes;c__Gemmatimonadetes;o__Gemmatimonadales;f__Gemmatimonadaceae;
OTU_176	p__Actinobacteria;c__Thermoleophilia;o__Gaiellales
OTU_610	p__Bacteroidetes;c__Sphingobacteriia;o__Sphingobacteriales;f__Chitinophagaceae;
OTU_215	p__Firmicutes;c__Clostridia;o__Clostridiales;f__Lachnospiraceae;g__Mobilitalea
OTU_3454	p__Bacteroidetes;c__Sphingobacteriia;o__Sphingobacteriales;f__Chitinophagaceae;
OTU_715	p__Proteobacteria;c__Gammaproteobacteria;o__Xanthomonadales;f__Xanthomonadales Incertae Sedis;g__Acidibacter
OTU_4647	p__Proteobacteria;c__Alphaproteobacteria;o__Rhizobiales;f__Xanthobacteraceae;
OTU_71	p__Actinobacteria;c__Thermoleophilia;o__Gaiellales;f__Gaiellaceae;g__Gaiella
OTU_3722	p__Proteobacteria;c__Alphaproteobacteria;o__Rhizobiales;f__Phyllobacteriaceae;
OTU_957	p__Bacteroidetes;c__Sphingobacteriia;o__Sphingobacteriales;f__Chitinophagaceae;g__Parasegetibacter
OTU_136	p__Actinobacteria;c__Actinobacteria;o__Propionibacteriales;f__Nocardioidaceae;
OTU_2167	p__Proteobacteria;c__Alphaproteobacteria;o__Rhizobiales;f__Rhizobiales Incertae Sedis;g__Nordella
OTU_426	p__Proteobacteria;c__Betaproteobacteria;o__Burkholderiales;f__Comamonadaceae;
OTU_102	p__Proteobacteria;c__Gammaproteobacteria;o__Xanthomonadales;f__Xanthomonadaceae;
OTU_408	p__Thaumarchaeota;c__Soil Crenarchaeotic Group(SCG)
OTU_528	p__Proteobacteria;c__Alphaproteobacteria;o__Caulobacterales;f__Hyphomonadaceae;g__Hirschia
OTU_873	p__Proteobacteria;c__Alphaproteobacteria;o__Rhizobiales;f__Hyphomicrobiaceae;
OTU_250	p__Proteobacteria;c__Betaproteobacteria;o__Burkholderiales;f__Oxalobacteraceae;g__Massilia
OTU_202	p__Firmicutes;c__Bacilli;o__Bacillales;f__Paenibacillaceae;g__Paenibacillus
OTU_154	p__Actinobacteria;c__Thermoleophilia;o__Gaiellales;f__Gaiellaceae;g__Gaiella
OTU_211	p__Actinobacteria;c__Thermoleophilia;o__Solirubrobacterales;f__Elev-16S-1332;
OTU_4023	p__Proteobacteria;
OTU_416	p__Firmicutes;c__Bacilli;o__Bacillales;f__Thermoactinomycetaceae;g__Thermoactinomyces
OTU_995	p__Proteobacteria;c__Deltaproteobacteria;o__Myxococcales;f__Polyangiaceae;g__Sorangium
OTU_52	p__Actinobacteria;c__Actinobacteria;o__Propionibacteriales;f__Propionibacteriaceae;g__Microlunatus
OTU_309	p__Bacteroidetes;c__Sphingobacteriia;o__Sphingobacteriales;f__Chitinophagaceae;
OTU_329	p__Actinobacteria;c__Thermoleophilia;o__Gaiellales
OTU_616	p__Proteobacteria;c__Alphaproteobacteria;o__Rhizobiales;f__Phyllobacteriaceae;
OTU_346	p__Firmicutes;c__Clostridia;o__Clostridiales;f__Clostridiaceae 1;g__Clostridium sensu stricto 13
OTU_538	p__Actinobacteria;c__Actinobacteria;o__Micromonosporales;f__Micromonosporaceae;
OTU_81	p__Actinobacteria;c__Thermoleophilia;o__Gaiellales;f__Gaiellaceae;g__Gaiella
OTU_636	p__Firmicutes;c__Bacilli;o__Bacillales;f__Planococcaceae;
OTU_739	p__Firmicutes;c__Clostridia;o__Clostridiales;f__Clostridiaceae 1;g__Clostridium sensu stricto 12
OTU_617	p__Proteobacteria;c__Betaproteobacteria;o__Burkholderiales;f__Comamonadaceae;
OTU_218	p__Actinobacteria;c__Actinobacteria;o__Pseudonocardiales;f__Pseudonocardiaceae;
OTU_592	p__Firmicutes;c__Bacilli;o__Bacillales
OTU_236	p__Proteobacteria;c__Alphaproteobacteria;o__Sphingomonadales;f__Erythrobacteraceae;g__Altererythrobacter
OTU_1086	p__Proteobacteria;c__Alphaproteobacteria;o__Rhizobiales;f__JG34-KF-361;
OTU_333	p__Actinobacteria;c__Thermoleophilia;o__Solirubrobacterales;f__319-6M6;
OTU_268	p__Proteobacteria;c__Gammaproteobacteria;o__Xanthomonadales;f__Xanthomonadales Incertae Sedis;g__Acidibacter
OTU_289	p__Proteobacteria;c__Betaproteobacteria;o__Burkholderiales;f__Comamonadaceae;
OTU_1093	p__Actinobacteria;c__Acidimicrobiia;o__Acidimicrobiales;f__Iamiaceae;g__Iamia
OTU_259	p__Actinobacteria;c__Actinobacteria;o__Propionibacteriales;f__Nocardioidaceae;
OTU_3809	p__Thaumarchaeota;c__Soil Crenarchaeotic Group(SCG)
OTU_133	p__Bacteroidetes;c__Sphingobacteriia;o__Sphingobacteriales;f__Chitinophagaceae;g__Terrimonas
OTU_691	p__Bacteroidetes;c__Sphingobacteriia;o__Sphingobacteriales;f__Chitinophagaceae;
OTU_295	p__Actinobacteria;c__Actinobacteria;o__Propionibacteriales;f__Nocardioidaceae;g__Nocardioides
OTU_3481	p__Firmicutes;c__Bacilli;o__Bacillales;f__Paenibacillaceae;g__Paenibacillus
OTU_819	p__Proteobacteria;c__Alphaproteobacteria;o__Rhodospirillales;f__Rhodospirillaceae;g__Skermanella
OTU_414	p__Firmicutes;c__Clostridia;o__Clostridiales;f__Peptostreptococcaceae;g__Sporacetigenium
OTU_651	p__Proteobacteria;c__Alphaproteobacteria;o__Rhizobiales;f__MNG7;
OTU_924	p__Proteobacteria;c__Alphaproteobacteria;o__Rhodospirillales;f__Rhodospirillaceae;
OTU_1298	p__Actinobacteria;c__Thermoleophilia;o__Gaiellales
OTU_418	p__Actinobacteria;c__Acidimicrobiia;o__Acidimicrobiales;f__Acidimicrobiaceae;g__Ilumatobacter
OTU_1046	p__Proteobacteria;c__Alphaproteobacteria;o__Rhizobiales;f__Xanthobacteraceae;g__Variibacter
OTU_565	p__Proteobacteria;c__Alphaproteobacteria;o__Rhodospirillales;f__Rhodospirillales Incertae Sedis;g__Reyranella
OTU_524	p__Proteobacteria;c__Deltaproteobacteria;o__Bdellovibrionales;f__Bacteriovoracaceae;g__Peredibacter
OTU_328	p__Actinobacteria;c__Actinobacteria;o__Pseudonocardiales;f__Pseudonocardiaceae;g__Pseudonocardia
OTU_522	p__Proteobacteria;c__Deltaproteobacteria;o__Myxococcales;f__Sandaracinaceae;
OTU_188	p__Proteobacteria;c__Gammaproteobacteria;o__Xanthomonadales;f__Xanthomonadales Incertae Sedis;
OTU_22441	p__Actinobacteria;c__Actinobacteria;o__Corynebacteriales;f__Mycobacteriaceae;g__Mycobacterium
OTU_22371	p__Actinobacteria;c__Actinobacteria;o__Micrococcales;f__Microbacteriaceae;g__Microbacterium
OTU_406	p__Firmicutes;c__Bacilli;o__Bacillales;f__Paenibacillaceae;g__Cohnella
OTU_1305	p__Proteobacteria;c__Gammaproteobacteria;o__Xanthomonadales;f__Xanthomonadales Incertae Sedis;
OTU_258	p__Firmicutes;c__Bacilli;o__Bacillales;f__Thermoactinomycetaceae;g__Planifilum

	OTU_749	p__Proteobacteria;c__Alphaproteobacteria;o__Rhizobiales;f__Rhizobiales Incertae Sedis;g__Nordella
	OTU_345	p__Bacteroidetes;c__Cytophagia;o__Cytophagales;f__Cytophagaceae;g__Adhaeribacter
	OTU_377	p__Proteobacteria;c__Gammaproteobacteria;o__Xanthomonadales
	OTU_373	p__Acidobacteria;c__Holophagae;o__Subgroup 1;f__ABS-19;
	OTU_1409	p__Proteobacteria;c__Alphaproteobacteria;o__Sphingomonadales;f__Ellin655;
	OTU_785	p__Actinobacteria;c__Acidimicrobiia;o__Acidimicrobiales;f__Iamiaceae;g__Iamia
	OTU_560	p__Proteobacteria;c__Alphaproteobacteria;o__Rhodospirillales;f__Rhodospirillaceae;g__Ferrovibrio
	OTU_1722	p__Proteobacteria;c__Betaproteobacteria;o__Burkholderiales;f__Comamonadaceae;g__Ramlibacter
	OTU_318	p__Actinobacteria;c__Actinobacteria;o__Corynebacteriales;f__Mycobacteriaceae;g__Mycobacterium
	OTU_306	p__Proteobacteria;c__Alphaproteobacteria;o__Sphingomonadales;f__Sphingomonadaceae;g__Sphingomonas
	OTU_344	p__Proteobacteria;c__Alphaproteobacteria;o__Sphingomonadales;f__Sphingomonadaceae;g__Sphingomonas
	OTU_941	p__Proteobacteria;c__Alphaproteobacteria;o__Rhizobiales;f__Methylobacteriaceae;
	OTU_1304	p__Actinobacteria;c__Actinobacteria;o__Micromonosporales;f__Micromonosporaceae;
	OTU_529	p__Actinobacteria;c__Thermoleophilia;o__Solirubrobacterales;f__Elev-16S-1332;
	OTU_375	p__Actinobacteria;c__Actinobacteria;o__Micrococcales;f__Microbacteriaceae;
	OTU_380	p__Bacteroidetes;c__Cytophagia;o__Cytophagales;f__Cytophagaceae;
	OTU_500	p__Proteobacteria;c__Gammaproteobacteria;o__Xanthomonadales;f__Xanthomonadaceae;
	OTU_393	p__Actinobacteria;c__Acidimicrobiia;o__Acidimicrobiales
	OTU_14606	p__Proteobacteria;c__Betaproteobacteria;o__Burkholderiales;f__Comamonadaceae;
	OTU_579	p__Firmicutes;c__Clostridia;o__Clostridiales;f__Clostridiaceae 1;g__Clostridium sensu stricto 13
	OTU_322	p__Bacteroidetes;c__Cytophagia;o__Cytophagales;f__Cytophagaceae;g__Ohtaekwangia
	OTU_244	p__Firmicutes;c__Bacilli;o__Bacillales;f__Paenibacillaceae;g__Cohnella
	OTU_58092	p__Actinobacteria;c__Actinobacteria;o__Corynebacteriales;f__Mycobacteriaceae;g__Mycobacterium
	OTU_1395	p__Proteobacteria;c__Alphaproteobacteria;o__Rhizobiales;f__Hyphomicrobiaceae;g__Pedomicrobium
	OTU_502	p__Proteobacteria;c__Alphaproteobacteria;o__Rhizobiales;f__Xanthobacteraceae;g__Variibacter
	OTU_257	p__Actinobacteria;c__Thermoleophilia;o__Gaiellales
	OTU_1534	p__Thaumarchaeota;c__Soil Crenarchaeotic Group(SCG)
	OTU_112	p__Thaumarchaeota;c__Soil Crenarchaeotic Group(SCG);o__Unknown Order;f__Unknown Family;g__Candidatus Nitrososphaera
	OTU_890	p__Actinobacteria;c__Acidimicrobiia;o__Acidimicrobiales;f__Acidimicrobiaceae;
	OTU_78	p__Bacteroidetes;c__Cytophagia;o__Cytophagales;f__Cytophagaceae;
	OTU_466	p__Proteobacteria;c__Alphaproteobacteria;o__Rhizobiales;f__Rhodobiaceae;
	OTU_7832	p__Bacteroidetes;c__Sphingobacteriia;o__Sphingobacteriales;f__Chitinophagaceae;
	OTU_5804	p__Proteobacteria;c__Gammaproteobacteria;o__Xanthomonadales;f__Xanthomonadales Incertae Sedis;
	OTU_2701	p__Proteobacteria;c__Betaproteobacteria;o__Nitrosomonadales;f__Nitrosomonadaceae;
	OTU_877	p__Actinobacteria;c__Thermoleophilia;o__Gaiellales
	OTU_302	p__Actinobacteria;c__Acidimicrobiia;o__Acidimicrobiales;f__OM1 clade;
	OTU_3618	p__Proteobacteria;c__Alphaproteobacteria;o__Rhodospirillales;f__Rhodospirillaceae;
	OTU_207	p__Proteobacteria;c__Deltaproteobacteria;o__Myxococcales;f__Sandaracinaceae;
	OTU_620	p__Bacteroidetes;c__Cytophagia;o__Cytophagales;f__Cytophagaceae;g__Ohtaekwangia
	OTU_461	p__Proteobacteria;c__Alphaproteobacteria;o__Rhizobiales;f__Xanthobacteraceae;
	OTU_512	p__Proteobacteria;c__Betaproteobacteria;o__Burkholderiales;f__Comamonadaceae;g__Aquabacterium
	OTU_1621	p__Actinobacteria;c__Acidimicrobiia;o__Acidimicrobiales;f__Acidimicrobiaceae;
	OTU_239	p__Bacteroidetes;c__Sphingobacteriia;o__Sphingobacteriales;f__Chitinophagaceae;g__Lacibacter
	OTU_667	p__Bacteroidetes;c__Sphingobacteriia;o__Sphingobacteriales;f__Chitinophagaceae;g__Flavitalea
	OTU_681	p__Proteobacteria;c__Alphaproteobacteria;o__Rhizobiales;f__Hyphomicrobiaceae;g__Hyphomicrobium
	OTU_1009	p__Proteobacteria;c__Gammaproteobacteria;o__Xanthomonadales;f__Xanthomonadaceae;
	OTU_261	p__Proteobacteria;c__Betaproteobacteria;o__Nitrosomonadales;f__Nitrosomonadaceae;
	OTU_1363	p__Actinobacteria;c__Acidimicrobiia;o__Acidimicrobiales
	OTU_143	p__Actinobacteria;c__Thermoleophilia;o__Gaiellales
	OTU_520	p__Proteobacteria;c__Betaproteobacteria;o__SC-I-84
	OTU_465	p__Acidobacteria;c__Holophagae;o__Subgroup 1;f__ABS-19;
	OTU_5772	p__Proteobacteria;c__Deltaproteobacteria;o__Desulfurellales;f__Desulfurellaceae;g__H16
	OTU_503	p__Proteobacteria;c__Gammaproteobacteria;o__Xanthomonadales
	OTU_145	p__Actinobacteria;c__Thermoleophilia;o__Solirubrobacterales;f__Q3-6C1;
	OTU_1117	p__Proteobacteria;c__Alphaproteobacteria;o__Rhizobiales;f__Hyphomicrobiaceae;g__Pedomicrobium
	OTU_1478	p__Proteobacteria;c__Betaproteobacteria;o__Burkholderiales;f__Comamonadaceae;g__Aquabacterium
	OTU_563	p__Proteobacteria;c__Alphaproteobacteria;o__Rhizobiales;f__Rhodobiaceae;
	OTU_379	p__Tectomicrobia;
	OTU_2047	p__Actinobacteria;c__Thermoleophilia;o__Gaiellales
	OTU_279	p__Actinobacteria;
	OTU_447	p__Proteobacteria;c__Alphaproteobacteria;o__Rhodospirillales;f__Rhodospirillaceae;g__Skermanella
	OTU_1256	p__Actinobacteria;c__Rubrobacteria;o__Rubrobacterales;f__Rubrobacteriaceae;g__Rubrobacter
	OTU_299	p__Bacteroidetes;c__Cytophagia;o__Cytophagales;f__Cytophagaceae;g__Pontibacter
	OTU_448	p__Actinobacteria;c__Thermoleophilia;o__Gaiellales
	OTU_741	p__Actinobacteria;c__Actinobacteria;o__Propionibacteriales;f__Nocardioidaceae;g__Nocardioides
	OTU_360	p__Actinobacteria;c__Acidimicrobiia;o__Acidimicrobiales;f__OM1 clade;

	OTU_725	p__Bacteroidetes;c__Cytophagia;o__Cytophagales;f__Cytophagaceae;
	OTU_537	p__Bacteroidetes;c__Cytophagia;o__Cytophagales;f__Cytophagaceae;
	OTU_1033	p__Actinobacteria;c__Thermoleophilia;o__Solirubrobacterales
	OTU_594	p__Actinobacteria;c__Thermoleophilia;o__Gaiellales
	OTU_413	p__Actinobacteria;c__Acidimicrobiia;o__Acidimicrobiales;f__OM1 clade;
	OTU_382	p__Actinobacteria;c__Thermoleophilia;o__Gaiellales
	OTU_724	p__Proteobacteria;c__Betaproteobacteria;o__Nitrosomonadales;f__Nitrosomonadaceae;
	OTU_1521	p__Actinobacteria;c__Thermoleophilia;o__Solirubrobacterales
	OTU_719	p__Chloroflexi;c__S85
	OTU_130	p__Actinobacteria;c__MB-A2-18
	OTU_1230	p__Acidobacteria;c__Subgroup 11
	OTU_536	p__Thaumarchaeota;c__Soil Crenarchaeotic Group(SCG)
	OTU_1360	p__Proteobacteria;c__Alphaproteobacteria;o__Rhizobiales;f__Methylobacteriaceae;g__Microvirga
	OTU_573	p__Bacteroidetes;c__Sphingobacteriia;o__Sphingobacteriales;f__Saprospiraceae;
	OTU_1333	p__Gemmatimonadetes;c__Gemmatimonadetes;o__Gemmatimonadales;f__Gemmatimonadaceae;
	OTU_604	p__Bacteroidetes;c__Sphingobacteriia;o__Sphingobacteriales;f__Chitinophagaceae;
	OTU_1194	p__Proteobacteria;c__Alphaproteobacteria;o__Rhizobiales;f__Neo-b11;
	OTU_1024	p__Proteobacteria;c__Betaproteobacteria;o__TRA3-2
	OTU_362	p__Proteobacteria;c__Betaproteobacteria;o__Burkholderiales;f__Alcaligenaceae;
	OTU_321	p__Tectomicrobia
	OTU_293	p__Bacteroidetes;c__Cytophagia;o__Cytophagales;f__Cytophagaceae;
	OTU_1143	p__Actinobacteria;c__Actinobacteria;o__Micrococcales;f__Intrasporangiaceae;g__Intrasporangium
	OTU_3580	p__Gemmatimonadetes;c__Gemmatimonadetes;o__Gemmatimonadales;f__Gemmatimonadaceae;
	OTU_193	p__Actinobacteria;c__MB-A2-18
	OTU_635	p__Proteobacteria;c__Alphaproteobacteria;o__Rhizobiales;f__Rhizobiales Incertae Sedis;g__Nordella
	OTU_843	p__Actinobacteria;c__TakashiAC-B11
	OTU_5766	p__Actinobacteria;c__Thermoleophilia;o__Gaiellales
	OTU_2160	p__Proteobacteria;c__Deltaproteobacteria;o__Myxococcales;f__Polyangiaceae;
	OTU_1421	p__Proteobacteria;c__Betaproteobacteria;o__SC-I-84
	OTU_2060	p__Proteobacteria;c__Alphaproteobacteria;o__Rhizobiales;f__DUNssu44;
	OTU_513	p__Chloroflexi;c__Chloroflexia;o__Chloroflexales;f__Roseiflexaceae;g__Roseiflexus
T0|T1	OTU_20	p__Bacteroidetes;c__Flavobacteriia;o__Flavobacteriales;f__Flavobacteriaceae;g__Chryseobacterium
	OTU_100	p__Proteobacteria;c__Alphaproteobacteria;o__Caulobacterales;f__Caulobacteraceae;g__Caulobacter
T0|T2 Tulln	OTU_18	p__Firmicutes;c__Bacilli;o__Bacillales
	OTU_54	p__Firmicutes;c__Bacilli;o__Bacillales;f__Bacillaceae;g__Bacillus
T1|T2 Tulln	OTU_27	p__Actinobacteria;c__Actinobacteria;o__Streptomycetales;f__Streptomycetaceae;g__Streptomyces
	OTU_9	p__Actinobacteria;c__Actinobacteria;o__Micrococcales;f__Microbacteriaceae;g__Microbacterium
	OTU_56	p__Actinobacteria;c__Actinobacteria;o__Micrococcales;f__Micrococcaceae;g__Arthrobacter
	OTU_45	p__Proteobacteria;c__Gammaproteobacteria;o__Pseudomonadales;f__Pseudomonadaceae;g__Pseudomonas
	OTU_50	p__Proteobacteria;c__Betaproteobacteria;o__Methylophilales;f__Methylophilaceae;
	OTU_67	p__Actinobacteria;c__Actinobacteria;o__Pseudonocardiales;f__Pseudonocardiaceae;
	OTU_68	p__Actinobacteria;c__Actinobacteria;o__Micrococcales;f__Microbacteriaceae;
	OTU_1252	p__Proteobacteria;c__Alphaproteobacteria;o__Rhizobiales;f__Hyphomicrobiaceae;g__Devosia
	OTU_44	p__Bacteroidetes;c__Sphingobacteriia;o__Sphingobacteriales;f__Chitinophagaceae;g__Niastella
	OTU_88	p__Proteobacteria;c__Alphaproteobacteria;o__Rhizobiales;f__Phyllobacteriaceae;g__Mesorhizobium
	OTU_238	p__Proteobacteria;c__Alphaproteobacteria;o__Rhizobiales;f__Bradyrhizobiaceae;g__Bosea
	OTU_212	p__Proteobacteria;c__Betaproteobacteria;o__Burkholderiales;f__Comamonadaceae;g__Hydrogenophaga
	OTU_83	p__Proteobacteria;c__Alphaproteobacteria;o__Rhizobiales;f__Hyphomicrobiaceae;g__Devosia
	OTU_147	p__Proteobacteria;c__Alphaproteobacteria;o__Rhizobiales;f__Bradyrhizobiaceae;
	OTU_392	p__Proteobacteria;c__Alphaproteobacteria;o__Sphingomonadales;f__Sphingomonadaceae;g__Sphingobium
	OTU_242	p__Proteobacteria;c__Alphaproteobacteria;o__Rhizobiales;f__Hyphomicrobiaceae;g__Devosia
	OTU_195	p__Proteobacteria;c__Alphaproteobacteria;o__Rhodospirillales;f__Rhodospirillaceae;g__Dongia
	OTU_165	p__Proteobacteria;c__Gammaproteobacteria;o__Pseudomonadales;f__Moraxellaceae;
	OTU_235	p__Proteobacteria;c__Gammaproteobacteria;o__Xanthomonadales;f__Xanthomonadaceae;g__Pseudoxanthomonas
	OTU_64	p__Proteobacteria;c__Alphaproteobacteria;o__Rhizobiales;f__Rhizobiaceae;g__Shinella
	OTU_641	p__Proteobacteria;c__Alphaproteobacteria;o__Rhizobiales;f__Phyllobacteriaceae;
	OTU_148	p__Proteobacteria;c__Alphaproteobacteria;o__Rhizobiales;f__Hyphomicrobiaceae;g__Devosia
	OTU_501	p__Actinobacteria;c__Acidimicrobiia;o__Acidimicrobiales;f__Acidimicrobiaceae;
	OTU_527	p__Proteobacteria;c__Alphaproteobacteria;o__Sphingomonadales;f__Erythrobacteraceae;g__Altererythrobacter
	OTU_85	p__Proteobacteria;c__Alphaproteobacteria;o__Sphingomonadales;f__Sphingomonadaceae;g__Sphingopyxis
	OTU_223	p__Actinobacteria;c__Acidimicrobiia;o__Acidimicrobiales;f__Iamiaceae;g__Iamia
	OTU_179	p__Proteobacteria;c__Alphaproteobacteria;o__Rhizobiales;f__Phyllobacteriaceae;
	OTU_69	p__Actinobacteria;c__Actinobacteria;o__Propionibacteriales;f__Nocardioidaceae;g__Nocardioides
	OTU_141	p__Actinobacteria;c__Thermoleophilia;o__Solirubrobacterales;f__Gsoil-1167;
	OTU_4010	p__Proteobacteria;c__Alphaproteobacteria;o__Rhizobiales;f__Hyphomicrobiaceae;g__Devosia
	OTU_174	p__Actinobacteria;c__Actinobacteria;o__Corynebacteriales;f__Mycobacteriaceae;g__Mycobacterium
	OTU_1288	p__Proteobacteria;c__Alphaproteobacteria;o__Rhizobiales;f__Rhizobiales Incertae Sedis;g__Bauldia

	OTU_15	p__Proteobacteria;c__Gammaproteobacteria;o__Pseudomonadales;f__Pseudomonadaceae;g__Pseudomonas
T0|T1|T2 Tulln	OTU_97	p__Actinobacteria;c__Actinobacteria;o__Propionibacteriales;f__Nocardioidaceae;
	OTU_3	p__Proteobacteria;c__Alphaproteobacteria;o__Rhizobiales;f__Rhizobiaceae;g__Rhizobium
	OTU_8	p__Actinobacteria;c__Actinobacteria;o__Micrococcales;f__Micrococcaceae;g__Pseudarthrobacter
	OTU_1	p__Firmicutes;c__Bacilli;o__Bacillales;f__Bacillaceae;g__Bacillus
	OTU_22	p__Proteobacteria;c__Betaproteobacteria;o__Burkholderiales;f__Comamonadaceae;g__Variovorax
	OTU_19	p__Proteobacteria;c__Alphaproteobacteria;o__Rhizobiales;f__Phyllobacteriaceae;g__Mesorhizobium

		**Kettlasbrunn A**
T0	OTU_4	p__Firmicutes;c__Bacilli;o__Bacillales;f__Staphylococcaceae;g__Staphylococcus
	OTU_13	p__Proteobacteria;c__Gammaproteobacteria;o__Pseudomonadales;f__Moraxellaceae;g__Acinetobacter
T1	OTU_58	p__Actinobacteria;c__Actinobacteria;o__Micrococcales;f__Microbacteriaceae;
	OTU_109	p__Actinobacteria;c__Actinobacteria;o__Propionibacteriales;f__Propionibacteriaceae;g__Propionibacterium
	OTU_35	p__Proteobacteria;c__Alphaproteobacteria;o__Caulobacterales;f__Caulobacteraceae;g__Asticcacaulis
	OTU_43	p__Bacteroidetes;c__Cytophagia;o__Cytophagales;f__Cytophagaceae;
	OTU_16	p__Bacteroidetes;c__Cytophagia;o__Cytophagales;f__Cytophagaceae;g__Emticicia
	OTU_468	p__Proteobacteria
	OTU_90	p__Bacteroidetes;c__Flavobacteriia;o__Flavobacteriales;f__Flavobacteriaceae;g__Chryseobacterium
	OTU_45	p__Proteobacteria;c__Gammaproteobacteria;o__Pseudomonadales;f__Pseudomonadaceae;g__Pseudomonas
	OTU_68	p__Actinobacteria;c__Actinobacteria;o__Micrococcales;f__Microbacteriaceae;
	OTU_464	p__Actinobacteria;c__Acidimicrobiia;o__Acidimicrobiales
	OTU_755	p__Actinobacteria;c__Acidimicrobiia;o__Acidimicrobiales
	OTU_131	p__Actinobacteria;c__Actinobacteria;o__Streptosporangiales;f__Thermomonosporaceae;
	OTU_155	p__Bacteroidetes;c__Sphingobacteriia;o__Sphingobacteriales;f__Chitinophagaceae;g__Chitinophaga
	OTU_119	p__Proteobacteria;c__Alphaproteobacteria;o__Rhizobiales;f__Rhizobiaceae;g__Shinella
	OTU_319	p__Actinobacteria;c__Acidimicrobiia;o__Acidimicrobiales;f__Iamiaceae;g__Iamia
	OTU_350	p__Proteobacteria;c__Alphaproteobacteria;o__Rhizobiales;f__Xanthobacteraceae;g__Pseudolabrys
	OTU_88	p__Proteobacteria;c__Alphaproteobacteria;o__Rhizobiales;f__Phyllobacteriaceae;g__Mesorhizobium
	OTU_5	p__Actinobacteria;c__Actinobacteria;o__Micrococcales;f__Cellulomonadaceae;g__Cellulomonas
	OTU_364	p__Proteobacteria;c__Alphaproteobacteria;o__Caulobacterales;f__Caulobacteraceae;g__Brevundimonas
	OTU_876	p__Actinobacteria;c__Actinobacteria;o__Micrococcales;f__Microbacteriaceae;g__Leifsonia
	OTU_392	p__Proteobacteria;c__Alphaproteobacteria;o__Sphingomonadales;f__Sphingomonadaceae;g__Sphingobium
	OTU_195	p__Proteobacteria;c__Alphaproteobacteria;o__Rhodospirillales;f__Rhodospirillaceae;g__Dongia
	OTU_509	p__Actinobacteria;c__Thermoleophilia;o__Solirubrobacterales;f__Elev-16S-1332;
	OTU_140	p__Proteobacteria;c__Alphaproteobacteria;o__Caulobacterales;f__Caulobacteraceae;g__Brevundimonas
	OTU_518	p__Proteobacteria;c__Alphaproteobacteria;o__Rhizobiales;f__Rhizobiales Incertae Sedis;g__Bauldia
	OTU_230	p__Actinobacteria;c__Acidimicrobiia;o__Acidimicrobiales;f__Iamiaceae;g__Iamia
	OTU_178	p__Actinobacteria;c__Actinobacteria;o__Propionibacteriales;f__Nocardioidaceae;g__Kribbella
	OTU_2846	p__Actinobacteria;c__Actinobacteria;o__Micrococcales;f__Microbacteriaceae;g__Microbacterium
	OTU_75	p__Bacteroidetes;c__Sphingobacteriia;o__Sphingobacteriales;f__Chitinophagaceae;g__Chitinophaga
	OTU_108	p__Bacteroidetes;c__Sphingobacteriia;o__Sphingobacteriales;f__Chitinophagaceae;g__Terrimonas
	OTU_505	p__Proteobacteria;c__Alphaproteobacteria;o__Rhodospirillales;f__Rhodospirillales Incertae Sedis;g__Reyranella
	OTU_501	p__Actinobacteria;c__Acidimicrobiia;o__Acidimicrobiales;f__Acidimicrobiaceae;
	OTU_527	p__Proteobacteria;c__Alphaproteobacteria;o__Sphingomonadales;f__Erythrobacteraceae;g__Altererythrobacter
	OTU_85	p__Proteobacteria;c__Alphaproteobacteria;o__Sphingomonadales;f__Sphingomonadaceae;g__Sphingopyxis
	OTU_601	p__Proteobacteria;c__Betaproteobacteria;o__Burkholderiales;f__Comamonadaceae;
	OTU_618	p__Proteobacteria;c__Alphaproteobacteria;o__Rhizobiales;f__Methylobacteriaceae;
	OTU_179	p__Proteobacteria;c__Alphaproteobacteria;o__Rhizobiales;f__Phyllobacteriaceae;
	OTU_252	p__Proteobacteria;c__Alphaproteobacteria;o__Rhizobiales;f__Brucellaceae;g__Ochrobactrum
	OTU_1094	p__Proteobacteria;c__Gammaproteobacteria;o__Xanthomonadales;f__Xanthomonadaceae;g__Dokdonella
	OTU_4010	p__Proteobacteria;c__Alphaproteobacteria;o__Rhizobiales;f__Hyphomicrobiaceae;g__Devosia
	OTU_1288	p__Proteobacteria;c__Alphaproteobacteria;o__Rhizobiales;f__Rhizobiales Incertae Sedis;g__Bauldia
	OTU_1267	p__Proteobacteria;c__Alphaproteobacteria;o__Rhizobiales
	OTU_1600	p__Proteobacteria;c__Alphaproteobacteria;o__Rhizobiales;f__Hyphomicrobiaceae;
	OTU_357	p__Proteobacteria;c__Alphaproteobacteria;o__Rhizobiales;f__Rhizobiales Incertae Sedis;g__Rhizomicrobium
T2-KA	OTU_33	p__Proteobacteria;c__Betaproteobacteria;o__Burkholderiales;f__Comamonadaceae;
	OTU_39	p__Bacteroidetes;c__Sphingobacteriia;o__Sphingobacteriales;f__Chitinophagaceae;g__Terrimonas
	OTU_30	p__Saccharibacteria
	OTU_28	p__Actinobacteria;c__Actinobacteria;o__Micrococcales;f__Microbacteriaceae;g__Agromyces
	OTU_34	p__Actinobacteria;c__Actinobacteria;o__Micrococcales;f__Micrococcaceae;
	OTU_142	p__Proteobacteria;c__Alphaproteobacteria;o__Caulobacterales;f__Caulobacteraceae;
	OTU_160	p__Bacteroidetes;c__Sphingobacteriia;o__Sphingobacteriales;f__Sphingobacteriaceae;
	OTU_1461	p__Actinobacteria;c__Actinobacteria;o__Streptomycetales;f__Streptomycetaceae;g__Streptomyces
	OTU_425	p__Actinobacteria;c__Actinobacteria;o__Streptomycetales;f__Streptomycetaceae;g__Streptomyces
	OTU_95	p__Actinobacteria;c__Actinobacteria;o__Streptomycetales;f__Streptomycetaceae;g__Streptomyces
	OTU_74	p__Proteobacteria;c__Betaproteobacteria;o__Burkholderiales;f__Comamonadaceae;

OTU_25	p__Actinobacteria;c__Actinobacteria;o__Micrococcales;f__Micrococcaceae;
OTU_282	p__Chloroflexi;c__S85
OTU_630	p__Actinobacteria;c__Actinobacteria;o__Propionibacteriales;f__Nocardioidaceae;g__Nocardioides
OTU_221	p__Proteobacteria;c__Alphaproteobacteria;o__Rhizobiales;f__Rhizobiaceae;
OTU_225	p__Proteobacteria;c__Betaproteobacteria;o__Burkholderiales;f__Oxalobacteraceae;g__Paucimonas
OTU_773	p__Proteobacteria;c__Betaproteobacteria;o__Burkholderiales;f__Comamonadaceae;
OTU_297	p__Proteobacteria;c__Betaproteobacteria;o__Burkholderiales;f__Oxalobacteraceae;
OTU_280	p__Actinobacteria;c__Actinobacteria;o__Frankiales;f__Geodermatophilaceae;g__Blastococcus
OTU_2313	p__Actinobacteria;c__Actinobacteria;o__Propionibacteriales;f__Nocardioidaceae;g__Marmoricola
OTU_135	p__Firmicutes;c__Bacilli;o__Bacillales;f__Paenibacillaceae;g__Paenibacillus
OTU_105	p__Proteobacteria;c__Gammaproteobacteria;o__Xanthomonadales;f__Xanthomonadaceae;g__Dyella
OTU_266	p__Bacteroidetes;c__Sphingobacteriia;o__Sphingobacteriales;f__Chitinophagaceae;
OTU_827	p__Actinobacteria;c__Actinobacteria;o__Propionibacteriales;f__Nocardioidaceae;g__Nocardioides
OTU_111	p__Bacteroidetes;c__Cytophagia;o__Cytophagales;f__Cytophagaceae;g__Ohtaekwangia
OTU_731	p__Actinobacteria;c__Actinobacteria;o__Propionibacteriales;f__Nocardioidaceae;g__Marmoricola
OTU_63	p__Actinobacteria;c__Actinobacteria;o__Frankiales;f__Geodermatophilaceae;
OTU_23	p__Firmicutes;c__Bacilli;o__Bacillales;f__Bacillaceae;g__Bacillus
OTU_1811	p__Proteobacteria;c__Alphaproteobacteria;o__Rhizobiales;f__Methylobacteriaceae;g__Microvirga
OTU_1559	p__Actinobacteria;c__Actinobacteria;o__Streptomycetales;f__Streptomycetaceae;g__Streptomyces
OTU_172	p__Proteobacteria;c__Betaproteobacteria;o__Rhodocyclales;f__Rhodocyclaceae;
OTU_2150	p__Actinobacteria;c__Actinobacteria;o__Micrococcales
OTU_246	p__Actinobacteria;c__Actinobacteria;o__Propionibacteriales;f__Nocardioidaceae;g__Nocardioides
OTU_158	p__Firmicutes;c__Bacilli;o__Bacillales
OTU_581	p__Bacteroidetes;c__Cytophagia;o__Cytophagales;f__Cytophagaceae;g__Ohtaekwangia
OTU_340	p__Proteobacteria;c__Gammaproteobacteria;o__Pseudomonadales;f__Moraxellaceae;
OTU_700	p__Bacteroidetes;c__Sphingobacteriia;o__Sphingobacteriales;f__Chitinophagaceae;g__Flavitalea
OTU_3612	p__Actinobacteria;c__Actinobacteria;o__Corynebacteriales;f__Mycobacteriaceae;g__Mycobacterium
OTU_313	p__Proteobacteria;c__Alphaproteobacteria;o__Rhizobiales;f__Rhizobiaceae;g__Ensifer
OTU_51	p__Proteobacteria;c__Alphaproteobacteria;o__Sphingomonadales;f__Sphingomonadaceae;g__Sphingomonas
OTU_92	p__Proteobacteria;c__Alphaproteobacteria;o__Caulobacterales;f__Caulobacteraceae;g__Caulobacter
OTU_336	p__Armatimonadetes;c__Fimbriimonadia;o__Fimbriimonadales;f__Fimbriimonadaceae;
OTU_91	p__Bacteroidetes;c__Sphingobacteriia;o__Sphingobacteriales;f__Chitinophagaceae;
OTU_291	p__Bacteroidetes;c__Cytophagia;o__Cytophagales;f__Cytophagaceae;g__Cytophaga
OTU_338	p__Proteobacteria;c__Gammaproteobacteria;o__Legionellales;f__Legionellaceae;g__Legionella
OTU_428	p__Firmicutes;c__Bacilli;o__Bacillales;f__Bacillaceae;g__Bacillus
OTU_842	p__Gemmatimonadetes;c__Gemmatimonadetes;o__Gemmatimonadales;f__Gemmatimonadaceae;
OTU_79	p__Thaumarchaeota;c__Soil Crenarchaeotic Group(SCG)
OTU_570	p__Proteobacteria;c__Alphaproteobacteria;o__Caulobacterales;f__Caulobacteraceae;g__Phenylobacterium
OTU_128	p__Bacteroidetes;c__Sphingobacteriia;o__Sphingobacteriales;f__Sphingobacteriaceae;
OTU_12	p__Thaumarchaeota;c__Soil Crenarchaeotic Group(SCG)
OTU_1215	p__Bacteroidetes;c__Sphingobacteriia;o__Sphingobacteriales;f__Chitinophagaceae;
OTU_269	p__Bacteroidetes;c__Cytophagia;o__Cytophagales;f__Cytophagaceae;g__Adhaeribacter
OTU_386	p__Bacteroidetes;c__Sphingobacteriia;o__Sphingobacteriales;f__Chitinophagaceae;
OTU_370	p__Bacteroidetes;c__Cytophagia;o__Cytophagales;f__Cytophagaceae;
OTU_42	p__Firmicutes;c__Bacilli;o__Bacillales;f__Paenibacillaceae;g__Paenibacillus
OTU_190	p__Proteobacteria;c__Alphaproteobacteria;o__Rhizobiales;f__Phyllobacteriaceae;g__Phyllobacterium
OTU_116	p__Firmicutes;c__Bacilli;o__Bacillales;f__Paenibacillaceae;g__Paenibacillus
OTU_59	p__Actinobacteria;c__Rubrobacteria;o__Rubrobacterales;f__Rubrobacteriaceae;g__Rubrobacter
OTU_134	p__Actinobacteria;c__Actinobacteria;o__Propionibacteriales;f__Nocardioidaceae;g__Nocardioides
OTU_7	p__Thaumarchaeota;c__Soil Crenarchaeotic Group(SCG)
OTU_296	p__Proteobacteria;c__Betaproteobacteria;o__Nitrosomonadales;f__Nitrosomonadaceae;
OTU_348	p__Actinobacteria;c__Actinobacteria;o__Micromonosporales;f__Micromonosporaceae;
OTU_186	p__Proteobacteria;c__Alphaproteobacteria;o__Rhizobiales;f__Bradyrhizobiaceae;g__Bradyrhizobium
OTU_276	p__Proteobacteria;c__Alphaproteobacteria;o__Rhizobiales;f__Hyphomicrobiaceae;g__Devosia
OTU_227	p__Nitrospirae;c__Nitrospira;o__Nitrospirales;f__Nitrospiraceae;g__Nitrospira
OTU_200	p__Bacteroidetes;c__Sphingobacteriia;o__Sphingobacteriales;f__Chitinophagaceae;g__Ferruginibacter
OTU_118	p__Actinobacteria;c__Actinobacteria;o__Micromonosporales;f__Micromonosporaceae;
OTU_480	p__Gemmatimonadetes;c__Gemmatimonadetes;o__Gemmatimonadales;f__Gemmatimonadaceae;
OTU_37	p__Firmicutes;c__Bacilli;o__Bacillales;f__Planococcaceae;g__Paenisporosarcina
OTU_352	p__Proteobacteria;c__Betaproteobacteria;o__Burkholderiales;f__Oxalobacteraceae;
OTU_46	p__Thaumarchaeota;c__Soil Crenarchaeotic Group(SCG)
OTU_539	p__Firmicutes;c__Bacilli;o__Bacillales;f__Planococcaceae;g__Sporosarcina
OTU_123	p__Actinobacteria;c__Actinobacteria;o__Propionibacteriales;f__Nocardioidaceae;g__Nocardioides
OTU_113	p__Actinobacteria;c__Actinobacteria;o__Corynebacteriales;f__Nocardiaceae;g__Nocardia
OTU_287	p__Actinobacteria;c__Thermoleophilia;o__Gaiellales
OTU_686	p__Proteobacteria;c__Gammaproteobacteria;o__Xanthomonadales;f__Xanthomonadales Incertae Sedis;
OTU_231	p__Actinobacteria;c__Acidimicrobiia;o__Acidimicrobiales;f__Acidimicrobiaceae;

OTU_334	p__Gemmatimonadetes;c__Gemmatimonadetes;o__Gemmatimonadales;f__Gemmatimonadaceae;
OTU_176	p__Actinobacteria;c__Thermoleophilia;o__Gaiellales
OTU_215	p__Firmicutes;c__Clostridia;o__Clostridiales;f__Lachnospiraceae;g__Mobilitalea
OTU_389	p__Proteobacteria;c__Betaproteobacteria;o__Burkholderiales;f__Comamonadaceae;g__Polaromonas
OTU_15516	p__Actinobacteria;c__Actinobacteria;o__Propionibacteriales;f__Nocardioidaceae;g__Nocardioides
OTU_3454	p__Bacteroidetes;c__Sphingobacteriia;o__Sphingobacteriales;f__Chitinophagaceae;
OTU_71	p__Actinobacteria;c__Thermoleophilia;o__Gaiellales;f__Gaiellaceae;g__Gaiella
OTU_3722	p__Proteobacteria;c__Alphaproteobacteria;o__Rhizobiales;f__Phyllobacteriaceae;
OTU_136	p__Actinobacteria;c__Actinobacteria;o__Propionibacteriales;f__Nocardioidaceae;
OTU_426	p__Proteobacteria;c__Betaproteobacteria;o__Burkholderiales;f__Comamonadaceae;
OTU_102	p__Proteobacteria;c__Gammaproteobacteria;o__Xanthomonadales;f__Xanthomonadaceae;
OTU_304	p__Firmicutes;c__Bacilli;o__Bacillales;f__Bacillaceae;g__Bacillus
OTU_408	p__Thaumarchaeota;c__Soil Crenarchaeotic Group(SCG)
OTU_873	p__Proteobacteria;c__Alphaproteobacteria;o__Rhizobiales;f__Hyphomicrobiaceae;
OTU_213	p__Proteobacteria;c__Betaproteobacteria;o__Methylophilales;f__Methylophilaceae;g__Methylotenera
OTU_250	p__Proteobacteria;c__Betaproteobacteria;o__Burkholderiales;f__Oxalobacteraceae;g__Massilia
OTU_202	p__Firmicutes;c__Bacilli;o__Bacillales;f__Paenibacillaceae;g__Paenibacillus
OTU_154	p__Actinobacteria;c__Thermoleophilia;o__Gaiellales;f__Gaiellaceae;g__Gaiella
OTU_211	p__Actinobacteria;c__Thermoleophilia;o__Solirubrobacterales;f__Elev-16S-1332;
OTU_995	p__Proteobacteria;c__Deltaproteobacteria;o__Myxococcales;f__Polyangiaceae;g__Sorangium
OTU_52	p__Actinobacteria;c__Actinobacteria;o__Propionibacteriales;f__Propionibacteriaceae;g__Microlunatus
OTU_309	p__Bacteroidetes;c__Sphingobacteriia;o__Sphingobacteriales;f__Chitinophagaceae;
OTU_329	p__Actinobacteria;c__Thermoleophilia;o__Gaiellales
OTU_1771	p__Firmicutes;c__Bacilli;o__Bacillales;f__Bacillaceae;g__Bacillus
OTU_538	p__Actinobacteria;c__Actinobacteria;o__Micromonosporales;f__Micromonosporaceae;
OTU_81	p__Actinobacteria;c__Thermoleophilia;o__Gaiellales;f__Gaiellaceae;g__Gaiella
OTU_636	p__Firmicutes;c__Bacilli;o__Bacillales;f__Planococcaceae;
OTU_617	p__Proteobacteria;c__Betaproteobacteria;o__Burkholderiales;f__Comamonadaceae;
OTU_218	p__Actinobacteria;c__Actinobacteria;o__Pseudonocardiales;f__Pseudonocardiaceae;
OTU_592	p__Firmicutes;c__Bacilli;o__Bacillales
OTU_236	p__Proteobacteria;c__Alphaproteobacteria;o__Sphingomonadales;f__Erythrobacteraceae;g__Altererythrobacter
OTU_349	p__Proteobacteria;c__Betaproteobacteria;o__Burkholderiales;f__Comamonadaceae;
OTU_333	p__Actinobacteria;c__Thermoleophilia;o__Solirubrobacterales;f__319-6M6;
OTU_268	p__Proteobacteria;c__Gammaproteobacteria;o__Xanthomonadales;f__Xanthomonadales Incertae Sedis;g__Acidibacter
OTU_3809	p__Thaumarchaeota;c__Soil Crenarchaeotic Group(SCG)
OTU_133	p__Bacteroidetes;c__Sphingobacteriia;o__Sphingobacteriales;f__Chitinophagaceae;g__Terrimonas
OTU_691	p__Bacteroidetes;c__Sphingobacteriia;o__Sphingobacteriales;f__Chitinophagaceae;
OTU_2737	p__Firmicutes;c__Bacilli;o__Bacillales;f__Planococcaceae;
OTU_295	p__Actinobacteria;c__Actinobacteria;o__Propionibacteriales;f__Nocardioidaceae;g__Nocardioides
OTU_400	p__Actinobacteria;c__Actinobacteria;o__Frankiales;f__Sporichthyaceae;
OTU_414	p__Firmicutes;c__Clostridia;o__Clostridiales;f__Peptostreptococcaceae;g__Sporacetigenium
OTU_603	p__Firmicutes;c__Bacilli;o__Bacillales;f__Paenibacillaceae;g__Paenibacillus
OTU_418	p__Actinobacteria;c__Acidimicrobiia;o__Acidimicrobiales;f__Acidimicrobiaceae;g__Ilumatobacter
OTU_1046	p__Proteobacteria;c__Alphaproteobacteria;o__Rhizobiales;f__Xanthobacteraceae;g__Variibacter
OTU_565	p__Proteobacteria;c__Alphaproteobacteria;o__Rhodospirillales;f__Rhodospirillales Incertae Sedis;g__Reyranella
OTU_1200	p__Actinobacteria;c__Acidimicrobiia;o__Acidimicrobiales;f__Iamiaceae;g__Iamia
OTU_524	p__Proteobacteria;c__Deltaproteobacteria;o__Bdellovibrionales;f__Bacteriovoracaceae;g__Peredibacter
OTU_328	p__Actinobacteria;c__Actinobacteria;o__Pseudonocardiales;f__Pseudonocardiaceae;g__Pseudonocardia
OTU_188	p__Proteobacteria;c__Gammaproteobacteria;o__Xanthomonadales;f__Xanthomonadales Incertae Sedis;
OTU_22441	p__Actinobacteria;c__Actinobacteria;o__Corynebacteriales;f__Mycobacteriaceae;g__Mycobacterium
OTU_439	p__Actinobacteria;c__Actinobacteria;o__Propionibacteriales;f__Nocardioidaceae;g__Nocardioides
OTU_22371	p__Actinobacteria;c__Actinobacteria;o__Micrococcales;f__Microbacteriaceae;g__Microbacterium
OTU_1659	p__Proteobacteria;c__Deltaproteobacteria;o__Bdellovibrionales;f__Bacteriovoracaceae;g__Peredibacter
OTU_345	p__Bacteroidetes;c__Cytophagia;o__Cytophagales;f__Cytophagaceae;g__Adhaeribacter
OTU_377	p__Proteobacteria;c__Gammaproteobacteria;o__Xanthomonadales
OTU_373	p__Acidobacteria;c__Holophagae;o__Subgroup 1;f__ABS-19;
OTU_2917	p__Actinobacteria;c__Actinobacteria;o__Frankiales;f__Geodermatophilaceae;
OTU_883	p__Proteobacteria;c__Alphaproteobacteria;o__Sphingomonadales;f__Ellin655;
OTU_785	p__Actinobacteria;c__Acidimicrobiia;o__Acidimicrobiales;f__Iamiaceae;g__Iamia
OTU_560	p__Proteobacteria;c__Alphaproteobacteria;o__Rhodospirillales;f__Rhodospirillaceae;g__Ferrovibrio
OTU_9738	p__Actinobacteria;c__Actinobacteria;o__Streptosporangiales;f__Streptosporangiaceae;
OTU_344	p__Proteobacteria;c__Alphaproteobacteria;o__Sphingomonadales;f__Sphingomonadaceae;g__Sphingomonas
OTU_529	p__Actinobacteria;c__Thermoleophilia;o__Solirubrobacterales;f__Elev-16S-1332;
OTU_380	p__Bacteroidetes;c__Cytophagia;o__Cytophagales;f__Cytophagaceae;
OTU_500	p__Proteobacteria;c__Gammaproteobacteria;o__Xanthomonadales;f__Xanthomonadaceae;
OTU_393	p__Actinobacteria;c__Acidimicrobiia;o__Acidimicrobiales

OTU_14606	p__Proteobacteria;c__Betaproteobacteria;o__Burkholderiales;f__Comamonadaceae;
OTU_244	p__Firmicutes;c__Bacilli;o__Bacillales;f__Paenibacillaceae;g__Cohnella
OTU_502	p__Proteobacteria;c__Alphaproteobacteria;o__Rhizobiales;f__Xanthobacteraceae;g__Variibacter
OTU_4963	p__Proteobacteria;c__Alphaproteobacteria;o__Sphingomonadales;f__Sphingomonadaceae;g__Sphingomonas
OTU_776	p__Proteobacteria;c__Alphaproteobacteria;o__Rhizobiales
OTU_257	p__Actinobacteria;c__Thermoleophilia;o__Gaiellales
OTU_531	p__Actinobacteria;c__Actinobacteria;o__Micrococcales;f__Intrasporangiaceae;
OTU_473	p__Nitrospirae;c__Nitrospira;o__Nitrospirales;f__Nitrospiraceae;g__Nitrospira
OTU_1534	p__Thaumarchaeota;c__Soil Crenarchaeotic Group(SCG)
OTU_112	p__Thaumarchaeota;c__Soil Crenarchaeotic Group(SCG);o__Unknown Order;f__Unknown Family;g__Candidatus Nitrososphaera
OTU_78	p__Bacteroidetes;c__Cytophagia;o__Cytophagales;f__Cytophagaceae;
OTU_466	p__Proteobacteria;c__Alphaproteobacteria;o__Rhizobiales;f__Rhodobiaceae;
OTU_19054	p__Bacteroidetes;c__Sphingobacteriia;o__Sphingobacteriales;f__Sphingobacteriaceae;
OTU_5804	p__Proteobacteria;c__Gammaproteobacteria;o__Xanthomonadales;f__Xanthomonadales Incertae Sedis;
OTU_2701	p__Proteobacteria;c__Betaproteobacteria;o__Nitrosomonadales;f__Nitrosomonadaceae;
OTU_302	p__Actinobacteria;c__Acidimicrobiia;o__Acidimicrobiales;f__OM1 clade;
OTU_672	p__Actinobacteria;c__Actinobacteria;o__Streptosporangiales;f__Streptosporangiaceae;
OTU_3618	p__Proteobacteria;c__Alphaproteobacteria;o__Rhodospirillales;f__Rhodospirillaceae;
OTU_620	p__Bacteroidetes;c__Cytophagia;o__Cytophagales;f__Cytophagaceae;g__Ohtaekwangia
OTU_398	p__Actinobacteria;c__Actinobacteria;o__Frankiales
OTU_461	p__Proteobacteria;c__Alphaproteobacteria;o__Rhizobiales;f__Xanthobacteraceae;
OTU_1621	p__Actinobacteria;c__Acidimicrobiia;o__Acidimicrobiales;f__Acidimicrobiaceae;
OTU_239	p__Bacteroidetes;c__Sphingobacteriia;o__Sphingobacteriales;f__Chitinophagaceae;g__Lacibacter
OTU_681	p__Proteobacteria;c__Alphaproteobacteria;o__Rhizobiales;f__Hyphomicrobiaceae;g__Hyphomicrobium
OTU_1009	p__Proteobacteria;c__Gammaproteobacteria;o__Xanthomonadales;f__Xanthomonadaceae;
OTU_261	p__Proteobacteria;c__Betaproteobacteria;o__Nitrosomonadales;f__Nitrosomonadaceae;
OTU_143	p__Actinobacteria;c__Thermoleophilia;o__Gaiellales
OTU_465	p__Acidobacteria;c__Holophagae;o__Subgroup 1;f__ABS-19;
OTU_5772	p__Proteobacteria;c__Deltaproteobacteria;o__Desulfurellales;f__Desulfurellaceae;g__H16
OTU_978	p__Actinobacteria;c__Acidimicrobiia;o__Acidimicrobiales
OTU_503	p__Proteobacteria;c__Gammaproteobacteria;o__Xanthomonadales
OTU_145	p__Actinobacteria;c__Thermoleophilia;o__Solirubrobacterales;f__Q3-6C1;
OTU_379	p__Tectomicrobia
OTU_279	p__Actinobacteria
OTU_680	p__Proteobacteria;c__Alphaproteobacteria;o__Rhizobiales
OTU_1256	p__Actinobacteria;c__Rubrobacteria;o__Rubrobacterales;f__Rubrobacteriaceae;g__Rubrobacter
OTU_299	p__Bacteroidetes;c__Cytophagia;o__Cytophagales;f__Cytophagaceae;g__Pontibacter
OTU_448	p__Actinobacteria;c__Thermoleophilia;o__Gaiellales
OTU_741	p__Actinobacteria;c__Actinobacteria;o__Propionibacteriales;f__Nocardioidaceae;g__Nocardioides
OTU_360	p__Actinobacteria;c__Acidimicrobiia;o__Acidimicrobiales;f__OM1 clade;
OTU_725	p__Bacteroidetes;c__Cytophagia;o__Cytophagales;f__Cytophagaceae;
OTU_537	p__Bacteroidetes;c__Cytophagia;o__Cytophagales;f__Cytophagaceae;
OTU_1033	p__Actinobacteria;c__Thermoleophilia;o__Solirubrobacterales
OTU_553	p__Acidobacteria;c__Subgroup 5
OTU_594	p__Actinobacteria;c__Thermoleophilia;o__Gaiellales
OTU_413	p__Actinobacteria;c__Acidimicrobiia;o__Acidimicrobiales;f__OM1 clade;
OTU_382	p__Actinobacteria;c__Thermoleophilia;o__Gaiellales
OTU_724	p__Proteobacteria;c__Betaproteobacteria;o__Nitrosomonadales;f__Nitrosomonadaceae;
OTU_1521	p__Actinobacteria;c__Thermoleophilia;o__Solirubrobacterales
OTU_719	p__Chloroflexi;c__S85
OTU_130	p__Actinobacteria;c__MB-A2-18
OTU_536	p__Thaumarchaeota;c__Soil Crenarchaeotic Group(SCG)
OTU_1360	p__Proteobacteria;c__Alphaproteobacteria;o__Rhizobiales;f__Methylobacteriaceae;g__Microvirga
OTU_573	p__Bacteroidetes;c__Sphingobacteriia;o__Sphingobacteriales;f__Saprospiraceae;
OTU_1275	p__Actinobacteria;c__Acidimicrobiia;o__Acidimicrobiales
OTU_1278	p__Proteobacteria;c__Deltaproteobacteria;o__Myxococcales;f__BIrii41;
OTU_604	p__Bacteroidetes;c__Sphingobacteriia;o__Sphingobacteriales;f__Chitinophagaceae;
OTU_1000	p__Proteobacteria;c__Deltaproteobacteria;o__Myxococcales;f__Nannocystaceae;g__Nannocystis
OTU_1156	p__Thaumarchaeota;c__Soil Crenarchaeotic Group(SCG)
OTU_362	p__Proteobacteria;c__Betaproteobacteria;o__Burkholderiales;f__Alcaligenaceae;
OTU_832	p__Actinobacteria;c__MB-A2-18
OTU_3580	p__Gemmatimonadetes;c__Gemmatimonadetes;o__Gemmatimonadales;f__Gemmatimonadaceae;
OTU_193	p__Actinobacteria;c__MB-A2-18
OTU_1767	p__Actinobacteria;c__Thermoleophilia;o__Gaiellales
OTU_843	p__Actinobacteria;c__TakashiAC-B11
OTU_782	p__Proteobacteria;c__Alphaproteobacteria;o__Rhizobiales;f__Methylobacteriaceae;g__Microvirga

	OTU_962	p__Proteobacteria;c__Gammaproteobacteria;o__Xanthomonadales;f__Xanthomonadaceae;g__Arenimonas
	OTU_273	p__Bacteroidetes;c__Cytophagia;o__Cytophagales;f__Cytophagaceae;
	OTU_1419	p__Actinobacteria;c__Actinobacteria;o__Pseudonocardiales;f__Pseudonocardiaceae;g__Pseudonocardia
	OTU_896	p__Bacteroidetes
	OTU_1425	p__Actinobacteria;c__Actinobacteria;o__Streptomycetales;f__Streptomycetaceae;g__Streptomyces
T0|T1	OTU_20	p__Bacteroidetes;c__Flavobacteriia;o__Flavobacteriales;f__Flavobacteriaceae;g__Chryseobacterium
	OTU_15	p__Proteobacteria;c__Gammaproteobacteria;o__Pseudomonadales;f__Pseudomonadaceae;g__Pseudomonas
	OTU_19	p__Proteobacteria;c__Alphaproteobacteria;o__Rhizobiales;f__Phyllobacteriaceae;g__Mesorhizobium
	OTU_100	p__Proteobacteria;c__Alphaproteobacteria;o__Caulobacterales;f__Caulobacteraceae;g__Caulobacter
T0|T2-KA	OTU_18	p__Firmicutes;c__Bacilli;o__Bacillales
	OTU_54	p__Firmicutes;c__Bacilli;o__Bacillales;f__Bacillaceae;g__Bacillus
T1|T2-KA	OTU_27	p__Actinobacteria;c__Actinobacteria;o__Streptomycetales;f__Streptomycetaceae;g__Streptomyces
	OTU_9	p__Actinobacteria;c__Actinobacteria;o__Micrococcales;f__Microbacteriaceae;g__Microbacterium
	OTU_56	p__Actinobacteria;c__Actinobacteria;o__Micrococcales;f__Micrococcaceae;g__Arthrobacter
	OTU_17	p__Saccharibacteria
	OTU_10	p__Proteobacteria;c__Betaproteobacteria;o__Burkholderiales;f__Burkholderiaceae;g__Ralstonia
	OTU_50	p__Proteobacteria;c__Betaproteobacteria;o__Methylophilales;f__Methylophilaceae;
	OTU_67	p__Actinobacteria;c__Actinobacteria;o__Pseudonocardiales;f__Pseudonocardiaceae;
	OTU_1252	p__Proteobacteria;c__Alphaproteobacteria;o__Rhizobiales;f__Hyphomicrobiaceae;g__Devosia
	OTU_44	p__Bacteroidetes;c__Sphingobacteriia;o__Sphingobacteriales;f__Chitinophagaceae;g__Niastella
	OTU_65	p__Proteobacteria;c__Alphaproteobacteria;o__Rhizobiales;f__Rhizobiaceae;g__Rhizobium
	OTU_238	p__Proteobacteria;c__Alphaproteobacteria;o__Rhizobiales;f__Bradyrhizobiaceae;g__Bosea
	OTU_212	p__Proteobacteria;c__Betaproteobacteria;o__Burkholderiales;f__Comamonadaceae;g__Hydrogenophaga
	OTU_83	p__Proteobacteria;c__Alphaproteobacteria;o__Rhizobiales;f__Hyphomicrobiaceae;g__Devosia
	OTU_147	p__Proteobacteria;c__Alphaproteobacteria;o__Rhizobiales;f__Bradyrhizobiaceae;
	OTU_242	p__Proteobacteria;c__Alphaproteobacteria;o__Rhizobiales;f__Hyphomicrobiaceae;g__Devosia
	OTU_165	p__Proteobacteria;c__Gammaproteobacteria;o__Pseudomonadales;f__Moraxellaceae;
	OTU_235	p__Proteobacteria;c__Gammaproteobacteria;o__Xanthomonadales;f__Xanthomonadaceae;g__Pseudoxanthomonas
	OTU_194	p__Proteobacteria;c__Alphaproteobacteria;o__Sphingomonadales;f__Sphingomonadaceae;g__Novosphingobium
	OTU_64	p__Proteobacteria;c__Alphaproteobacteria;o__Rhizobiales;f__Rhizobiaceae;g__Shinella
	OTU_641	p__Proteobacteria;c__Alphaproteobacteria;o__Rhizobiales;f__Phyllobacteriaceae;
	OTU_148	p__Proteobacteria;c__Alphaproteobacteria;o__Rhizobiales;f__Hyphomicrobiaceae;g__Devosia
	OTU_223	p__Actinobacteria;c__Acidimicrobiia;o__Acidimicrobiales;f__Iamiaceae;g__Iamia
	OTU_69	p__Actinobacteria;c__Actinobacteria;o__Propionibacteriales;f__Nocardioidaceae;g__Nocardioides
	OTU_141	p__Actinobacteria;c__Thermoleophilia;o__Solirubrobacterales;f__Gsoil-1167;
	OTU_174	p__Actinobacteria;c__Actinobacteria;o__Corynebacteriales;f__Mycobacteriaceae;g__Mycobacterium
T0|T1|T2-KA	OTU_97	p__Actinobacteria;c__Actinobacteria;o__Propionibacteriales;f__Nocardioidaceae;
	OTU_3	p__Proteobacteria;c__Alphaproteobacteria;o__Rhizobiales;f__Rhizobiaceae;g__Rhizobium
	OTU_8	p__Actinobacteria;c__Actinobacteria;o__Micrococcales;f__Micrococcaceae;g__Pseudarthrobacter
	OTU_1	p__Firmicutes;c__Bacilli;o__Bacillales;f__Bacillaceae;g__Bacillus
	OTU_22	p__Proteobacteria;c__Betaproteobacteria;o__Burkholderiales;f__Comamonadaceae;g__Variovorax

	**Kettlasbrunn B**	
T0	OTU_4	p__Firmicutes;c__Bacilli;o__Bacillales;f__Staphylococcaceae;g__Staphylococcus
	OTU_13	p__Proteobacteria;c__Gammaproteobacteria;o__Pseudomonadales;f__Moraxellaceae;g__Acinetobacter
T1	OTU_58	p__Actinobacteria;c__Actinobacteria;o__Micrococcales;f__Microbacteriaceae;
	OTU_109	p__Actinobacteria;c__Actinobacteria;o__Propionibacteriales;f__Propionibacteriaceae;g__Propionibacterium
	OTU_17	p__Saccharibacteria;
	OTU_43	p__Bacteroidetes;c__Cytophagia;o__Cytophagales;f__Cytophagaceae;
	OTU_16	p__Bacteroidetes;c__Cytophagia;o__Cytophagales;f__Cytophagaceae;g__Emticicia
	OTU_90	p__Bacteroidetes;c__Flavobacteriia;o__Flavobacteriales;f__Flavobacteriaceae;g__Chryseobacterium
	OTU_45	p__Proteobacteria;c__Gammaproteobacteria;o__Pseudomonadales;f__Pseudomonadaceae;g__Pseudomonas
	OTU_464	p__Actinobacteria;c__Acidimicrobiia;o__Acidimicrobiales
	OTU_755	p__Actinobacteria;c__Acidimicrobiia;o__Acidimicrobiales
	OTU_131	p__Actinobacteria;c__Actinobacteria;o__Streptosporangiales;f__Thermomonosporaceae;
	OTU_155	p__Bacteroidetes;c__Sphingobacteriia;o__Sphingobacteriales;f__Chitinophagaceae;g__Chitinophaga
	OTU_119	p__Proteobacteria;c__Alphaproteobacteria;o__Rhizobiales;f__Rhizobiaceae;g__Shinella
	OTU_319	p__Actinobacteria;c__Acidimicrobiia;o__Acidimicrobiales;f__Iamiaceae;g__Iamia
	OTU_350	p__Proteobacteria;c__Alphaproteobacteria;o__Rhizobiales;f__Xanthobacteraceae;g__Pseudolabrys
	OTU_5	p__Actinobacteria;c__Actinobacteria;o__Micrococcales;f__Cellulomonadaceae;g__Cellulomonas
	OTU_364	p__Proteobacteria;c__Alphaproteobacteria;o__Caulobacterales;f__Caulobacteraceae;g__Brevundimonas
	OTU_238	p__Proteobacteria;c__Alphaproteobacteria;o__Rhizobiales;f__Bradyrhizobiaceae;g__Bosea
	OTU_876	p__Actinobacteria;c__Actinobacteria;o__Micrococcales;f__Microbacteriaceae;g__Leifsonia
	OTU_392	p__Proteobacteria;c__Alphaproteobacteria;o__Sphingomonadales;f__Sphingomonadaceae;g__Sphingobium
	OTU_242	p__Proteobacteria;c__Alphaproteobacteria;o__Rhizobiales;f__Hyphomicrobiaceae;g__Devosia
	OTU_195	p__Proteobacteria;c__Alphaproteobacteria;o__Rhodospirillales;f__Rhodospirillaceae;g__Dongia
	OTU_235	p__Proteobacteria;c__Gammaproteobacteria;o__Xanthomonadales;f__Xanthomonadaceae;g__Pseudoxanthomonas

	OTU_509	p__Actinobacteria;c__Thermoleophilia;o__Solirubrobacterales;f__Elev-16S-1332;
	OTU_140	p__Proteobacteria;c__Alphaproteobacteria;o__Caulobacterales;f__Caulobacteraceae;g__Brevundimonas
	OTU_518	p__Proteobacteria;c__Alphaproteobacteria;o__Rhizobiales;f__Rhizobiales Incertae Sedis;g__Bauldia
	OTU_230	p__Actinobacteria;c__Acidimicrobiia;o__Acidimicrobiales;f__Iamiaceae;g__Iamia
	OTU_178	p__Actinobacteria;c__Actinobacteria;o__Propionibacteriales;f__Nocardioidaceae;g__Kribbella
	OTU_2846	p__Actinobacteria;c__Actinobacteria;o__Micrococcales;f__Microbacteriaceae;g__Microbacterium
	OTU_75	p__Bacteroidetes;c__Sphingobacteriia;o__Sphingobacteriales;f__Chitinophagaceae;g__Chitinophaga
	OTU_108	p__Bacteroidetes;c__Sphingobacteriia;o__Sphingobacteriales;f__Chitinophagaceae;g__Terrimonas
	OTU_505	p__Proteobacteria;c__Alphaproteobacteria;o__Rhodospirillales;f__Rhodospirillales Incertae Sedis;g__Reyranella
	OTU_527	p__Proteobacteria;c__Alphaproteobacteria;o__Sphingomonadales;f__Erythrobacteraceae;g__Altererythrobacter
	OTU_601	p__Proteobacteria;c__Betaproteobacteria;o__Burkholderiales;f__Comamonadaceae;
	OTU_618	p__Proteobacteria;c__Alphaproteobacteria;o__Rhizobiales;f__Methylobacteriaceae;
	OTU_252	p__Proteobacteria;c__Alphaproteobacteria;o__Rhizobiales;f__Brucellaceae;g__Ochrobactrum
	OTU_1094	p__Proteobacteria;c__Gammaproteobacteria;o__Xanthomonadales;f__Xanthomonadaceae;g__Dokdonella
	OTU_4010	p__Proteobacteria;c__Alphaproteobacteria;o__Rhizobiales;f__Hyphomicrobiaceae;g__Devosia
	OTU_1288	p__Proteobacteria;c__Alphaproteobacteria;o__Rhizobiales;f__Rhizobiales Incertae Sedis;g__Bauldia
	OTU_1267	p__Proteobacteria;c__Alphaproteobacteria;o__Rhizobiales
	OTU_1600	p__Proteobacteria;c__Alphaproteobacteria;o__Rhizobiales;f__Hyphomicrobiaceae;
	OTU_357	p__Proteobacteria;c__Alphaproteobacteria;o__Rhizobiales;f__Rhizobiales Incertae Sedis;g__Rhizomicrobium
T2-KB	OTU_33	p__Proteobacteria;c__Betaproteobacteria;o__Burkholderiales;f__Comamonadaceae;
	OTU_39	p__Bacteroidetes;c__Sphingobacteriia;o__Sphingobacteriales;f__Chitinophagaceae;g__Terrimonas
	OTU_30	p__Saccharibacteria
	OTU_28	p__Actinobacteria;c__Actinobacteria;o__Micrococcales;f__Microbacteriaceae;g__Agromyces
	OTU_34	p__Actinobacteria;c__Actinobacteria;o__Micrococcales;f__Micrococcaceae;
	OTU_142	p__Proteobacteria;c__Alphaproteobacteria;o__Caulobacterales;f__Caulobacteraceae;
	OTU_160	p__Bacteroidetes;c__Sphingobacteriia;o__Sphingobacteriales;f__Sphingobacteriaceae;
	OTU_1461	p__Actinobacteria;c__Actinobacteria;o__Streptomycetales;f__Streptomycetaceae;g__Streptomyces
	OTU_95	p__Actinobacteria;c__Actinobacteria;o__Streptomycetales;f__Streptomycetaceae;g__Streptomyces
	OTU_74	p__Proteobacteria;c__Betaproteobacteria;o__Burkholderiales;f__Comamonadaceae;
	OTU_25	p__Actinobacteria;c__Actinobacteria;o__Micrococcales;f__Micrococcaceae;
	OTU_48	p__Firmicutes;c__Bacilli;o__Bacillales;f__Planococcaceae;g__Lysinibacillus
	OTU_282	p__Chloroflexi;c__S85
	OTU_630	p__Actinobacteria;c__Actinobacteria;o__Propionibacteriales;f__Nocardioidaceae;g__Nocardioides
	OTU_221	p__Proteobacteria;c__Alphaproteobacteria;o__Rhizobiales;f__Rhizobiaceae;
	OTU_407	p__Proteobacteria;c__Betaproteobacteria;o__Burkholderiales;f__Oxalobacteraceae;
	OTU_225	p__Proteobacteria;c__Betaproteobacteria;o__Burkholderiales;f__Oxalobacteraceae;g__Paucimonas
	OTU_773	p__Proteobacteria;c__Betaproteobacteria;o__Burkholderiales;f__Comamonadaceae;
	OTU_297	p__Proteobacteria;c__Betaproteobacteria;o__Burkholderiales;f__Oxalobacteraceae;
	OTU_596	p__Bacteroidetes;c__Cytophagia;o__Cytophagales;f__Cytophagaceae;g__Ohtaekwangia
	OTU_138	p__Actinobacteria;c__Actinobacteria;o__Micrococcales;f__Microbacteriaceae;
	OTU_280	p__Actinobacteria;c__Actinobacteria;o__Frankiales;f__Geodermatophilaceae;g__Blastococcus
	OTU_53	p__Proteobacteria;c__Alphaproteobacteria;o__Caulobacterales;f__Caulobacteraceae;g__Caulobacter
	OTU_135	p__Firmicutes;c__Bacilli;o__Bacillales;f__Paenibacillaceae;g__Paenibacillus
	OTU_1606	p__Proteobacteria;c__Betaproteobacteria;o__Burkholderiales;f__Comamonadaceae;
	OTU_266	p__Bacteroidetes;c__Sphingobacteriia;o__Sphingobacteriales;f__Chitinophagaceae;
	OTU_111	p__Bacteroidetes;c__Cytophagia;o__Cytophagales;f__Cytophagaceae;g__Ohtaekwangia
	OTU_654	p__Bacteroidetes;c__Sphingobacteriia;o__Sphingobacteriales;f__Chitinophagaceae;
	OTU_343	p__Proteobacteria;c__Deltaproteobacteria;o__Myxococcales
	OTU_63	p__Actinobacteria;c__Actinobacteria;o__Frankiales;f__Geodermatophilaceae;
	OTU_23	p__Firmicutes;c__Bacilli;o__Bacillales;f__Bacillaceae;g__Bacillus
	OTU_1811	p__Proteobacteria;c__Alphaproteobacteria;o__Rhizobiales;f__Methylobacteriaceae;g__Microvirga
	OTU_172	p__Proteobacteria;c__Betaproteobacteria;o__Rhodocyclales;f__Rhodocyclaceae;
	OTU_2150	p__Actinobacteria;c__Actinobacteria;o__Micrococcales
	OTU_246	p__Actinobacteria;c__Actinobacteria;o__Propionibacteriales;f__Nocardioidaceae;g__Nocardioides
	OTU_158	p__Firmicutes;c__Bacilli;o__Bacillales
	OTU_581	p__Bacteroidetes;c__Cytophagia;o__Cytophagales;f__Cytophagaceae;g__Ohtaekwangia
	OTU_340	p__Proteobacteria;c__Gammaproteobacteria;o__Pseudomonadales;f__Moraxellaceae;
	OTU_700	p__Bacteroidetes;c__Sphingobacteriia;o__Sphingobacteriales;f__Chitinophagaceae;g__Flavitalea
	OTU_313	p__Proteobacteria;c__Alphaproteobacteria;o__Rhizobiales;f__Rhizobiaceae;g__Ensifer
	OTU_51	p__Proteobacteria;c__Alphaproteobacteria;o__Sphingomonadales;f__Sphingomonadaceae;g__Sphingomonas
	OTU_92	p__Proteobacteria;c__Alphaproteobacteria;o__Caulobacterales;f__Caulobacteraceae;g__Caulobacter
	OTU_381	p__Bacteroidetes;c__Sphingobacteriia;o__Sphingobacteriales;f__Chitinophagaceae;g__Niastella
	OTU_91	p__Bacteroidetes;c__Sphingobacteriia;o__Sphingobacteriales;f__Chitinophagaceae;
	OTU_291	p__Bacteroidetes;c__Cytophagia;o__Cytophagales;f__Cytophagaceae;g__Cytophaga
	OTU_6535	p__Firmicutes;c__Bacilli;o__Bacillales;f__Planococcaceae;g__Lysinibacillus
	OTU_428	p__Firmicutes;c__Bacilli;o__Bacillales;f__Bacillaceae;g__Bacillus
	OTU_972	p__Bacteroidetes;c__Sphingobacteriia;o__Sphingobacteriales;f__Chitinophagaceae;g__Terrimonas

OTU_79	p__Thaumarchaeota;c__Soil Crenarchaeotic Group(SCG)
OTU_570	p__Proteobacteria;c__Alphaproteobacteria;o__Caulobacterales;f__Caulobacteraceae;g__Phenylobacterium
OTU_128	p__Bacteroidetes;c__Sphingobacteriia;o__Sphingobacteriales;f__Sphingobacteriaceae;
OTU_12	p__Thaumarchaeota;c__Soil Crenarchaeotic Group(SCG)
OTU_1215	p__Bacteroidetes;c__Sphingobacteriia;o__Sphingobacteriales;f__Chitinophagaceae;
OTU_269	p__Bacteroidetes;c__Cytophagia;o__Cytophagales;f__Cytophagaceae;g__Adhaeribacter
OTU_370	p__Bacteroidetes;c__Cytophagia;o__Cytophagales;f__Cytophagaceae;
OTU_42	p__Firmicutes;c__Bacilli;o__Bacillales;f__Paenibacillaceae;g__Paenibacillus
OTU_190	p__Proteobacteria;c__Alphaproteobacteria;o__Rhizobiales;f__Phyllobacteriaceae;g__Phyllobacterium
OTU_116	p__Firmicutes;c__Bacilli;o__Bacillales;f__Paenibacillaceae;g__Paenibacillus
OTU_59	p__Actinobacteria;c__Rubrobacteria;o__Rubrobacterales;f__Rubrobacteriaceae;g__Rubrobacter
OTU_134	p__Actinobacteria;c__Actinobacteria;o__Propionibacteriales;f__Nocardioidaceae;g__Nocardioides
OTU_7	p__Thaumarchaeota;c__Soil Crenarchaeotic Group(SCG)
OTU_384	p__Actinobacteria;c__Thermoleophilia;o__Solirubrobacterales;f__Solirubrobacteraceae;g__Solirubrobacter
OTU_348	p__Actinobacteria;c__Actinobacteria;o__Micromonosporales;f__Micromonosporaceae;
OTU_186	p__Proteobacteria;c__Alphaproteobacteria;o__Rhizobiales;f__Bradyrhizobiaceae;g__Bradyrhizobium
OTU_276	p__Proteobacteria;c__Alphaproteobacteria;o__Rhizobiales;f__Hyphomicrobiaceae;g__Devosia
OTU_224	p__Firmicutes;c__Bacilli;o__Bacillales;f__Bacillaceae;
OTU_227	p__Nitrospirae;c__Nitrospira;o__Nitrospirales;f__Nitrospiraceae;g__Nitrospira
OTU_104	p__Proteobacteria;c__Gammaproteobacteria;o__Cellvibrionales;f__Cellvibrionaceae;g__Cellvibrio
OTU_118	p__Actinobacteria;c__Actinobacteria;o__Micromonosporales;f__Micromonosporaceae;
OTU_37	p__Firmicutes;c__Bacilli;o__Bacillales;f__Planococcaceae;g__Paenisporosarcina
OTU_410	p__Bacteroidetes;c__Cytophagia;o__Cytophagales;f__Cytophagaceae;g__Chryseolinea
OTU_352	p__Proteobacteria;c__Betaproteobacteria;o__Burkholderiales;f__Oxalobacteraceae;
OTU_46	p__Thaumarchaeota;c__Soil Crenarchaeotic Group(SCG)
OTU_539	p__Firmicutes;c__Bacilli;o__Bacillales;f__Planococcaceae;g__Sporosarcina
OTU_686	p__Proteobacteria;c__Gammaproteobacteria;o__Xanthomonadales;f__Xanthomonadales Incertae Sedis;
OTU_312	p__Bacteroidetes;c__Sphingobacteriia;o__Sphingobacteriales;f__Chitinophagaceae;g__Parafilimonas
OTU_231	p__Actinobacteria;c__Acidimicrobiia;o__Acidimicrobiales;f__Acidimicrobiaceae;
OTU_176	p__Actinobacteria;c__Thermoleophilia;o__Gaiellales
OTU_215	p__Firmicutes;c__Clostridia;o__Clostridiales;f__Lachnospiraceae;g__Mobilitalea
OTU_272	p__Actinobacteria;c__Actinobacteria;o__Streptomycetales;f__Streptomycetaceae;g__Streptomyces
OTU_71	p__Actinobacteria;c__Thermoleophilia;o__Gaiellales;f__Gaiellaceae;g__Gaiella
OTU_957	p__Bacteroidetes;c__Sphingobacteriia;o__Sphingobacteriales;f__Chitinophagaceae;g__Parasegetibacter
OTU_136	p__Actinobacteria;c__Actinobacteria;o__Propionibacteriales;f__Nocardioidaceae;
OTU_426	p__Proteobacteria;c__Betaproteobacteria;o__Burkholderiales;f__Comamonadaceae;
OTU_102	p__Proteobacteria;c__Gammaproteobacteria;o__Xanthomonadales;f__Xanthomonadaceae;
OTU_408	p__Thaumarchaeota;c__Soil Crenarchaeotic Group(SCG)
OTU_250	p__Proteobacteria;c__Betaproteobacteria;o__Burkholderiales;f__Oxalobacteraceae;g__Massilia
OTU_202	p__Firmicutes;c__Bacilli;o__Bacillales;f__Paenibacillaceae;g__Paenibacillus
OTU_154	p__Actinobacteria;c__Thermoleophilia;o__Gaiellales;f__Gaiellaceae;g__Gaiella
OTU_211	p__Actinobacteria;c__Thermoleophilia;o__Solirubrobacterales;f__Elev-16S-1332;
OTU_416	p__Firmicutes;c__Bacilli;o__Bacillales;f__Thermoactinomycetaceae;g__Thermoactinomyces
OTU_995	p__Proteobacteria;c__Deltaproteobacteria;o__Myxococcales;f__Polyangiaceae;g__Sorangium
OTU_52	p__Actinobacteria;c__Actinobacteria;o__Propionibacteriales;f__Propionibacteriaceae;g__Microlunatus
OTU_1458	p__Proteobacteria;c__Deltaproteobacteria;o__Myxococcales;f__Phaselicystidaceae;g__Phaselicystis
OTU_309	p__Bacteroidetes;c__Sphingobacteriia;o__Sphingobacteriales;f__Chitinophagaceae;
OTU_329	p__Actinobacteria;c__Thermoleophilia;o__Gaiellales
OTU_1771	p__Firmicutes;c__Bacilli;o__Bacillales;f__Bacillaceae;g__Bacillus
OTU_346	p__Firmicutes;c__Clostridia;o__Clostridiales;f__Clostridiaceae 1;g__Clostridium sensu stricto 13
OTU_81	p__Actinobacteria;c__Thermoleophilia;o__Gaiellales;f__Gaiellaceae;g__Gaiella
OTU_617	p__Proteobacteria;c__Betaproteobacteria;o__Burkholderiales;f__Comamonadaceae;
OTU_592	p__Firmicutes;c__Bacilli;o__Bacillales
OTU_236	p__Proteobacteria;c__Alphaproteobacteria;o__Sphingomonadales;f__Erythrobacteraceae;g__Altererythrobacter
OTU_349	p__Proteobacteria;c__Betaproteobacteria;o__Burkholderiales;f__Comamonadaceae;
OTU_333	p__Actinobacteria;c__Thermoleophilia;o__Solirubrobacterales;f__319-6M6;
OTU_268	p__Proteobacteria;c__Gammaproteobacteria;o__Xanthomonadales;f__Xanthomonadales Incertae Sedis;g__Acidibacter
OTU_133	p__Bacteroidetes;c__Sphingobacteriia;o__Sphingobacteriales;f__Chitinophagaceae;g__Terrimonas
OTU_153	p__Proteobacteria;c__Betaproteobacteria;o__Methylophilales;f__Methylophilaceae;
OTU_295	p__Actinobacteria;c__Actinobacteria;o__Propionibacteriales;f__Nocardioidaceae;g__Nocardioides
OTU_819	p__Proteobacteria;c__Alphaproteobacteria;o__Rhodospirillales;f__Rhodospirillaceae;g__Skermanella
OTU_400	p__Actinobacteria;c__Actinobacteria;o__Frankiales;f__Sporichthyaceae;
OTU_414	p__Firmicutes;c__Clostridia;o__Clostridiales;f__Peptostreptococcaceae;g__Sporacetigenium
OTU_603	p__Firmicutes;c__Bacilli;o__Bacillales;f__Paenibacillaceae;g__Paenibacillus
OTU_924	p__Proteobacteria;c__Alphaproteobacteria;o__Rhodospirillales;f__Rhodospirillaceae;
OTU_418	p__Actinobacteria;c__Acidimicrobiia;o__Acidimicrobiales;f__Acidimicrobiaceae;g__Ilumatobacter

	OTU_621	p__Proteobacteria;c__Betaproteobacteria;o__TRA3-2
	OTU_565	p__Proteobacteria;c__Alphaproteobacteria;o__Rhodospirillales;f__Rhodospirillales Incertae Sedis;g__Reyranella
	OTU_328	p__Actinobacteria;c__Actinobacteria;o__Pseudonocardiales;f__Pseudonocardiaceae;g__Pseudonocardia
	OTU_188	p__Proteobacteria;c__Gammaproteobacteria;o__Xanthomonadales;f__Xanthomonadales Incertae Sedis;
	OTU_22441	p__Actinobacteria;c__Actinobacteria;o__Corynebacteriales;f__Mycobacteriaceae;g__Mycobacterium
	OTU_22371	p__Actinobacteria;c__Actinobacteria;o__Micrococcales;f__Microbacteriaceae;g__Microbacterium
	OTU_406	p__Firmicutes;c__Bacilli;o__Bacillales;f__Paenibacillaceae;g__Cohnella
	OTU_258	p__Firmicutes;c__Bacilli;o__Bacillales;f__Thermoactinomycetaceae;g__Planifilum
	OTU_345	p__Bacteroidetes;c__Cytophagia;o__Cytophagales;f__Cytophagaceae;g__Adhaeribacter
	OTU_377	p__Proteobacteria;c__Gammaproteobacteria;o__Xanthomonadales
	OTU_785	p__Actinobacteria;c__Acidimicrobiia;o__Acidimicrobiales;f__Iamiaceae;g__Iamia
	OTU_560	p__Proteobacteria;c__Alphaproteobacteria;o__Rhodospirillales;f__Rhodospirillaceae;g__Ferrovibrio
	OTU_344	p__Proteobacteria;c__Alphaproteobacteria;o__Sphingomonadales;f__Sphingomonadaceae;g__Sphingomonas
	OTU_529	p__Actinobacteria;c__Thermoleophilia;o__Solirubrobacterales;f__Elev-16S-1332;
	OTU_380	p__Bacteroidetes;c__Cytophagia;o__Cytophagales;f__Cytophagaceae;
	OTU_393	p__Actinobacteria;c__Acidimicrobiia;o__Acidimicrobiales
	OTU_244	p__Firmicutes;c__Bacilli;o__Bacillales;f__Paenibacillaceae;g__Cohnella
	OTU_58092	p__Actinobacteria;c__Actinobacteria;o__Corynebacteriales;f__Mycobacteriaceae;g__Mycobacterium
	OTU_502	p__Proteobacteria;c__Alphaproteobacteria;o__Rhizobiales;f__Xanthobacteraceae;g__Variibacter
	OTU_257	p__Actinobacteria;c__Thermoleophilia;o__Gaiellales
	OTU_531	p__Actinobacteria;c__Actinobacteria;o__Micrococcales;f__Intrasporangiaceae;
	OTU_473	p__Nitrospirae;c__Nitrospira;o__Nitrospirales;f__Nitrospiraceae;g__Nitrospira
	OTU_1534	p__Thaumarchaeota;c__Soil Crenarchaeotic Group(SCG)
	OTU_112	p__Thaumarchaeota;c__Soil Crenarchaeotic Group(SCG);o__Unknown Order;f__Unknown Family;g__Candidatus Nitrososphaera
	OTU_890	p__Actinobacteria;c__Acidimicrobiia;o__Acidimicrobiales;f__Acidimicrobiaceae;
	OTU_78	p__Bacteroidetes;c__Cytophagia;o__Cytophagales;f__Cytophagaceae;
	OTU_466	p__Proteobacteria;c__Alphaproteobacteria;o__Rhizobiales;f__Rhodobiaceae;
	OTU_302	p__Actinobacteria;c__Acidimicrobiia;o__Acidimicrobiales;f__OM1 clade;
	OTU_672	p__Actinobacteria;c__Actinobacteria;o__Streptosporangiales;f__Streptosporangiaceae;
	OTU_620	p__Bacteroidetes;c__Cytophagia;o__Cytophagales;f__Cytophagaceae;g__Ohtaekwangia
	OTU_461	p__Proteobacteria;c__Alphaproteobacteria;o__Rhizobiales;f__Xanthobacteraceae;
	OTU_512	p__Proteobacteria;c__Betaproteobacteria;o__Burkholderiales;f__Comamonadaceae;g__Aquabacterium
	OTU_239	p__Bacteroidetes;c__Sphingobacteriia;o__Sphingobacteriales;f__Chitinophagaceae;g__Lacibacter
	OTU_681	p__Proteobacteria;c__Alphaproteobacteria;o__Rhizobiales;f__Hyphomicrobiaceae;g__Hyphomicrobium
	OTU_1009	p__Proteobacteria;c__Gammaproteobacteria;o__Xanthomonadales;f__Xanthomonadaceae;
	OTU_261	p__Proteobacteria;c__Betaproteobacteria;o__Nitrosomonadales;f__Nitrosomonadaceae;
	OTU_143	p__Actinobacteria;c__Thermoleophilia;o__Gaiellales
	OTU_520	p__Proteobacteria;c__Betaproteobacteria;o__SC-I-84
	OTU_465	p__Acidobacteria;c__Holophagae;o__Subgroup 1;f__ABS-19;
	OTU_5772	p__Proteobacteria;c__Deltaproteobacteria;o__Desulfurellales;f__Desulfurellaceae;g__H16
	OTU_503	p__Proteobacteria;c__Gammaproteobacteria;o__Xanthomonadales
	OTU_145	p__Actinobacteria;c__Thermoleophilia;o__Solirubrobacterales;f__Q3-6C1;
	OTU_1478	p__Proteobacteria;c__Betaproteobacteria;o__Burkholderiales;f__Comamonadaceae;g__Aquabacterium
	OTU_379	p__Tectomicrobia;
	OTU_670	p__Actinobacteria;c__Thermoleophilia;o__Solirubrobacterales;f__FFCH1375;
	OTU_279	p__Actinobacteria;
	OTU_447	p__Proteobacteria;c__Alphaproteobacteria;o__Rhodospirillales;f__Rhodospirillaceae;g__Skermanella
	OTU_680	p__Proteobacteria;c__Alphaproteobacteria;o__Rhizobiales
	OTU_1256	p__Actinobacteria;c__Rubrobacteria;o__Rubrobacterales;f__Rubrobacteriaceae;g__Rubrobacter
	OTU_537	p__Bacteroidetes;c__Cytophagia;o__Cytophagales;f__Cytophagaceae;
	OTU_1321	p__Bacteroidetes;c__Sphingobacteriia;o__Sphingobacteriales;f__NS11-12 marine group;
	OTU_1033	p__Actinobacteria;c__Thermoleophilia;o__Solirubrobacterales
	OTU_594	p__Actinobacteria;c__Thermoleophilia;o__Gaiellales
	OTU_413	p__Actinobacteria;c__Acidimicrobiia;o__Acidimicrobiales;f__OM1 clade;
	OTU_724	p__Proteobacteria;c__Betaproteobacteria;o__Nitrosomonadales;f__Nitrosomonadaceae;
	OTU_1521	p__Actinobacteria;c__Thermoleophilia;o__Solirubrobacterales
	OTU_130	p__Actinobacteria;c__MB-A2-18
	OTU_536	p__Thaumarchaeota;c__Soil Crenarchaeotic Group(SCG)
	OTU_1360	p__Proteobacteria;c__Alphaproteobacteria;o__Rhizobiales;f__Methylobacteriaceae;g__Microvirga
	OTU_1333	p__Gemmatimonadetes;c__Gemmatimonadetes;o__Gemmatimonadales;f__Gemmatimonadaceae;
	OTU_604	p__Bacteroidetes;c__Sphingobacteriia;o__Sphingobacteriales;f__Chitinophagaceae;
	OTU_1156	p__Thaumarchaeota;c__Soil Crenarchaeotic Group(SCG)
	OTU_1194	p__Proteobacteria;c__Alphaproteobacteria;o__Rhizobiales;f__Neo-b11;
	OTU_362	p__Proteobacteria;c__Betaproteobacteria;o__Burkholderiales;f__Alcaligenaceae;
	OTU_875	p__Proteobacteria;c__Deltaproteobacteria;o__Myxococcales;f__Sandaracinaceae;
	OTU_862	p__Gemmatimonadetes;c__Gemmatimonadetes;o__Gemmatimonadales;f__Gemmatimonadaceae;

	OTU_3884	p__Gemmatimonadetes;c__Gemmatimonadetes;o__Gemmatimonadales;f__Gemmatimonadaceae;g__Gemmatimonas
	OTU_193	p__Actinobacteria;c__MB-A2-18
	OTU_1767	p__Actinobacteria;c__Thermoleophilia;o__Gaiellales
	OTU_782	p__Proteobacteria;c__Alphaproteobacteria;o__Rhizobiales;f__Methylobacteriaceae;g__Microvirga
	OTU_5766	p__Actinobacteria;c__Thermoleophilia;o__Gaiellales
	OTU_1746	p__Bacteroidetes;c__Cytophagia;o__Cytophagales;f__Cytophagaceae;g__Ohtaekwangia
	OTU_962	p__Proteobacteria;c__Gammaproteobacteria;o__Xanthomonadales;f__Xanthomonadaceae;g__Arenimonas
	OTU_2160	p__Proteobacteria;c__Deltaproteobacteria;o__Myxococcales;f__Polyangiaceae;
	OTU_273	p__Bacteroidetes;c__Cytophagia;o__Cytophagales;f__Cytophagaceae;
T0|T1	OTU_20	p__Bacteroidetes;c__Flavobacteriia;o__Flavobacteriales;f__Flavobacteriaceae;g__Chryseobacterium
	OTU_15	p__Proteobacteria;c__Gammaproteobacteria;o__Pseudomonadales;f__Pseudomonadaceae;g__Pseudomonas
	OTU_19	p__Proteobacteria;c__Alphaproteobacteria;o__Rhizobiales;f__Phyllobacteriaceae;g__Mesorhizobium
	OTU_100	p__Proteobacteria;c__Alphaproteobacteria;o__Caulobacterales;f__Caulobacteraceae;g__Caulobacter
T0|T2-KB	OTU_18	p__Firmicutes;c__Bacilli;o__Bacillales
	OTU_54	p__Firmicutes;c__Bacilli;o__Bacillales;f__Bacillaceae;g__Bacillus
T1|T2-KB	OTU_27	p__Actinobacteria;c__Actinobacteria;o__Streptomycetales;f__Streptomycetaceae;g__Streptomyces
	OTU_9	p__Actinobacteria;c__Actinobacteria;o__Micrococcales;f__Microbacteriaceae;g__Microbacterium
	OTU_56	p__Actinobacteria;c__Actinobacteria;o__Micrococcales;f__Micrococcaceae;g__Arthrobacter
	OTU_10	p__Proteobacteria;c__Betaproteobacteria;o__Burkholderiales;f__Burkholderiaceae;g__Ralstonia
	OTU_35	p__Proteobacteria;c__Alphaproteobacteria;o__Caulobacterales;f__Caulobacteraceae;g__Asticcacaulis
	OTU_468	p__Proteobacteria;
	OTU_50	p__Proteobacteria;c__Betaproteobacteria;o__Methylophilales;f__Methylophilaceae;
	OTU_67	p__Actinobacteria;c__Actinobacteria;o__Pseudonocardiales;f__Pseudonocardiaceae;
	OTU_68	p__Actinobacteria;c__Actinobacteria;o__Micrococcales;f__Microbacteriaceae;
	OTU_1252	p__Proteobacteria;c__Alphaproteobacteria;o__Rhizobiales;f__Hyphomicrobiaceae;g__Devosia
	OTU_44	p__Bacteroidetes;c__Sphingobacteriia;o__Sphingobacteriales;f__Chitinophagaceae;g__Niastella
	OTU_88	p__Proteobacteria;c__Alphaproteobacteria;o__Rhizobiales;f__Phyllobacteriaceae;g__Mesorhizobium
	OTU_65	p__Proteobacteria;c__Alphaproteobacteria;o__Rhizobiales;f__Rhizobiaceae;g__Rhizobium
	OTU_212	p__Proteobacteria;c__Betaproteobacteria;o__Burkholderiales;f__Comamonadaceae;g__Hydrogenophaga
	OTU_83	p__Proteobacteria;c__Alphaproteobacteria;o__Rhizobiales;f__Hyphomicrobiaceae;g__Devosia
	OTU_147	p__Proteobacteria;c__Alphaproteobacteria;o__Rhizobiales;f__Bradyrhizobiaceae;
	OTU_165	p__Proteobacteria;c__Gammaproteobacteria;o__Pseudomonadales;f__Moraxellaceae;
	OTU_194	p__Proteobacteria;c__Alphaproteobacteria;o__Sphingomonadales;f__Sphingomonadaceae;g__Novosphingobium
	OTU_64	p__Proteobacteria;c__Alphaproteobacteria;o__Rhizobiales;f__Rhizobiaceae;g__Shinella
	OTU_641	p__Proteobacteria;c__Alphaproteobacteria;o__Rhizobiales;f__Phyllobacteriaceae;
	OTU_148	p__Proteobacteria;c__Alphaproteobacteria;o__Rhizobiales;f__Hyphomicrobiaceae;g__Devosia
	OTU_501	p__Actinobacteria;c__Acidimicrobiia;o__Acidimicrobiales;f__Acidimicrobiaceae;
	OTU_85	p__Proteobacteria;c__Alphaproteobacteria;o__Sphingomonadales;f__Sphingomonadaceae;g__Sphingopyxis
	OTU_223	p__Actinobacteria;c__Acidimicrobiia;o__Acidimicrobiales;f__Iamiaceae;g__Iamia
	OTU_179	p__Proteobacteria;c__Alphaproteobacteria;o__Rhizobiales;f__Phyllobacteriaceae;
	OTU_69	p__Actinobacteria;c__Actinobacteria;o__Propionibacteriales;f__Nocardioidaceae;g__Nocardioides
	OTU_141	p__Actinobacteria;c__Thermoleophilia;o__Solirubrobacterales;f__Gsoil-1167;
	OTU_174	p__Actinobacteria;c__Actinobacteria;o__Corynebacteriales;f__Mycobacteriaceae;g__Mycobacterium
T0|T1|T2-KB	OTU_97	p__Actinobacteria;c__Actinobacteria;o__Propionibacteriales;f__Nocardioidaceae;
	OTU_3	p__Proteobacteria;c__Alphaproteobacteria;o__Rhizobiales;f__Rhizobiaceae;g__Rhizobium
	OTU_8	p__Actinobacteria;c__Actinobacteria;o__Micrococcales;f__Micrococcaceae;g__Pseudarthrobacter
	OTU_1	p__Firmicutes;c__Bacilli;o__Bacillales;f__Bacillaceae;g__Bacillus
	OTU_22	p__Proteobacteria;c__Betaproteobacteria;o__Burkholderiales;f__Comamonadaceae;g__Variovorax

	**Karnabrunn**	
T0	OTU_4	p__Firmicutes;c__Bacilli;o__Bacillales;f__Staphylococcaceae;g__Staphylococcus
	OTU_13	p__Proteobacteria;c__Gammaproteobacteria;o__Pseudomonadales;f__Moraxellaceae;g__Acinetobacter
T1	OTU_58	p__Actinobacteria;c__Actinobacteria;o__Micrococcales;f__Microbacteriaceae;
	OTU_109	p__Actinobacteria;c__Actinobacteria;o__Propionibacteriales;f__Propionibacteriaceae;g__Propionibacterium
	OTU_10	p__Proteobacteria;c__Betaproteobacteria;o__Burkholderiales;f__Burkholderiaceae;g__Ralstonia
	OTU_43	p__Bacteroidetes;c__Cytophagia;o__Cytophagales;f__Cytophagaceae;
	OTU_16	p__Bacteroidetes;c__Cytophagia;o__Cytophagales;f__Cytophagaceae;g__Emticicia
	OTU_468	p__Proteobacteria;
	OTU_90	p__Bacteroidetes;c__Flavobacteriia;o__Flavobacteriales;f__Flavobacteriaceae;g__Chryseobacterium
	OTU_464	p__Actinobacteria;c__Acidimicrobiia;o__Acidimicrobiales;
	OTU_755	p__Actinobacteria;c__Acidimicrobiia;o__Acidimicrobiales;
	OTU_131	p__Actinobacteria;c__Actinobacteria;o__Streptosporangiales;f__Thermomonosporaceae;
	OTU_155	p__Bacteroidetes;c__Sphingobacteriia;o__Sphingobacteriales;f__Chitinophagaceae;g__Chitinophaga
	OTU_119	p__Proteobacteria;c__Alphaproteobacteria;o__Rhizobiales;f__Rhizobiaceae;g__Shinella
	OTU_319	p__Actinobacteria;c__Acidimicrobiia;o__Acidimicrobiales;f__Iamiaceae;g__Iamia
	OTU_350	p__Proteobacteria;c__Alphaproteobacteria;o__Rhizobiales;f__Xanthobacteraceae;g__Pseudolabrys

	OTU_364	p__Proteobacteria;c__Alphaproteobacteria;o__Caulobacterales;f__Caulobacteraceae;g__Brevundimonas
	OTU_238	p__Proteobacteria;c__Alphaproteobacteria;o__Rhizobiales;f__Bradyrhizobiaceae;g__Bosea
	OTU_876	p__Actinobacteria;c__Actinobacteria;o__Micrococcales;f__Microbacteriaceae;g__Leifsonia
	OTU_195	p__Proteobacteria;c__Alphaproteobacteria;o__Rhodospirillales;f__Rhodospirillaceae;g__Dongia
	OTU_509	p__Actinobacteria;c__Thermoleophilia;o__Solirubrobacterales;f__Elev-16S-1332;
	OTU_140	p__Proteobacteria;c__Alphaproteobacteria;o__Caulobacterales;f__Caulobacteraceae;g__Brevundimonas
	OTU_518	p__Proteobacteria;c__Alphaproteobacteria;o__Rhizobiales;f__Rhizobiales Incertae Sedis;g__Bauldia
	OTU_230	p__Actinobacteria;c__Acidimicrobiia;o__Acidimicrobiales;f__Iamiaceae;g__Iamia
	OTU_178	p__Actinobacteria;c__Actinobacteria;o__Propionibacteriales;f__Nocardioidaceae;g__Kribbella
	OTU_2846	p__Actinobacteria;c__Actinobacteria;o__Micrococcales;f__Microbacteriaceae;g__Microbacterium
	OTU_75	p__Bacteroidetes;c__Sphingobacteriia;o__Sphingobacteriales;f__Chitinophagaceae;g__Chitinophaga
	OTU_108	p__Bacteroidetes;c__Sphingobacteriia;o__Sphingobacteriales;f__Chitinophagaceae;g__Terrimonas
	OTU_505	p__Proteobacteria;c__Alphaproteobacteria;o__Rhodospirillales;f__Rhodospirillales Incertae Sedis;g__Reyranella
	OTU_501	p__Actinobacteria;c__Acidimicrobiia;o__Acidimicrobiales;f__Acidimicrobiaceae;
	OTU_527	p__Proteobacteria;c__Alphaproteobacteria;o__Sphingomonadales;f__Erythrobacteraceae;g__Altererythrobacter
	OTU_601	p__Proteobacteria;c__Betaproteobacteria;o__Burkholderiales;f__Comamonadaceae;
	OTU_618	p__Proteobacteria;c__Alphaproteobacteria;o__Rhizobiales;f__Methylobacteriaceae;
	OTU_179	p__Proteobacteria;c__Alphaproteobacteria;o__Rhizobiales;f__Phyllobacteriaceae;
	OTU_252	p__Proteobacteria;c__Alphaproteobacteria;o__Rhizobiales;f__Brucellaceae;g__Ochrobactrum
	OTU_1094	p__Proteobacteria;c__Gammaproteobacteria;o__Xanthomonadales;f__Xanthomonadaceae;g__Dokdonella
	OTU_4010	p__Proteobacteria;c__Alphaproteobacteria;o__Rhizobiales;f__Hyphomicrobiaceae;g__Devosia
	OTU_1288	p__Proteobacteria;c__Alphaproteobacteria;o__Rhizobiales;f__Rhizobiales Incertae Sedis;g__Bauldia
	OTU_1267	p__Proteobacteria;c__Alphaproteobacteria;o__Rhizobiales;
	OTU_1600	p__Proteobacteria;c__Alphaproteobacteria;o__Rhizobiales;f__Hyphomicrobiaceae;
	OTU_357	p__Proteobacteria;c__Alphaproteobacteria;o__Rhizobiales;f__Rhizobiales Incertae Sedis;g__Rhizomicrobium
T2-K	OTU_33	p__Proteobacteria;c__Betaproteobacteria;o__Burkholderiales;f__Comamonadaceae;
	OTU_39	p__Bacteroidetes;c__Sphingobacteriia;o__Sphingobacteriales;f__Chitinophagaceae;g__Terrimonas
	OTU_30	p__Saccharibacteria;
	OTU_28	p__Actinobacteria;c__Actinobacteria;o__Micrococcales;f__Microbacteriaceae;g__Agromyces
	OTU_34	p__Actinobacteria;c__Actinobacteria;o__Micrococcales;f__Micrococcaceae;
	OTU_142	p__Proteobacteria;c__Alphaproteobacteria;o__Caulobacterales;f__Caulobacteraceae;
	OTU_270	p__Actinobacteria;c__Actinobacteria;o__Streptomycetales;f__Streptomycetaceae;g__Streptomyces
	OTU_1461	p__Actinobacteria;c__Actinobacteria;o__Streptomycetales;f__Streptomycetaceae;g__Streptomyces
	OTU_425	p__Actinobacteria;c__Actinobacteria;o__Streptomycetales;f__Streptomycetaceae;g__Streptomyces
	OTU_74	p__Proteobacteria;c__Betaproteobacteria;o__Burkholderiales;f__Comamonadaceae;
	OTU_25	p__Actinobacteria;c__Actinobacteria;o__Micrococcales;f__Micrococcaceae;
	OTU_630	p__Actinobacteria;c__Actinobacteria;o__Propionibacteriales;f__Nocardioidaceae;g__Nocardioides
	OTU_124	p__Actinobacteria;c__Actinobacteria;o__Propionibacteriales;f__Nocardioidaceae;g__Nocardioides
	OTU_221	p__Proteobacteria;c__Alphaproteobacteria;o__Rhizobiales;f__Rhizobiaceae;
	OTU_225	p__Proteobacteria;c__Betaproteobacteria;o__Burkholderiales;f__Oxalobacteraceae;g__Paucimonas
	OTU_773	p__Proteobacteria;c__Betaproteobacteria;o__Burkholderiales;f__Comamonadaceae;
	OTU_4677	p__Proteobacteria;c__Gammaproteobacteria;o__Xanthomonadales;f__Xanthomonadaceae;
	OTU_280	p__Actinobacteria;c__Actinobacteria;o__Frankiales;f__Geodermatophilaceae;g__Blastococcus
	OTU_53	p__Proteobacteria;c__Alphaproteobacteria;o__Caulobacterales;f__Caulobacteraceae;g__Caulobacter
	OTU_266	p__Bacteroidetes;c__Sphingobacteriia;o__Sphingobacteriales;f__Chitinophagaceae;
	OTU_654	p__Bacteroidetes;c__Sphingobacteriia;o__Sphingobacteriales;f__Chitinophagaceae;
	OTU_383	p__Proteobacteria;c__Deltaproteobacteria;o__Myxococcales;f__BIrii41;
	OTU_63	p__Actinobacteria;c__Actinobacteria;o__Frankiales;f__Geodermatophilaceae;
	OTU_23	p__Firmicutes;c__Bacilli;o__Bacillales;f__Bacillaceae;g__Bacillus
	OTU_1811	p__Proteobacteria;c__Alphaproteobacteria;o__Rhizobiales;f__Methylobacteriaceae;g__Microvirga
	OTU_172	p__Proteobacteria;c__Betaproteobacteria;o__Rhodocyclales;f__Rhodocyclaceae;
	OTU_2150	p__Actinobacteria;c__Actinobacteria;o__Micrococcales;
	OTU_246	p__Actinobacteria;c__Actinobacteria;o__Propionibacteriales;f__Nocardioidaceae;g__Nocardioides
	OTU_158	p__Firmicutes;c__Bacilli;o__Bacillales;
	OTU_581	p__Bacteroidetes;c__Cytophagia;o__Cytophagales;f__Cytophagaceae;g__Ohtaekwangia
	OTU_340	p__Proteobacteria;c__Gammaproteobacteria;o__Pseudomonadales;f__Moraxellaceae;
	OTU_700	p__Bacteroidetes;c__Sphingobacteriia;o__Sphingobacteriales;f__Chitinophagaceae;g__Flavitalea
	OTU_432	p__Firmicutes;c__Bacilli;o__Bacillales;f__Paenibacillaceae;g__Brevibacillus
	OTU_313	p__Proteobacteria;c__Alphaproteobacteria;o__Rhizobiales;f__Rhizobiaceae;g__Ensifer
	OTU_51	p__Proteobacteria;c__Alphaproteobacteria;o__Sphingomonadales;f__Sphingomonadaceae;g__Sphingomonas
	OTU_92	p__Proteobacteria;c__Alphaproteobacteria;o__Caulobacterales;f__Caulobacteraceae;g__Caulobacter
	OTU_381	p__Bacteroidetes;c__Sphingobacteriia;o__Sphingobacteriales;f__Chitinophagaceae;g__Niastella
	OTU_277	p__Bacteroidetes;c__Cytophagia;o__Cytophagales;f__Cytophagaceae;g__Dyadobacter
	OTU_1680	p__Firmicutes;c__Clostridia;o__Clostridiales;f__Peptostreptococcaceae;
	OTU_728	p__Actinobacteria;c__Acidimicrobiia;o__Acidimicrobiales;
	OTU_428	p__Firmicutes;c__Bacilli;o__Bacillales;f__Bacillaceae;g__Bacillus
	OTU_79	p__Thaumarchaeota;c__Soil Crenarchaeotic Group(SCG);

	OTU_588	p__Proteobacteria;c__Alphaproteobacteria;o__Rhizobiales;f__Xanthobacteraceae;g__Variibacter
	OTU_570	p__Proteobacteria;c__Alphaproteobacteria;o__Caulobacterales;f__Caulobacteraceae;g__Phenylobacterium
	OTU_128	p__Bacteroidetes;c__Sphingobacteriia;o__Sphingobacteriales;f__Sphingobacteriaceae;
	OTU_12	p__Thaumarchaeota;c__Soil Crenarchaeotic Group(SCG);
	OTU_192	p__Firmicutes;c__Bacilli;o__Bacillales;f__Paenibacillaceae;g__Paenibacillus
	OTU_1215	p__Bacteroidetes;c__Sphingobacteriia;o__Sphingobacteriales;f__Chitinophagaceae;
	OTU_386	p__Bacteroidetes;c__Sphingobacteriia;o__Sphingobacteriales;f__Chitinophagaceae;
	OTU_370	p__Bacteroidetes;c__Cytophagia;o__Cytophagales;f__Cytophagaceae;
	OTU_42	p__Firmicutes;c__Bacilli;o__Bacillales;f__Paenibacillaceae;g__Paenibacillus
	OTU_190	p__Proteobacteria;c__Alphaproteobacteria;o__Rhizobiales;f__Phyllobacteriaceae;g__Phyllobacterium
	OTU_116	p__Firmicutes;c__Bacilli;o__Bacillales;f__Paenibacillaceae;g__Paenibacillus
	OTU_59	p__Actinobacteria;c__Rubrobacteria;o__Rubrobacterales;f__Rubrobacteriaceae;g__Rubrobacter
	OTU_134	p__Actinobacteria;c__Actinobacteria;o__Propionibacteriales;f__Nocardioidaceae;g__Nocardioides
	OTU_7	p__Thaumarchaeota;c__Soil Crenarchaeotic Group(SCG);
	OTU_384	p__Actinobacteria;c__Thermoleophilia;o__Solirubrobacterales;f__Solirubrobacteraceae;g__Solirubrobacter
	OTU_296	p__Proteobacteria;c__Betaproteobacteria;o__Nitrosomonadales;f__Nitrosomonadaceae;
	OTU_348	p__Actinobacteria;c__Actinobacteria;o__Micromonosporales;f__Micromonosporaceae;
	OTU_186	p__Proteobacteria;c__Alphaproteobacteria;o__Rhizobiales;f__Bradyrhizobiaceae;g__Bradyrhizobium
	OTU_276	p__Proteobacteria;c__Alphaproteobacteria;o__Rhizobiales;f__Hyphomicrobiaceae;g__Devosia
	OTU_229	p__Actinobacteria;c__Actinobacteria;o__Corynebacteriales;f__Nocardiaceae;g__Rhodococcus
	OTU_515	p__Proteobacteria;c__Deltaproteobacteria;o__Myxococcales;f__Myxococcaceae;
	OTU_227	p__Nitrospirae;c__Nitrospira;o__Nitrospirales;f__Nitrospiraceae;g__Nitrospira
	OTU_104	p__Proteobacteria;c__Gammaproteobacteria;o__Cellvibrionales;f__Cellvibrionaceae;g__Cellvibrio
	OTU_200	p__Bacteroidetes;c__Sphingobacteriia;o__Sphingobacteriales;f__Chitinophagaceae;g__Ferruginibacter
	OTU_118	p__Actinobacteria;c__Actinobacteria;o__Micromonosporales;f__Micromonosporaceae;
	OTU_480	p__Gemmatimonadetes;c__Gemmatimonadetes;o__Gemmatimonadales;f__Gemmatimonadaceae;
	OTU_37	p__Firmicutes;c__Bacilli;o__Bacillales;f__Planococcaceae;g__Paenisporosarcina
	OTU_352	p__Proteobacteria;c__Betaproteobacteria;o__Burkholderiales;f__Oxalobacteraceae;
	OTU_46	p__Thaumarchaeota;c__Soil Crenarchaeotic Group(SCG);
	OTU_324	p__Firmicutes;c__Bacilli;o__Bacillales;f__Paenibacillaceae;g__Paenibacillus
	OTU_123	p__Actinobacteria;c__Actinobacteria;o__Propionibacteriales;f__Nocardioidaceae;g__Nocardioides
	OTU_113	p__Actinobacteria;c__Actinobacteria;o__Corynebacteriales;f__Nocardiaceae;g__Nocardia
	OTU_686	p__Proteobacteria;c__Gammaproteobacteria;o__Xanthomonadales;f__Xanthomonadales Incertae Sedis;
	OTU_312	p__Bacteroidetes;c__Sphingobacteriia;o__Sphingobacteriales;f__Chitinophagaceae;g__Parafilimonas
	OTU_979	p__Proteobacteria;c__Alphaproteobacteria;o__Rhizobiales;f__Hyphomicrobiaceae;g__Rhodoplanes
	OTU_231	p__Actinobacteria;c__Acidimicrobiia;o__Acidimicrobiales;f__Acidimicrobiaceae;
	OTU_334	p__Gemmatimonadetes;c__Gemmatimonadetes;o__Gemmatimonadales;f__Gemmatimonadaceae;
	OTU_176	p__Actinobacteria;c__Thermoleophilia;o__Gaiellales;
	OTU_215	p__Firmicutes;c__Clostridia;o__Clostridiales;f__Lachnospiraceae;g__Mobilitalea
	OTU_3454	p__Bacteroidetes;c__Sphingobacteriia;o__Sphingobacteriales;f__Chitinophagaceae;
	OTU_71	p__Actinobacteria;c__Thermoleophilia;o__Gaiellales;f__Gaiellaceae;g__Gaiella
	OTU_136	p__Actinobacteria;c__Actinobacteria;o__Propionibacteriales;f__Nocardioidaceae;
	OTU_426	p__Proteobacteria;c__Betaproteobacteria;o__Burkholderiales;f__Comamonadaceae;
	OTU_102	p__Proteobacteria;c__Gammaproteobacteria;o__Xanthomonadales;f__Xanthomonadaceae;
	OTU_304	p__Firmicutes;c__Bacilli;o__Bacillales;f__Bacillaceae;g__Bacillus
	OTU_408	p__Thaumarchaeota;c__Soil Crenarchaeotic Group(SCG);
	OTU_873	p__Proteobacteria;c__Alphaproteobacteria;o__Rhizobiales;f__Hyphomicrobiaceae;
	OTU_202	p__Firmicutes;c__Bacilli;o__Bacillales;f__Paenibacillaceae;g__Paenibacillus
	OTU_154	p__Actinobacteria;c__Thermoleophilia;o__Gaiellales;f__Gaiellaceae;g__Gaiella
	OTU_211	p__Actinobacteria;c__Thermoleophilia;o__Solirubrobacterales;f__Elev-16S-1332;
	OTU_52	p__Actinobacteria;c__Actinobacteria;o__Propionibacteriales;f__Propionibacteriaceae;g__Microlunatus
	OTU_309	p__Bacteroidetes;c__Sphingobacteriia;o__Sphingobacteriales;f__Chitinophagaceae;
	OTU_329	p__Actinobacteria;c__Thermoleophilia;o__Gaiellales;
	OTU_616	p__Proteobacteria;c__Alphaproteobacteria;o__Rhizobiales;f__Phyllobacteriaceae;
	OTU_346	p__Firmicutes;c__Clostridia;o__Clostridiales;f__Clostridiaceae 1;g__Clostridium sensu stricto 13
	OTU_538	p__Actinobacteria;c__Actinobacteria;o__Micromonosporales;f__Micromonosporaceae;
	OTU_81	p__Actinobacteria;c__Thermoleophilia;o__Gaiellales;f__Gaiellaceae;g__Gaiella
	OTU_617	p__Proteobacteria;c__Betaproteobacteria;o__Burkholderiales;f__Comamonadaceae;
	OTU_236	p__Proteobacteria;c__Alphaproteobacteria;o__Sphingomonadales;f__Erythrobacteraceae;g__Altererythrobacter
	OTU_268	p__Proteobacteria;c__Gammaproteobacteria;o__Xanthomonadales;f__Xanthomonadales Incertae Sedis;g__Acidibacter
	OTU_259	p__Actinobacteria;c__Actinobacteria;o__Propionibacteriales;f__Nocardioidaceae;
	OTU_3809	p__Thaumarchaeota;c__Soil Crenarchaeotic Group(SCG);
	OTU_133	p__Bacteroidetes;c__Sphingobacteriia;o__Sphingobacteriales;f__Chitinophagaceae;g__Terrimonas
	OTU_691	p__Bacteroidetes;c__Sphingobacteriia;o__Sphingobacteriales;f__Chitinophagaceae;
	OTU_153	p__Proteobacteria;c__Betaproteobacteria;o__Methylophilales;f__Methylophilaceae;
	OTU_295	p__Actinobacteria;c__Actinobacteria;o__Propionibacteriales;f__Nocardioidaceae;g__Nocardioides

OTU_819	p__Proteobacteria;c__Alphaproteobacteria;o__Rhodospirillales;f__Rhodospirillaceae;g__Skermanella
OTU_400	p__Actinobacteria;c__Actinobacteria;o__Frankiales;f__Sporichthyaceae;
OTU_414	p__Firmicutes;c__Clostridia;o__Clostridiales;f__Peptostreptococcaceae;g__Sporacetigenium
OTU_603	p__Firmicutes;c__Bacilli;o__Bacillales;f__Paenibacillaceae;g__Paenibacillus
OTU_924	p__Proteobacteria;c__Alphaproteobacteria;o__Rhodospirillales;f__Rhodospirillaceae;
OTU_418	p__Actinobacteria;c__Acidimicrobiia;o__Acidimicrobiales;f__Acidimicrobiaceae;g__Ilumatobacter
OTU_565	p__Proteobacteria;c__Alphaproteobacteria;o__Rhodospirillales;f__Rhodospirillales Incertae Sedis;g__Reyranella
OTU_524	p__Proteobacteria;c__Deltaproteobacteria;o__Bdellovibrionales;f__Bacteriovoracaceae;g__Peredibacter
OTU_328	p__Actinobacteria;c__Actinobacteria;o__Pseudonocardiales;f__Pseudonocardiaceae;g__Pseudonocardia
OTU_188	p__Proteobacteria;c__Gammaproteobacteria;o__Xanthomonadales;f__Xanthomonadales Incertae Sedis;
OTU_22371	p__Actinobacteria;c__Actinobacteria;o__Micrococcales;f__Microbacteriaceae;g__Microbacterium
OTU_406	p__Firmicutes;c__Bacilli;o__Bacillales;f__Paenibacillaceae;g__Cohnella
OTU_1305	p__Proteobacteria;c__Gammaproteobacteria;o__Xanthomonadales;f__Xanthomonadales Incertae Sedis;
OTU_1659	p__Proteobacteria;c__Deltaproteobacteria;o__Bdellovibrionales;f__Bacteriovoracaceae;g__Peredibacter
OTU_345	p__Bacteroidetes;c__Cytophagia;o__Cytophagales;f__Cytophagaceae;g__Adhaeribacter
OTU_377	p__Proteobacteria;c__Gammaproteobacteria;o__Xanthomonadales;
OTU_785	p__Actinobacteria;c__Acidimicrobiia;o__Acidimicrobiales;f__Iamiaceae;g__Iamia
OTU_560	p__Proteobacteria;c__Alphaproteobacteria;o__Rhodospirillales;f__Rhodospirillaceae;g__Ferrovibrio
OTU_9738	p__Actinobacteria;c__Actinobacteria;o__Streptosporangiales;f__Streptosporangiaceae;
OTU_318	p__Actinobacteria;c__Actinobacteria;o__Corynebacteriales;f__Mycobacteriaceae;g__Mycobacterium
OTU_344	p__Proteobacteria;c__Alphaproteobacteria;o__Sphingomonadales;f__Sphingomonadaceae;g__Sphingomonas
OTU_1304	p__Actinobacteria;c__Actinobacteria;o__Micromonosporales;f__Micromonosporaceae;
OTU_529	p__Actinobacteria;c__Thermoleophilia;o__Solirubrobacterales;f__Elev-16S-1332;
OTU_380	p__Bacteroidetes;c__Cytophagia;o__Cytophagales;f__Cytophagaceae;
OTU_500	p__Proteobacteria;c__Gammaproteobacteria;o__Xanthomonadales;f__Xanthomonadaceae;
OTU_393	p__Actinobacteria;c__Acidimicrobiia;o__Acidimicrobiales;
OTU_1244	p__Proteobacteria;c__Gammaproteobacteria;o__Pseudomonadales;f__Pseudomonadaceae;
OTU_14606	p__Proteobacteria;c__Betaproteobacteria;o__Burkholderiales;f__Comamonadaceae;
OTU_244	p__Firmicutes;c__Bacilli;o__Bacillales;f__Paenibacillaceae;g__Cohnella
OTU_2446	p__Proteobacteria;c__Gammaproteobacteria;o__Xanthomonadales;f__Xanthomonadaceae;g__Lysobacter
OTU_1524	p__Proteobacteria;c__Gammaproteobacteria;o__Xanthomonadales;f__Xanthomonadaceae;g__Arenimonas
OTU_502	p__Proteobacteria;c__Alphaproteobacteria;o__Rhizobiales;f__Xanthobacteraceae;g__Variibacter
OTU_257	p__Actinobacteria;c__Thermoleophilia;o__Gaiellales;
OTU_531	p__Actinobacteria;c__Actinobacteria;o__Micrococcales;f__Intrasporangiaceae;
OTU_473	p__Nitrospirae;c__Nitrospira;o__Nitrospirales;f__Nitrospiraceae;g__Nitrospira
OTU_1534	p__Thaumarchaeota;c__Soil Crenarchaeotic Group(SCG);
OTU_112	p__Thaumarchaeota;c__Soil Crenarchaeotic Group(SCG);o__Unknown Order;f__Unknown Family;g__Candidatus Nitrososphaera
OTU_890	p__Actinobacteria;c__Acidimicrobiia;o__Acidimicrobiales;f__Acidimicrobiaceae;
OTU_78	p__Bacteroidetes;c__Cytophagia;o__Cytophagales;f__Cytophagaceae;
OTU_466	p__Proteobacteria;c__Alphaproteobacteria;o__Rhizobiales;f__Rhodobiaceae;
OTU_19054	p__Bacteroidetes;c__Sphingobacteriia;o__Sphingobacteriales;f__Sphingobacteriaceae;
OTU_5804	p__Proteobacteria;c__Gammaproteobacteria;o__Xanthomonadales;f__Xanthomonadales Incertae Sedis;
OTU_822	p__Proteobacteria;c__Alphaproteobacteria;o__Rhizobiales;f__Methylobacteriaceae;g__Microvirga
OTU_302	p__Actinobacteria;c__Acidimicrobiia;o__Acidimicrobiales;f__OM1 clade;
OTU_672	p__Actinobacteria;c__Actinobacteria;o__Streptosporangiales;f__Streptosporangiaceae;
OTU_620	p__Bacteroidetes;c__Cytophagia;o__Cytophagales;f__Cytophagaceae;g__Ohtaekwangia
OTU_398	p__Actinobacteria;c__Actinobacteria;o__Frankiales;
OTU_512	p__Proteobacteria;c__Betaproteobacteria;o__Burkholderiales;f__Comamonadaceae;g__Aquabacterium
OTU_1621	p__Actinobacteria;c__Acidimicrobiia;o__Acidimicrobiales;f__Acidimicrobiaceae;
OTU_239	p__Bacteroidetes;c__Sphingobacteriia;o__Sphingobacteriales;f__Chitinophagaceae;g__Lacibacter
OTU_667	p__Bacteroidetes;c__Sphingobacteriia;o__Sphingobacteriales;f__Chitinophagaceae;g__Flavitalea
OTU_681	p__Proteobacteria;c__Alphaproteobacteria;o__Rhizobiales;f__Hyphomicrobiaceae;g__Hyphomicrobium
OTU_1009	p__Proteobacteria;c__Gammaproteobacteria;o__Xanthomonadales;f__Xanthomonadaceae;
OTU_261	p__Proteobacteria;c__Betaproteobacteria;o__Nitrosomonadales;f__Nitrosomonadaceae;
OTU_143	p__Actinobacteria;c__Thermoleophilia;o__Gaiellales;
OTU_465	p__Acidobacteria;c__Holophagae;o__Subgroup 1f__ABS-19;
OTU_5772	p__Proteobacteria;c__Deltaproteobacteria;o__Desulfurellales;f__Desulfurellaceae;g__H16
OTU_503	p__Proteobacteria;c__Gammaproteobacteria;o__Xanthomonadales;
OTU_145	p__Actinobacteria;c__Thermoleophilia;o__Solirubrobacterales;f__Q3-6C1;
OTU_563	p__Proteobacteria;c__Alphaproteobacteria;o__Rhizobiales;f__Rhodobiaceae;
OTU_379	p__Tectomicrobia;
OTU_670	p__Actinobacteria;c__Thermoleophilia;o__Solirubrobacterales;f__FFCH1375;
OTU_279	p__Actinobacteria;
OTU_447	p__Proteobacteria;c__Alphaproteobacteria;o__Rhodospirillales;f__Rhodospirillaceae;g__Skermanella
OTU_680	p__Proteobacteria;c__Alphaproteobacteria;o__Rhizobiales;
OTU_1256	p__Actinobacteria;c__Rubrobacteria;o__Rubrobacterales;f__Rubrobacteriaceae;g__Rubrobacter

	OTU_299	p__Bacteroidetes;c__Cytophagia;o__Cytophagales;f__Cytophagaceae;g__Pontibacter
	OTU_448	p__Actinobacteria;c__Thermoleophilia;o__Gaiellales;
	OTU_741	p__Actinobacteria;c__Actinobacteria;o__Propionibacteriales;f__Nocardioidaceae;g__Nocardioides
	OTU_360	p__Actinobacteria;c__Acidimicrobiia;o__Acidimicrobiales;f__OM1 clade;
	OTU_725	p__Bacteroidetes;c__Cytophagia;o__Cytophagales;f__Cytophagaceae;
	OTU_537	p__Bacteroidetes;c__Cytophagia;o__Cytophagales;f__Cytophagaceae;
	OTU_1033	p__Actinobacteria;c__Thermoleophilia;o__Solirubrobacterales;
	OTU_413	p__Actinobacteria;c__Acidimicrobiia;o__Acidimicrobiales;f__OM1 clade;
	OTU_1367	p__Armatimonadetes;c__Fimbriimonadia;o__Fimbriimonadales;f__Fimbriimonadaceae;
	OTU_382	p__Actinobacteria;c__Thermoleophilia;o__Gaiellales;
	OTU_1521	p__Actinobacteria;c__Thermoleophilia;o__Solirubrobacterales;
	OTU_130	p__Actinobacteria;c__MB-A2-18;
	OTU_536	p__Thaumarchaeota;c__Soil Crenarchaeotic Group(SCG);
	OTU_1333	p__Gemmatimonadetes;c__Gemmatimonadetes;o__Gemmatimonadales;f__Gemmatimonadaceae;
	OTU_7522	p__Bacteroidetes;c__Cytophagia;o__Cytophagales;f__Cytophagaceae;g__Ohtaekwangia
	OTU_604	p__Bacteroidetes;c__Sphingobacteriia;o__Sphingobacteriales;f__Chitinophagaceae;
	OTU_1156	p__Thaumarchaeota;c__Soil Crenarchaeotic Group(SCG);
	OTU_1024	p__Proteobacteria;c__Betaproteobacteria;o__TRA3-2
	OTU_362	p__Proteobacteria;c__Betaproteobacteria;o__Burkholderiales;f__Alcaligenaceae;
	OTU_321	p__Tectomicrobia;
	OTU_862	p__Gemmatimonadetes;c__Gemmatimonadetes;o__Gemmatimonadales;f__Gemmatimonadaceae;
	OTU_7063	p__Bacteroidetes;c__Sphingobacteriia;o__Sphingobacteriales;f__Chitinophagaceae;
	OTU_193	p__Actinobacteria;c__MB-A2-18;
	OTU_635	p__Proteobacteria;c__Alphaproteobacteria;o__Rhizobiales;f__Rhizobiales Incertae Sedis;g__Nordella
	OTU_1767	p__Actinobacteria;c__Thermoleophilia;o__Gaiellales;
	OTU_782	p__Proteobacteria;c__Alphaproteobacteria;o__Rhizobiales;f__Methylobacteriaceae;g__Microvirga
	OTU_1281	p__Proteobacteria;c__Deltaproteobacteria;o__Desulfurellales;f__Desulfurellaceae;g__G55
	OTU_938	p__Bacteroidetes;c__Cytophagia;o__Cytophagales;f__Cytophagaceae;
	OTU_962	p__Proteobacteria;c__Gammaproteobacteria;o__Xanthomonadales;f__Xanthomonadaceae;g__Arenimonas
	OTU_2160	p__Proteobacteria;c__Deltaproteobacteria;o__Myxococcales;f__Polyangiaceae;
	OTU_273	p__Bacteroidetes;c__Cytophagia;o__Cytophagales;f__Cytophagaceae;
	OTU_513	p__Chloroflexi;c__Chloroflexia;o__Chloroflexales;f__Roseiflexaceae;g__Roseiflexus
T0|T1	OTU_20	p__Bacteroidetes;c__Flavobacteriia;o__Flavobacteriales;f__Flavobacteriaceae;g__Chryseobacterium
	OTU_15	p__Proteobacteria;c__Gammaproteobacteria;o__Pseudomonadales;f__Pseudomonadaceae;g__Pseudomonas
	OTU_19	p__Proteobacteria;c__Alphaproteobacteria;o__Rhizobiales;f__Phyllobacteriaceae;g__Mesorhizobium
	OTU_100	p__Proteobacteria;c__Alphaproteobacteria;o__Caulobacterales;f__Caulobacteraceae;g__Caulobacter
T0|T2-K	OTU_18	p__Firmicutes;c__Bacilli;o__Bacillales;
	OTU_54	p__Firmicutes;c__Bacilli;o__Bacillales;f__Bacillaceae;g__Bacillus
T1|T2-K	OTU_27	p__Actinobacteria;c__Actinobacteria;o__Streptomycetales;f__Streptomycetaceae;g__Streptomyces
	OTU_9	p__Actinobacteria;c__Actinobacteria;o__Micrococcales;f__Microbacteriaceae;g__Microbacterium
	OTU_56	p__Actinobacteria;c__Actinobacteria;o__Micrococcales;f__Micrococcaceae;g__Arthrobacter
	OTU_17	p__Saccharibacteria;
	OTU_35	p__Proteobacteria;c__Alphaproteobacteria;o__Caulobacterales;f__Caulobacteraceae;g__Asticcacaulis
	OTU_45	p__Proteobacteria;c__Gammaproteobacteria;o__Pseudomonadales;f__Pseudomonadaceae;g__Pseudomonas
	OTU_50	p__Proteobacteria;c__Betaproteobacteria;o__Methylophilales;f__Methylophilaceae;
	OTU_67	p__Actinobacteria;c__Actinobacteria;o__Pseudonocardiales;f__Pseudonocardiaceae;
	OTU_68	p__Actinobacteria;c__Actinobacteria;o__Micrococcales;f__Microbacteriaceae;
	OTU_1252	p__Proteobacteria;c__Alphaproteobacteria;o__Rhizobiales;f__Hyphomicrobiaceae;g__Devosia
	OTU_44	p__Bacteroidetes;c__Sphingobacteriia;o__Sphingobacteriales;f__Chitinophagaceae;g__Niastella
	OTU_88	p__Proteobacteria;c__Alphaproteobacteria;o__Rhizobiales;f__Phyllobacteriaceae;g__Mesorhizobium
	OTU_5	p__Actinobacteria;c__Actinobacteria;o__Micrococcales;f__Cellulomonadaceae;g__Cellulomonas
	OTU_65	p__Proteobacteria;c__Alphaproteobacteria;o__Rhizobiales;f__Rhizobiaceae;g__Rhizobium
	OTU_212	p__Proteobacteria;c__Betaproteobacteria;o__Burkholderiales;f__Comamonadaceae;g__Hydrogenophaga
	OTU_83	p__Proteobacteria;c__Alphaproteobacteria;o__Rhizobiales;f__Hyphomicrobiaceae;g__Devosia
	OTU_147	p__Proteobacteria;c__Alphaproteobacteria;o__Rhizobiales;f__Bradyrhizobiaceae;
	OTU_392	p__Proteobacteria;c__Alphaproteobacteria;o__Sphingomonadales;f__Sphingomonadaceae;g__Sphingobium
	OTU_242	p__Proteobacteria;c__Alphaproteobacteria;o__Rhizobiales;f__Hyphomicrobiaceae;g__Devosia
	OTU_165	p__Proteobacteria;c__Gammaproteobacteria;o__Pseudomonadales;f__Moraxellaceae;
	OTU_235	p__Proteobacteria;c__Gammaproteobacteria;o__Xanthomonadales;f__Xanthomonadaceae;g__Pseudoxanthomonas
	OTU_194	p__Proteobacteria;c__Alphaproteobacteria;o__Sphingomonadales;f__Sphingomonadaceae;g__Novosphingobium
	OTU_64	p__Proteobacteria;c__Alphaproteobacteria;o__Rhizobiales;f__Rhizobiaceae;g__Shinella
	OTU_641	p__Proteobacteria;c__Alphaproteobacteria;o__Rhizobiales;f__Phyllobacteriaceae;
	OTU_148	p__Proteobacteria;c__Alphaproteobacteria;o__Rhizobiales;f__Hyphomicrobiaceae;g__Devosia
	OTU_85	p__Proteobacteria;c__Alphaproteobacteria;o__Sphingomonadales;f__Sphingomonadaceae;g__Sphingopyxis
	OTU_223	p__Actinobacteria;c__Acidimicrobiia;o__Acidimicrobiales;f__Iamiaceae;g__Iamia
	OTU_69	p__Actinobacteria;c__Actinobacteria;o__Propionibacteriales;f__Nocardioidaceae;g__Nocardioides
	OTU_141	p__Actinobacteria;c__Thermoleophilia;o__Solirubrobacterales;f__Gsoil-1167;

	OTU_174	p__Actinobacteria;c__Actinobacteria;o__Corynebacteriales;f__Mycobacteriaceae;g__Mycobacterium
T0|T1|T2-K	OTU_97	p__Actinobacteria;c__Actinobacteria;o__Propionibacteriales;f__Nocardioidaceae;
	OTU_3	p__Proteobacteria;c__Alphaproteobacteria;o__Rhizobiales;f__Rhizobiaceae;g__Rhizobium
	OTU_8	p__Actinobacteria;c__Actinobacteria;o__Micrococcales;f__Micrococcaceae;g__Pseudarthrobacter
	OTU_1	p__Firmicutes;c__Bacilli;o__Bacillales;f__Bacillaceae;g__Bacillus
	OTU_22	p__Proteobacteria;c__Betaproteobacteria;o__Burkholderiales;f__Comamonadaceae;g__Variovorax
