# Supplementary material for: The bacterial community in potato is recruited from soil and partly inherited across generations
Source: PLoS One. 2019 Nov 8;14(11):e0223691. doi: 10.1371/journal.pone.0223691 (PMC6839881; doi:10.1371/journal.pone.0223691)
Supplement: S8 Table — Differentially abundant rOTUs of the bacterial communities based on the sequencing data of potato tubers from the first generation (dataset 4) are shown. (PDF) [file pone.0223691.s011.pdf]

Table S8: Taxonomic classification of differentially abundant rOTU. Differentially abundant rOTUs of bacterial community sequencing data of potato tubers of the first generation (dataset 4) are shown.

Differentially abundant OTUs	Taxonomy
OTU_1	p__Firmicutes;c__Bacilli;o__Bacillales;f__Bacillaceae;g__Bacillus
OTU_10	p__Proteobacteria;c__Betaproteobacteria;o__Burkholderiales;f__Burkholderiaceae;g__Ralstonia
OTU_100	p__Proteobacteria;c__Alphaproteobacteria;o__Caulobacterales;f__Caulobacteraceae;g__Caulobacter
OTU_105	p__Proteobacteria;c__Gammaproteobacteria;o__Xanthomonadales;f__Xanthomonadaceae;g__Dyella
OTU_119	p__Proteobacteria;c__Alphaproteobacteria;o__Rhizobiales;f__Rhizobiaceae;g__Shinella
OTU_12	p__Thaumarchaeota;c__Soil Crenarchaeotic Group(SCG)
OTU_1252	p__Proteobacteria;c__Alphaproteobacteria;o__Rhizobiales;f__Hyphomicrobiaceae;g__Devosia
OTU_126	p__Bacteroidetes;c__Sphingobacteriia;o__Sphingobacteriales;f__Chitinophagaceae;g__Chitinophaga
OTU_13	p__Proteobacteria;c__Gammaproteobacteria;o__Pseudomonadales;f__Moraxellaceae;g__Acinetobacter
OTU_15	p__Proteobacteria;c__Gammaproteobacteria;o__Pseudomonadales;f__Pseudomonadaceae;g__Pseudomonas
OTU_152	p__Proteobacteria;c__Gammaproteobacteria;o__Xanthomonadales;f__Xanthomonadaceae
OTU_158	p__Firmicutes;c__Bacilli;o__Bacillales
OTU_16	p__Bacteroidetes;c__Cytophagia;o__Cytophagales;f__Cytophagaceae;g__Emticicia
OTU_17	p__Saccharibacteria
OTU_18	p__Firmicutes;c__Bacilli;o__Bacillales
OTU_180	p__Actinobacteria;c__Actinobacteria;o__Micrococcales;f__Microbacteriaceae;g__Leifsonia
OTU_195	p__Proteobacteria;c__Alphaproteobacteria;o__Rhodospirillales;f__Rhodospirillaceae;g__Dongia
OTU_2	p__Proteobacteria;c__Betaproteobacteria;o__Burkholderiales;f__Burkholderiaceae;g__Burkholderia-Paraburkholderia
OTU_20	p__Bacteroidetes;c__Flavobacteriia;o__Flavobacteriales;f__Flavobacteriaceae;g__Chryseobacterium
OTU_209	p__Proteobacteria;c__Betaproteobacteria;o__Burkholderiales;f__Alcaligenaceae
OTU_238	p__Proteobacteria;c__Alphaproteobacteria;o__Rhizobiales;f__Bradyrhizobiaceae;g__Bosea
OTU_25	p__Actinobacteria;c__Actinobacteria;o__Micrococcales;f__Micrococcaceae
OTU_265	p__Bacteroidetes;c__Sphingobacteriia;o__Sphingobacteriales;f__Chitinophagaceae;g__Parafilimonas
OTU_270	p__Actinobacteria;c__Actinobacteria;o__Streptomycetales;f__Streptomycetaceae;g__Streptomyces
OTU_275	p__Bacteroidetes;c__Flavobacteriia;o__Flavobacteriales;f__Flavobacteriaceae;g__Flavobacterium
OTU_28	p__Actinobacteria;c__Actinobacteria;o__Micrococcales;f__Microbacteriaceae;g__Agromyces
OTU_29	p__Proteobacteria;c__Gammaproteobacteria;o__Pseudomonadales;f__Moraxellaceae;g__Enhydrobacter
OTU_3	p__Proteobacteria;c__Alphaproteobacteria;o__Rhizobiales;f__Rhizobiaceae;g__Rhizobium
OTU_32	p__Actinobacteria;c__Actinobacteria;o__Corynebacteriales;f__Nocardiaceae;g__Rhodococcus
OTU_33	p__Proteobacteria;c__Betaproteobacteria;o__Burkholderiales;f__Comamonadaceae
OTU_34	p__Actinobacteria;c__Actinobacteria;o__Micrococcales;f__Micrococcaceae
OTU_35	p__Proteobacteria;c__Alphaproteobacteria;o__Caulobacterales;f__Caulobacteraceae;g__Asticcacaulis
OTU_350	p__Proteobacteria;c__Alphaproteobacteria;o__Rhizobiales;f__Xanthobacteraceae;g__Pseudolabrys
OTU_36	p__Bacteroidetes;c__Flavobacteriia;o__Flavobacteriales;f__Flavobacteriaceae;g__Flavobacterium
OTU_38	p__Proteobacteria;c__Betaproteobacteria;o__Burkholderiales;f__Oxalobacteraceae;g__Massilia
OTU_39	p__Bacteroidetes;c__Sphingobacteriia;o__Sphingobacteriales;f__Chitinophagaceae;g__Terrimonas
OTU_4	p__Firmicutes;c__Bacilli;o__Bacillales;f__Staphylococcaceae;g__Staphylococcus
OTU_40	p__Actinobacteria;c__Actinobacteria;o__Micrococcales;f__Micrococcaceae;g__Glutamicibacter
OTU_41	p__Bacteroidetes;c__Sphingobacteriia;o__Sphingobacteriales;f__Chitinophagaceae
OTU_410	p__Bacteroidetes;c__Cytophagia;o__Cytophagales;f__Cytophagaceae;g__Chryseolinea
OTU_42	p__Firmicutes;c__Bacilli;o__Bacillales;f__Paenibacillaceae;g__Paenibacillus
OTU_43	p__Bacteroidetes;c__Cytophagia;o__Cytophagales;f__Cytophagaceae
OTU_45	p__Proteobacteria;c__Gammaproteobacteria;o__Pseudomonadales;f__Pseudomonadaceae;g__Pseudomonas
OTU_47	p__Bacteroidetes;c__Sphingobacteriia;o__Sphingobacteriales;f__Sphingobacteriaceae;g__Pedobacter
OTU_48	p__Firmicutes;c__Bacilli;o__Bacillales;f__Planococcaceae;g__Lysinibacillus
OTU_49	p__Actinobacteria;c__Actinobacteria;o__Micrococcales;f__Micrococcaceae;g__Micrococcus
OTU_5	p__Actinobacteria;c__Actinobacteria;o__Micrococcales;f__Cellulomonadaceae;g__Cellulomonas
OTU_56	p__Actinobacteria;c__Actinobacteria;o__Micrococcales;f__Micrococcaceae;g__Arthrobacter
OTU_5735	p__Bacteroidetes;c__Flavobacteriia;o__Flavobacteriales;f__Flavobacteriaceae;g__Flavobacterium
OTU_58	p__Actinobacteria;c__Actinobacteria;o__Micrococcales;f__Microbacteriaceae
OTU_6	p__Proteobacteria;c__Betaproteobacteria;o__Burkholderiales;f__Comamonadaceae;g__Delftia
OTU_61	p__Bacteroidetes;c__Sphingobacteriia;o__Sphingobacteriales;f__Sphingobacteriaceae;g__Pedobacter
OTU_614	p__Proteobacteria;c__Betaproteobacteria;o__Burkholderiales;f__Burkholderiaceae;g__Cupriavidus
OTU_62	p__Bacteroidetes;c__Sphingobacteriia;o__Sphingobacteriales;f__Sphingobacteriaceae;g__Pedobacter
OTU_641	p__Proteobacteria;c__Alphaproteobacteria;o__Rhizobiales;f__Phyllobacteriaceae
OTU_69	p__Actinobacteria;c__Actinobacteria;o__Propionibacteriales;f__Nocardioidaceae;g__Nocardioides
OTU_7	p__Thaumarchaeota;c__Soil Crenarchaeotic Group(SCG)
OTU_73	p__Proteobacteria;c__Betaproteobacteria;o__Burkholderiales;f__Burkholderiaceae;g__Burkholderia-Paraburkholderia

OTU_74	p__Proteobacteria;c__Betaproteobacteria;o__Burkholderiales;f__Comamonadaceae
OTU_76	p__Bacteroidetes;c__Sphingobacteriia;o__Sphingobacteriales;f__Sphingobacteriaceae;g__Mucilaginibacter
OTU_77	p__Actinobacteria;c__Actinobacteria;o__Micrococcales;f__Microbacteriaceae;g__Rudaibacter
OTU_8	p__Actinobacteria;c__Actinobacteria;o__Micrococcales;f__Micrococcaceae;g__Pseudarthrobacter
OTU_85	p__Proteobacteria;c__Alphaproteobacteria;o__Sphingomonadales;f__Sphingomonadaceae;g__Sphingopyxis
OTU_86	p__Actinobacteria;c__Actinobacteria;o__Streptomycetales;f__Streptomycetaceae;g__Streptomyces
OTU_9	p__Actinobacteria;c__Actinobacteria;o__Micrococcales;f__Microbacteriaceae;g__Microbacterium
OTU_95	p__Actinobacteria;c__Actinobacteria;o__Streptomycetales;f__Streptomycetaceae;g__Streptomyces
OTU_97	p__Actinobacteria;c__Actinobacteria;o__Propionibacteriales;f__Nocardioidaceae
OTU_972	p__Bacteroidetes;c__Sphingobacteriia;o__Sphingobacteriales;f__Chitinophagaceae;g__Terrimonas
